# Sourcing thermotolerant poly(ethylene terephthalate) hydrolase scaffolds from natural diversity

**DOI:** 10.1038/s41467-022-35237-x

**Published:** 2022-12-21

**Authors:** Erika Erickson, Japheth E. Gado, Luisana Avilán, Felicia Bratti, Richard K. Brizendine, Paul A. Cox, Raj Gill, Rosie Graham, Dong-Jin Kim, Gerhard König, William E. Michener, Saroj Poudel, Kelsey J. Ramirez, Thomas J. Shakespeare, Michael Zahn, Eric S. Boyd, Christina M. Payne, Jennifer L. DuBois, Andrew R. Pickford, Gregg T. Beckham, John E. McGeehan

**Affiliations:** 1 Renewable Resources and Enabling Sciences Center, National Renewable Energy Laboratory, Golden, CO USA; 2 BOTTLE Consortium, Golden, CO USA; 3 Centre for Enzyme Innovation, School of Biological Sciences, University of Portsmouth, Portsmouth, UK; 4 Department of Biochemistry, Montana State University, Bozeman, MT USA; 5 Department of Microbiology and Cell Biology, Montana State University, Bozeman, MT USA; 6 National Science Foundation, Alexandria, VA USA; 7 World Plastics Association, Fontvieille, Monaco

**Keywords:** Biocatalysis, Hydrolases

## Abstract

Enzymatic deconstruction of poly(ethylene terephthalate) (PET) is under intense investigation, given the ability of hydrolase enzymes to depolymerize PET to its constituent monomers near the polymer glass transition temperature. To date, reported PET hydrolases have been sourced from a relatively narrow sequence space. Here, we identify additional PET-active biocatalysts from natural diversity by using bioinformatics and machine learning to mine 74 putative thermotolerant PET hydrolases. We successfully express, purify, and assay 51 enzymes from seven distinct phylogenetic groups; observing PET hydrolysis activity on amorphous PET film from 37 enzymes in reactions spanning pH from 4.5–9.0 and temperatures from 30–70 °C. We conduct PET hydrolysis time-course reactions with the best-performing enzymes, where we observe differences in substrate selectivity as function of PET morphology. We employed X-ray crystallography and AlphaFold to examine the enzyme architectures of all 74 candidates, revealing protein folds and accessory domains not previously associated with PET deconstruction. Overall, this study expands the number and diversity of thermotolerant scaffolds for enzymatic PET deconstruction.

## Abstract (web summary)

Enzymes have potential for recycling plastics such as PET, a polyester used in textiles and single-use packaging. Here, the authors identify and characterize additional PET-active biocatalysts and expand the number and diversity of thermotolerant scaffolds for enzymatic PET deconstruction.

## Introduction

Poly(ethylene terephthalate) (PET) is one of the most commonly discarded plastics. Given its ubiquity in consumer plastics and the relative ease of ester bond cleavage, PET is among the most well-studied polymers for chemical recycling^1–5^. For biocatalytic PET conversion, the use of hydrolase enzymes has witnessed major advances, both in terms of advancing the industrial relevance of this approach, and the discovery of natural microbial systems that respond to the presence of PET in nature^5–16^.

Multiple serine hydrolase family enzymes have been confirmed to deconstruct PET to mono(2-hydroxyethyl) terephthalate (MHET), terephthalic acid (TPA), and ethylene glycol (EG) (Supplementary Table 1), with new discoveries being reported frequently^17,18^. Most known PET hydrolases are cutinases, lipases, and carboxylesterases (Enzyme Commission 3.1.1.-)^10–12,15^. Based upon pioneering discoveries^5,6,8,12,15,17,19–24^, further efforts have aimed to identify the necessary features for PET hydrolytic activity and to improve these enzymes for industrial use^9,11,13,25–42^. Notably, the most efficient PET-degrading biocatalysts reported thus far are thermostable enzymes that exhibit optimal PET hydrolysis activity near the PET glass transition temperature (PET *T*_g_ ~65–80 °C) and to date, mostly on amorphous PET substrates. For example, the thermotolerant leaf-branch compost cutinase (LCC) has been engineered for improved amorphous PET hydrolysis^6,9,13^, with similar work on *Thermobifida* cutinases and the mesophilic *Ideonella sakaiensis* PETase, among others^8,26,27,29,34,36,43,44^.

The sequence and structural features that confer PET hydrolysis activity are not yet fully understood^3^, both within and beyond the sequence space explored thus far. Similarly, the diversity of enzymes naturally able to hydrolyze PET remains unclear. To address these questions, Danso et al. applied a Hidden Markov Model (HMM) to search metagenomic databases for potential PET hydrolases. They identified 504 putative PET hydrolases, based on known sequences at the time^17^. They proposed that PET hydrolysis activity is likely quite rare in nature. As these authors discussed, there remains an urgent need to further develop the suite of known PET-active enzymes from natural diversity^10,15,17^.

To that end, the current study aims to expand the catalog of thermotolerant PET hydrolase scaffolds available for future enzyme discovery and engineering. We combined an HMM approach with machine learning (ML) to identify PET hydrolases and predict the temperature where the enzymes would be optimally active based on sequence. From this analysis, we selected 74 putative thermotolerant PET hydrolases for experimental screening, sourced from seven distinct phylogenetic groups, including several from which no PET hydrolysis activity has been previously reported to our knowledge. Expression and purification trials for each enzyme were conducted, and the proteins successfully expressed were screened for amorphous PET hydrolysis as a function of pH and temperature. For the best-performing enzymes from each group, we conducted thermal characterization to measure the melting temperature (T_m_). To examine substrate selectivity, which is critical for applications of PET hydrolases to semi-crystalline post-consumer PET waste^45,46^, we performed time course deconstruction reactions using crystalline PET powder, amorphous PET powder, and amorphous PET films as substrate to ascertain differences in reactivity as a function of substrate properties. Next, we explored the relationship between enzyme charge and optimal reaction pH for each of the three PET substrates. We then integrated high-throughput X-ray crystallography and AlphaFold^47–49^ for structural characterization of all 74 enzymes to gain insights into a significantly broadened diversity of folds. Together, this work demonstrates that PET hydrolytic activity can be sourced from a wider range of natural sequence diversity than previously reported and expands the number of enzyme scaffolds for thermotolerant PET hydrolysis.

## Results

### Bioinformatics and ML enables identification of 74 diverse putative thermotolerant PET hydrolases

Similar to other successes in identifying PET hydrolases with HMM^17,50,51^, we constructed an HMM from 17 characterized enzymes that had been confirmed to exhibit PET hydrolysis activity as of December 2018 (Supplementary Table 1), and applied the HMM to search sequences in the National Center for Biotechnology Information (NCBI) non-redundant database^52^ and select thermal metagenomes from the Joint Genome Institute Integrated Microbial Genome (JGI IMG) database (Supplementary Table 2)^53^. We sought to limit the search to thermostable enzymes capable of PET hydrolysis near the PET *T*_g_. To this end, we leveraged the correlation between enzyme maximum temperatures and the optimal growth temperature (OGT) of the organism or the environment where the sequence was detected^54,55^. Hence, the HMM sequence hits were mapped to OGT data retrieved from the NCBI Bioproject database, the BacDive database^56^, and the JGI IMG metagenome sample temperature. Sequences with OGT lower than 50 °C were discarded. For sequences that could not be mapped to OGT data, we trained a ML model (ThermoProt) to discriminate between 8000 proteins from thermophiles (>50 °C) and 8000 proteins from non-thermophiles (<50 °C) using the support vector machine method with calculated amino acid features. ThermoProt demonstrated an accuracy of 86.6% in five-fold cross-validation tests (Supplementary Tables 3–7).

**Fig. 1:**
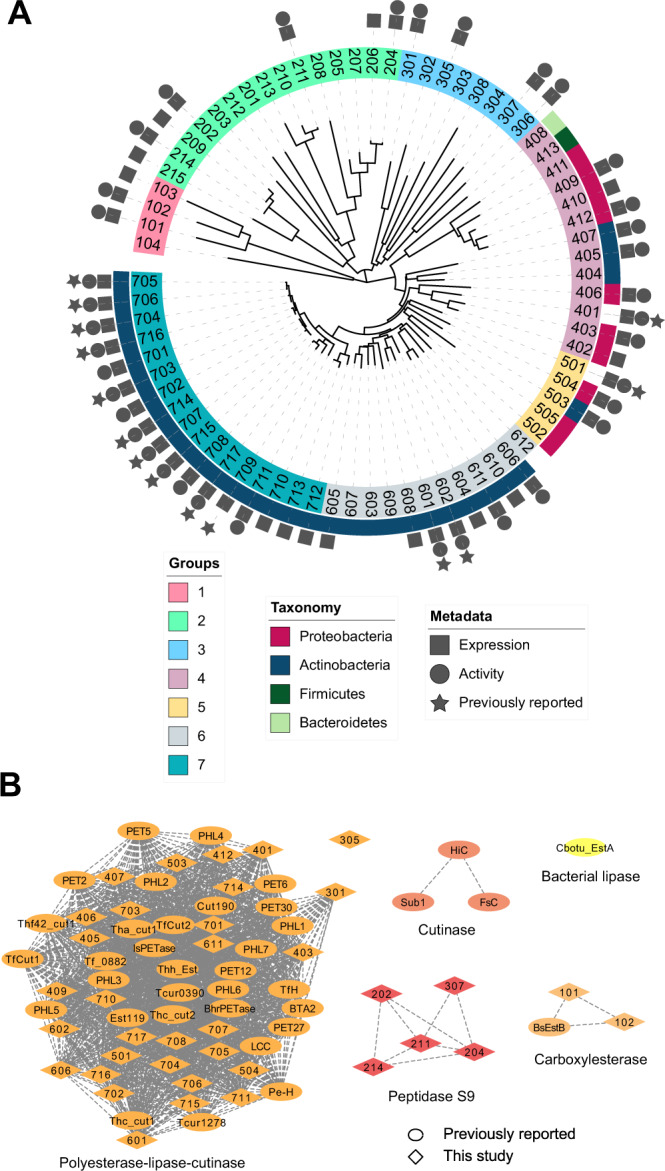
Bioinformatics and machine learning to derive PET hydrolase sequences from natural diversity. **A** PET hydrolase candidates (74 total) selected by HMM and ML shown with a minimum-evolution phylogenetic tree. Sequences retrieved from environmental (meta)genomes in JGI IMG with lower HMM scores (groups 1–3) are notably diverse compared to the sequences that comprise the rest of the tree (groups 4–7). The symbols around the tree show expression, activity, and previously reported PET activity. Full organism names and accession numbers are shown in Supplementary Table 9, and sequence identity between these 74 sequences and previously reported PETases is shown in Supplementary Table 8. A maximum-likelihood phylogenetic tree of all experimentally confirmed PET hydrolases is shown in Supplementary Fig. 1. **B** Sequence Similarity Network (SSN) of PET hydrolases with experimentally confirmed PET hydrolase activity, including sequences examined in this study and previously reported PETases. Edges represent pairwise BLAST similarity with *E*-value < 1e–10. The SSN clusters are consistent with the associated families in the ESTHER database^57^, and show that most reported PET hydrolases fall in the polyester-lipase-cutinase family. We note that these clusters are different from phylogenetic groups in (**A**). Full details of experimentally verified PET hydrolases are shown in Supplementary Tables 1 and 10.

We observed that many of the top HMM hits from the JGI IMG metagenomes were identical or very similar to hits from NCBI. To diversify the sequence search space further, we selected proteins with predicted thermostability and high HMM scores (>100, *E*-value < 8.0e−26) from the NCBI hits, but thermophile-derived proteins with relatively low scores (<55, *E*-value > 2.0e−11) from the JGI IMG hits. Consequently, 74 sequences were selected. We note that 14 of these sequences have been reported in other studies (Supplementary Tables 1, 8) to our knowledge and were retained in our assays as benchmarks. As illustrated in Fig. 1A, phylogenetic analysis showed that these 74 sequences comprise at least seven distinct phylogenetic groups, with the more diverse JGI IMG sequences forming three clades (which we termed groups 1–3) that are clearly separate from the NCBI sequences. The NCBI sequences form two clades (which we termed groups 6 and 7) and two paraphyletic groups (termed groups 4 and 5) (Fig. 1A). Based on these results, the 74 PET hydrolase candidate sequences were assigned identification numbers according to these phylogenetic groups (101 and 102 in group 1, 201 and 202 in group 2, and so on). The full list of candidate sequences is provided in the Source Data file and an annotated description with accession numbers for each is provided in Supplementary Table 9.

Upon classifying the sequences according to families from the ESTHER database^57^, results reveal that all candidate sequences in groups 4–7 with high HMM scores (>100) belong to the polyesterase-lipase-cutinase family, along with nearly all previously reported PET hydrolases, and are associated with carboxyl ester hydrolase (3.1.1.-) and cutinase (3.1.1.74) activities (Supplementary Fig. 1, Supplementary Table 10)^58^. However, the sequences derived from lower HMM scores (groups 1–3) diverge from canonical PET hydrolases and are associated with distant families, including peptidases (3.4.-.-). A sequence similarity network (Fig. 1B), plotted at a level of stringency sufficient to subdivide the sequence set into functional families, demonstrates the clustering of currently known and group 5–7 candidate PET hydrolases in the polyesterase-lipase-cutinase family, and the divergence of candidate sequences from groups 1–3.

### Screening on amorphous PET shows that PET hydrolysis activity is distributed among all seven phylogenetic groups

The 74 enzymes were expressed in *Escherichia coli* with each putative PET hydrolase gene codon-optimized and cloned into a pET21b(+) plasmid with a C-terminal hexa-histidine epitope tag, as detailed in the Methods and in the Supplementary Information. Given the diversity of enzymes to be expressed and purified, we adopted a 4-stage expression screening approach that varied *E. coli* expression strains, growth medium composition, incubation temperature and duration, induction protocol, and other relevant expression parameters, as described in the Methods and in the Supplementary Information. Enzyme purification followed a standardized protocol of affinity chromatography, buffer exchange, and size exclusion chromatography as described in Supplementary Methods. Supplementary Table 11 details the expression strategies that enabled the production of 51 of the 74 enzymes, and Supplementary Fig. 2 shows the expression yield for each enzyme.

We employed a comprehensive, semi-quantitative screening assay to first detect PET hydrolytic activity from each enzyme. In this initial activity screen, we employed commercially available amorphous PET film from Goodfellow, thereby enabling inter- and intra-study comparisons^3,25^. All reactions were conducted for 96 h at an enzyme loading of 0.7 mg enzyme/g PET and a substrate loading of 2.9%. The aromatic reaction products, bis(2-hydroxyethyl) terephthalate (BHET), MHET, and TPA, were quantitated using ultra-high-performance liquid chromatography up to a product concentration of 500 mg/L accounting for dilution, above which the calibration curve was outside of the linear range. For this substrate loading, the upper limit of quantitation of the product corresponds to a maximum extent of conversion of 2.1% by mass. Aromatic product release concentrations, relative to background aromatic product release detected in no-enzyme control reactions at each pH and temperature, are presented throughout. For comparison to the state-of-the-art from the PET hydrolase literature, we also tested four thermophilic PET hydrolases, the LCC wild-type enzyme^6,28^ two improved mutant variants (ICCG and WCCG)^13^, and *T. fusca* cutinase BTA-1^27^, to serve as benchmark datasets. We also tested representative mesophilic PET hydrolases, including the PETase wild-type enzyme from *I. sakaiensis*^8^ and an improved double mutant variant (W159H/S238F)^31,59^. The 6 benchmark enzyme sequences are provided in the Source Data file and accession numbers are in Supplementary Table 9. The ICCG variant of LCC is reported as a control for all experiments.

**Fig. 2:**
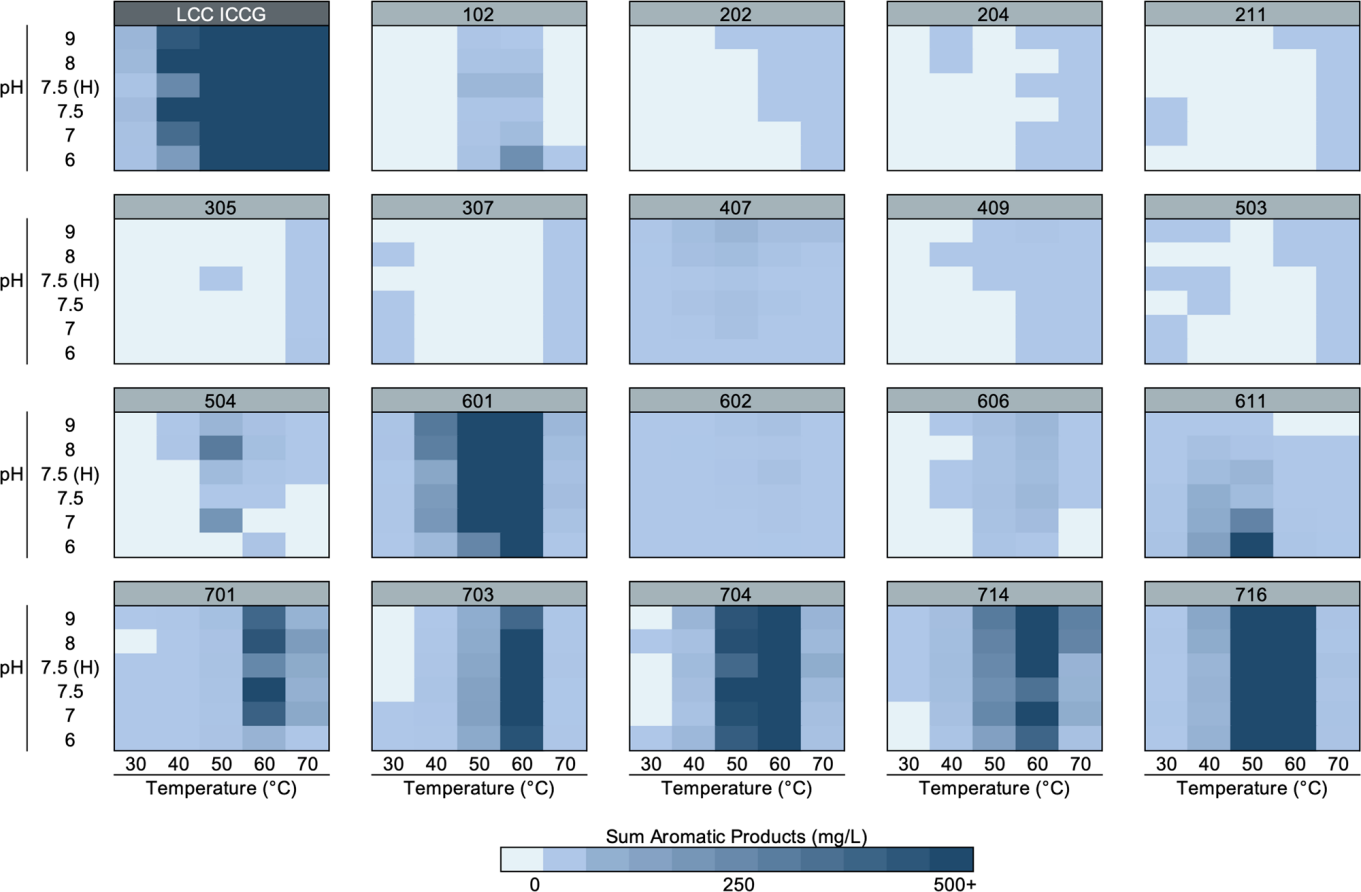
Enzyme activities. Heat map profiles of pH and temperature screening for hydrolytic activity on amorphous PET film by a diverse selection of 19 candidate enzymes and a positive control enzyme, LCC ICCG. The heat map gradient indicates the extent of measured product release up to 500 mg/L of total aromatic products after 96 h reaction time, and is reported as the average of reactions performed in triplicate (*n* = 3). Each heat map displays the reaction conditions utilized (citrate at pH 6.0, NaH_2_PO_4_ at pH 7.0, NaH_2_PO_4_ at pH 7.5, HEPES (H) at pH 7.5, bicine at pH 8.0, and glycine at pH 9.0), and reaction temperature (30, 40, 50, 60, or 70 °C). The heat maps for all other enzymes tested on amorphous PET film are shown in Supplementary Fig. 3. Source data are provided as a Source Data file.

Figure 2 shows illustrative heat maps of total aromatic product release across 30 reaction conditions using amorphous PET film as substrate for the 19 best-performing enzymes from each of the seven phylogenetic groups, alongside the ICCG variant of LCC. Supplementary Fig. 3 contains the full screening data for all 51 expressed and purified candidate enzymes and the 6 benchmark PET hydrolases. At least one enzyme from each of the phylogenetic groups shown in Fig. 1 exhibited measurable PET hydrolysis activity. Overall, 37 enzymes were found to be active for PET hydrolysis at levels above the lower limit of aromatic product quantitation, while 14 of the 51 enzymes did not exhibit any detectable PET hydrolytic activity. Figure 2 shows that enzymes in groups 5–7 exhibited the highest detected activity. This is not surprising given that most of the enzyme discovery efforts to date for PET hydrolases have identified enzymes belonging to the polyesterase-lipase-cutinase family, to which the enzymes in groups 5–7 also belong^11,12,17^. Groups 1 and 4 also exhibited appreciable PET hydrolysis activity, while groups 2 and 3 displayed only minimal activity above the no-enzyme control background. Overall, this screening highlights 23 thermostable enzymes that have not been previously reported, to our knowledge, and that exhibit PET hydrolase activity beyond the 36 previously reported enzymes at the time of writing this manuscript (Supplementary Table 8).

As shown in Fig. 2, there is a breadth of activity across the pH and temperature ranges studied, with activity of at least one enzyme in every condition tested. For the four enzymes that exhibited optimal or near optimal activity at pH 6.0 (102, 611, 702, 715), we further extended the pH screen. As shown in Supplementary Fig. 4, the ICCG variant of LCC is active in buffered medium with a pH as low as 5.0, while 102 was not active at pH below 6.0, and 611, 702, and 715 all exhibit detectable activity at pH <6.0.

### Characterization of the best-performing enzymes highlights reactivity differences as a function of substrate

We were also interested to learn if the best-performing enzymes from each phylogenetic group would exhibit different reactivity profiles as a function of PET substrate. For these comparisons, we used two commercially available substrates that have been thoroughly characterized^59^, namely a crystalline Goodfellow PET powder and the same Goodfellow amorphous PET film used for screening. This set included 12 enzymes selected to represent a diverse group for which the highest extents of conversion were observed during screening, and hydrolysis reactions utilized the single best reaction condition identified during screening on amorphous PET film (Supplementary Figs. 5, 6). These reactions proceeded for 168 h to capture effects due to enzyme stability. As shown in Supplementary Fig. 5, the control enzyme (LCC ICCG) and several group 7 enzymes (701, 704, 714, 716) exhibited higher activity on amorphous PET film, consistent with prior work^25,37,60–62^. However, we also identified enzymes with higher activity on crystalline PET powder compared to amorphous PET film (Supplementary Fig. 5), which has not previously been reported for wild-type thermophilic PET hydrolases, to our knowledge. Additional comparisons of the 168 h reactions are in Supplementary Fig. 6, Supplementary Tables 12 and the Source Data file show the corresponding reaction conditions employed in these experiments and the data, respectively.

**Fig. 3:**
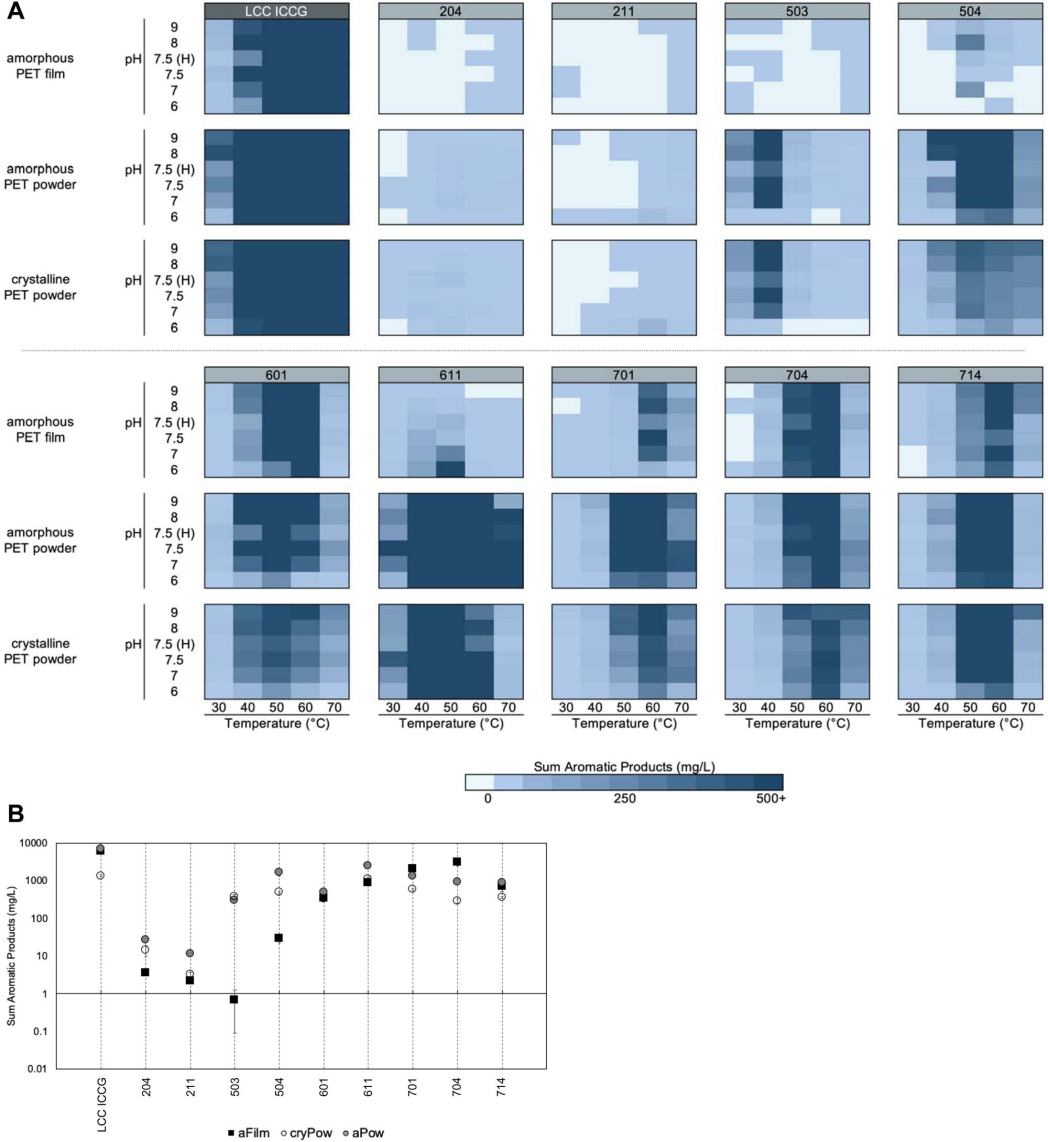
Substrate selectivity varies across PET morphologies. **A** Heat map profiles of pH and temperature screening for hydrolytic activity on 3 PET substrate morphologies, the same amorphous PET film presented in Fig. 2, as well as an amorphous PET powder and a crystalline PET powder, using a subset of 9 candidate enzymes and positive control enzyme, LCC ICCG. The heat map gradient indicates extent of measured product release up to 500 mg/L of total aromatic products after 96 h reaction time, and is reported as the average of reactions performed in triplicate (*n* = 3). Each heat map displays the reaction conditions utilized (citrate at pH 6.0, NaH_2_PO_4_ at pH 7.0, NaH_2_PO_4_ at pH 7.5, HEPES (H) at pH 7.5, bicine at pH 8.0, and glycine at pH 9.0), and reaction temperature (30, 40, 50, 60, or 70 °C). The heat maps for all other enzymes tested on the 3 PET substrate morphologies are shown in Supplementary Fig. 6. Source data are provided the a Source Data file. **B** Log-plot of the sum of aromatic products measured after 168 h reaction time using amorphous PET film (aFilm, black squares), crystalline PET powder (cryPow, open circles) and amorphous PET powder (aPow, gray circles) as substrates. Reaction conditions used for time course experiments correspond to the pH and temperature resulting in the highest product release observed in amorphous PET film screening reactions, which are listed in Supplementary Table 13. Ratios of product release observed from hydrolysis reactions for each PET substrate morphology pairwise comparison, demonstrating differences in substrate selectivity for each selected enzyme is presented in Supplementary Fig. 9. For all enzymatic reactions shown in **A**, **B**, the enzyme loading was 0.7 mg enzyme/g PET and the solids loading was 2.9% (29  g/L). The reaction products were quantified with UHPLC, and the results show the sum of aromatic products, including BHET, MHET, and TPA. All reactions were conducted in triplicate (*n* = 3). Error bars represent standard deviation and are centered on the average of the three reaction measurements. Source data are provided as a Source Data file.

Given that the hydrolytic activity on crystalline PET powder was higher than expected, for a selection of 18 candidate enzymes, including a subset of 9 of the 12 selected enzymes above, we repeated the screening experiment over 30 reaction conditions using the crystalline PET powder from Goodfellow, as well as an amorphous PET powder with the same particle size distribution profile as the crystalline powder, to control for accessible substrate surface area. Detailed characterization of the amorphous powder is described in the Methods and in the Supplementary Information. As shown in Fig. 3A and Supplementary Table 13, the optimal reaction conditions identified for each enzyme varies with each specific substrate morphology (Supplementary Fig. 7). Additional 168 h time course reactions were performed for the selected enzymes also using the amorphous PET powder, comparing the single best reaction condition from amorphous PET film screening, even though this is not necessarily the best reaction condition shared across all substrate morphologies (Supplementary Fig. 8). We observed that most enzymes demonstrate the highest levels of PET hydrolysis on the amorphous powder substrate (Fig. 3B). This is not unexpected and aligns with process conditions recommended for optimal hydrolysis reported in the previous studies^13^. Despite this, especially when comparing across conditions, we observe 3 enzymes from this selected set that demonstrate higher extents of hydrolysis for crystalline powder compared to either amorphous powder or amorphous film (enzymes 503, 602, and 711) (Supplementary Fig. 9, Supplementary Table 13). Also of note are enzymes with better hydrolytic performance on amorphous film compared to amorphous powder (enzymes 701 and 704) (Supplementary Fig. 9, Supplementary Table 13).

Of the total expressed and purified enzymes, 20 were of sufficient yield and solubility for thermostability analysis by differential scanning calorimetry (DSC), including at least one member from each of the seven distinct phylogenetic groups, as shown in Supplementary Table 14. Enzyme 306 exhibited the highest *T*_m_ (92.6 °C) of all 20 enzymes analyzed, including wild-type LCC.

### Structural characterization highlights diversity of PET-active enzymes

Given the range of sequence diversity captured in this work (Fig. 1B) and the opportunities to develop structure-function relationships across a broad group, we conducted comprehensive crystallization screening, resulting in eight high-resolution X-ray structures for enzymes 202 (7QJM), 306 (7QJN), 606 (7QJO), 611 (7QJP), 702 (7QJQ), 703 (7QJR), 705 (7QJS), and 711 (7QJT) at resolutions extending between 1.43–2.19 Å (Supplementary Table 15). As we screened enzymes more divergent from those originating from *I. sakaiensis, Thermobifida*, and LCC, the success rate of crystallization hits fell. Given that PET-active representatives were identified in all seven phylogenetic groups, we also employed AlphaFold^47^ to interrogate the structural diversity of all 74 enzymes (Supplementary Figs. 10–12) to better understand structural features across the entire cohort for PET-active and inactive enzymes.

**Fig. 4:**
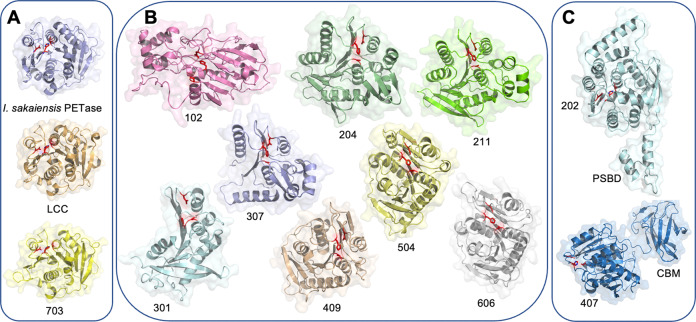
Structural diversity of PET-active and representative enzymes from phylogenetic groups. All structural models are shown to scale, rendered as cartoons with transparent accessible surface areas and putative active sites highlighted with the Ser-His-Asp catalytic triad in red sticks. **A** PET hydrolase scaffolds identified from mesophilic (*top, I. sakaiensis* PETase, PDB ID 6EQE^31^) and thermophilic (*middle*, LCC, PDB ID 4EB0^28^, and *bottom*, *T. fusca* cutinase 1 DSM44342 (703; PDB ID 7QJR)) sources occupy a narrow structural space with highly conserved α/β hydrolase folds. **B** A selection of representatives from more distant phylogenetic groups reveals multiple additional and alternative structural features with substantial increases (102) and reductions (307) in the core fold. **C** Several additional distinct domains were revealed, including a Peripheral Subunit-Binding Domain (PSBD) and a Family 35 carbohydrate binding module (CBM).

As shown in Fig. 4A, representatives of known PET hydrolase enzymes, such as those in groups 5–7, share highly similar structures. However, in groups 1–4, the expanded primary sequence diversity correlates with a large increase in structural diversity, including large core deletions, modifications, and substantial fold extensions or additions (Fig. 4B). Overall, this group of enzymes spans molecular weights ranging from 13 to 55 kDa (*I. sakaiensis* PETase is ~27 kDa) and isoelectric points from 4.3 to 9.7 (Supplementary Table 9).

### Surface residue modifications provide functional diversity while maintaining a conserved catalytic core

The group 5–7 enzymes share many common features including a highly conserved core domain with a 9-stranded β-sheet flanked by 8 or 9 α-helices. These groups represent generally the most active members of the cohort of 74, with the exception of 712 and 713, which have truncated sequences and are inactive on PET.

**Fig. 5:**
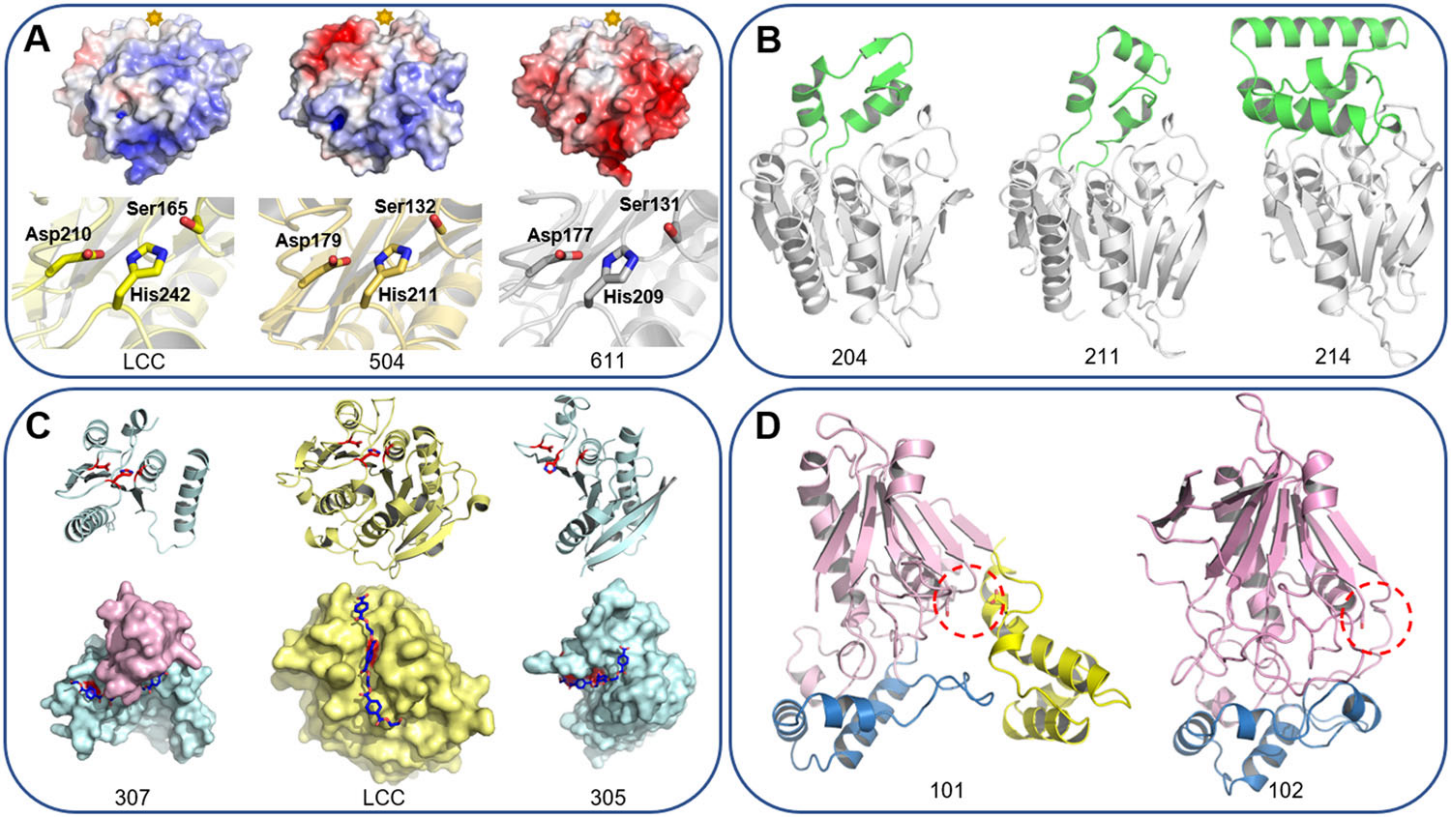
Increasing degrees of structural diversity across phylogenetic groups. **A** Conserved canonical folds with surface residue changes in groups 5 and 6. Electrostatic surface representations are colored with a gradient from red (acidic) at −7 kT/e to blue (basic) at 7 kT/e (where k is Boltzmann’s constant, *T* is temperature, and e is the charge on an electron). The general location of active site cleft is indicated with a star. Known (LCC) and predicted catalytic triad residues are shown as stick representations in the corresponding images below. **B** Accessory lid domains in group 2 enzymes. Examples of alternative lid domains are highlighted in green. **C** Mini-PETases are created from large core deletions to the canonical fold. LCC is shown in the middle column (yellow) as a cartoon with the catalytic triad highlighted in red, and a surface representation below with a PET trimer (blue) docked in the active site cleft. A comparison with 307 on the left (cartoon above shown without the lid domain for clarity) reveals the extent of the core deletion, removing four of the eight β-strands and corresponding helices. A comparison with 305 on the right reveals an almost complementary set of deletions. These major rearrangements generate alternative binding clefts and docking studies predict vastly different binding modes (PET trimers in blue). Superpositions of the three enzymes in this panel are depicted in Supplementary Fig. 19. **D** An alternative enzyme family for PET hydrolysis. The enzymes 101 (left) and 102 (right) are colored according to the 3-domain arrangement in the *Geobacillus stearothermophilus* carboxylesterase EST55 (PDB ID 2OGT). Both enzymes display a truncated version of the catalytic domain (pink) compared to EST55 (Supplementary Fig. 20) and have modified versions of the α/β domain (blue). Only enzyme 101 has a version of the regulatory domain, the absence of which in 102 disrupts the formation of the canonical active site (locations highlighted with red dashes). While the catalytic Ser and Glu residues are conserved between EST55 and 101 (pink and yellow sticks), there is no direct substitute for the His residue. In enzyme 102, only the catalytic Ser is position is conserved (Supplementary Fig. 20).

A comparison of LCC with enzymes 504 and 611 reveals high similarities, and almost identical active site triad geometries (Fig. 5A) making the selectivity of these two enzymes for crystalline PET powder, relative to LCC, surprising. Analysis of the surface charge distribution revealed a highly acidic patch adjacent to the active site cavity of enzyme 504 compared to LCC, while 611 displays an exceptionally acidic surface extending around multiple faces, in stark contrast to canonical PET hydrolases that are generally more positively charged on the solvent-exposed surface (Fig. 5A). This correlates with an isoelectric point of 4.3 for enzyme 611, compared to 9.3 for LCC.

#### Structural features provide clues for mechanism of substrate selectivity

In search of a mechanistic explanation for the diverse substrate selectivity behavior observed through biochemical assay, two hypotheses based on structural characterization were explored. First, the diverse surface charges, represented by a broad range of isoelectric points, may be key in mediating enzyme-substrate interactions or enzyme access to reactive sites on the surface of the substrate. For example, enzyme 611, which has a very low pI (4.3), shows a significant change in substrate selectivity across substrate morphologies under different reaction pH conditions (Supplementary Fig. 6D). To understand if enzyme pI correlates with optimal reaction pH for any of the PET substrate morphologies, optimal reaction pH was plotted against enzyme pI (Supplementary Fig. 13). No correlation is observed in our experiments, as has been previously reported for other enzymes^63^. A second hypothesis explores the active site cleft conformation and the constraints it sets for accommodating PET polymers. Computational substrate docking reveals that LCC accommodates a PET trimer deep within a cleft, which leads to a strong preference for twisting of the adjacent monomers in the polymer chain (Supplementary Fig. 14). Enzymes 504, 606, and 611 all present shallower clefts that enable the polymer chain to adopt low energy conformations where the monomer units adopt a more linear arrangement, similar to that observed in crystalline PET. These results therefore provide a potential structural rationale for the observed preferential breakdown of crystalline rather than amorphous PET by these enzymes (Supplementary Figs. 6D and 13).

### Evolution of lid and accessory domains generates additional variety

A variety of accessory domains are observed in groups 2–4, ranging from small lids that cap or partially occlude the predicted active site regions, to large independent folds connected by flexible linkers (Figs. 4C, 5B). These include a Peripheral Subunit-Binding Domain (PSBD) in enzyme 202, and a Family 35 carbohydrate binding module (CBM) in enzyme 407 (Fig. 4C). Enzyme 408 contains a putative cell wall anchor domain, and enzyme 212 contains a predicted extended transmembrane anchor (Supplementary Fig. 15).

The group 2 enzymes are peptidase-like hydrolases with sparing activity on PET, all characterized by a central core with the addition of lid domains in a variety of constructions. Examples include a mixed helical and β-sheet arrangement (204), a three-helix bundle (211), and for enzyme 214, a substantial 80-residue extended helical domain which creates a 40 Å wide flat surface platform of unknown function (Fig. 5B, Supplementary Fig. 16).

It is of particular note that the shapes of the group 2 active site clefts are also unusual. In example, enzyme 204 displays a partially covered active site (Supplementary Fig. 17). In a departure from classical PET hydrolases, the active site of 202 is completely buried in this apo crystal structure. However, the occluding helix sits on what appears to be a hinge-like structure that may have the potential to swing open to accommodate the polymer chain (Supplementary Fig. 18).

### Mini-PETases reconstitute productive active sites from only half the core domain

Enzyme 307 has a large deletion of around one half of the core domain, with only four strands in the central β-sheet compared to the typical eight or more strands found in canonical PET hydrolases (Fig. 5C, Supplementary Fig. 19). Despite the absence of four helices in the core, this enzyme remarkably retains the conserved canonical active site, which conveys a low level of PET hydrolysis activity (Supplementary Figs. 3, 5). As a result of the deletion, the 307 active site is open and docking studies predict potential electrostatic interactions that may stabilize an otherwise flexible protein following substrate binding. Docking simulations with a PET trimer reveal the potential for binding within a large open cleft, as compared to the relatively narrow groove of the LCC active site (Fig. 5C).

Enzyme 305 also displays a major deletion, but more surprisingly in the opposite half of the core compared to 307. The missing α-helical region would normally contribute half of the active site cavity and the His residue of the active site triad in the canonical fold. On closer inspection, an alternative His is positioned in the triad, reconstituting what appears to be a unique active site from the same half of the core (Supplementary Fig. 19). Both mini-PETases offer opportunities to investigate the minimal protein chain required for PET hydrolysis, and these examples offer two alternative active sites. Experimental validation of the predicted catalytic residues is still needed to fully understand the implications of these alternative active sites.

### Newly identified PET-active family members offer alternative folds, binding surfaces, and active site geometries

The group 1 enzymes exhibit a distinct fold, closer to carboxylesterases, such as the EST55 enzyme from *G. stearothermophilus* (PDB ID 2OGT)^64^ (Fig. 5D) and a previously identified mesophilic enzyme with PET activity, *Bacillus subtilis p*-nitrobenzylesterase, BsEstB^65,66^. An AlphaFold structural model reveals that the BsEstB enzyme is similar to EST55, sharing the same 3-domain architecture (catalytic, regulatory, and α/β) with conserved active site triad residues (Supplementary Fig. 20). However, enzymes 101 and 102 have comparatively large deletions in the main catalytic domain, and enzyme 102 lacks the regulatory domain entirely (Fig. 5D). These truncations are significant because in the canonical fold they contribute around one half of the active site environment, including the catalytic His and Glu residues (Supplementary Fig. 20). Both 101 and 102 conserve the position of the catalytic Ser, but there is no equivalently positioned His in 101, and no equivalently positioned His or Glu in 102. Further experimental validation of the non-canonical predicted catalytic residues will be necessary to identify if there may be additional alternative active site residues involved in PET hydrolysis.

## Discussion

Enzymes capable of PET hydrolysis have been identified thus far from a relatively narrow sequence space^5,10–12^, and therefore are unlikely to fully encompass the natural diversity that can catalyze this reaction. Using bioinformatics and ML to gather sequences from environmental and cultivar genomes, we discovered distinct enzymes that hydrolyze PET, likely all via a serine hydrolase mechanism based on almost universally complete conservation of the catalytic triad, but with different active site architectures, including several variations that will benefit from more study. Many of these rearrangements and adaptations create alternative active site clefts, gorges, and planes, which may provide a useful diversity of structural motifs to achieve efficient interfacial biocatalysis for PET deconstruction. Furthermore, distinct differences in surface charge and in binding mode provide tractable parameters for enzyme engineering to develop biocatalysts with high selectivity for different morphologies of PET, as commercially available model substrates are not necessarily representative of PET waste streams.

The JGI IMG sequences in groups 1–3 yielded low alignment scores with the PET hydrolase HMM (Supplementary Table 9), though several of these sequences demonstrated hydrolytic activity on PET, despite being markedly diverse relative to canonical PET hydrolases. This finding suggests that the distribution of currently known PET hydrolases, which are largely limited to the polyesterase-lipase-cutinase family (Fig. 1B), may result from biases of sequence similarity and HMM methods that limit the search to a narrow sequence space within the vicinity of these first studied PET-active enzymes. To understand these limitations, we further examined the ability of HMM scores to discriminate between active PET hydrolases and inactive homologs by computing the area under the curve (AUC) of the receiver operating characteristic plot and the Spearman correlation coefficient (ρ) between HMM scores and our experimental activity data (Supplementary Fig. 21A–C). Our results indicate that the HMM scores demonstrate mediocre performance in predicting the PET hydrolase activity of putative hits (AUC = 0.581, *ρ* = 0.167). Furthermore, we investigated the distribution of amino acids at each position in a multiple sequence alignment (MSA) of active PET hydrolases and inactive homologs to identify positions that correlate with activity and, therefore, could play key roles in PET hydrolysis activity^67^. We did not find statistically significant relationships (two-sided chi-squared test of independence, *p* < 0.01) between positional variation in the MSA and activity (Supplementary Fig. 21D). This suggests that pairwise covariation and higher-order interactions that are not captured by the HMM^68^ could play dominant roles in PET hydrolase activity. Recent studies have shown that ML can successfully capture such complex pairwise interactions^68–70^. Consequently, the application of ML with our experimental activity data within a semi-supervised framework provides promise for improved prospecting of additional active PET hydrolases^71^.

Our analysis of candidates from this study already extends to some industrially relevant parameters. For example, previous studies have shown that high substrate crystallinity leads to reduced conversion extents relative to amorphous PET^9,13,25,34,37,59,72^. This has led to an emphasis on substrate pretreatment to amorphize PET^13,15^. We recently reported a techno-economic analysis and life cycle assessment of enzymatic PET recycling^45,46^. Of relevance to PET crystallinity and pretreatment, the process model included thermal extrusion, rapid quenching, and mechanical size reduction using a microgranulator to reduce the crystallinity of PET from post-consumer PET flake^13^. Sensitivity analysis indicates the potential to reduce process electricity usage by 67%, overall process energy by nearly 50%, and a savings of $0.24/kg recovered TPA if substrate pretreatment could be avoided, thus motivating an interest in enzymes with specificity to crystalline substrates. As shown in Figs. 2 and 3, several enzymes reported here preferentially deconstruct crystalline PET powder relative to some morphologies of amorphous PET, suggesting possibilities in biocatalyst development for crystalline PET deconstruction^16,45,46^, and highlighting the potential for identifying additional desirable biocatalyst characteristics from natural diversity toward the development of enzymatic PET recycling.

## Methods

### Materials

Amorphous PET film (Goodfellow Product ES30-FM-000145) and crystalline PET powder (Goodfellow Product ES30-PD-006031) were purchased from Goodfellow Corporation (USA). Percent crystallinity for each substrate has previously been reported^59^. All reagents and buffer components were acquired from Sigma-Aldrich.

### Sequence search and alignments

Environmental metagenomes (*n* = 3136) were retrieved from the Joint Genome Institute Integrated Microbial Genome (JGI IMG) database^53^ in April 2017. The metagenomes were first categorized into sub-categories (thermal springs, groundwater) as previously reported^73,74^, and only thermal spring metagenomes were considered further (Supplementary Table 2). Sequences from these metagenomes were retrieved (~38 million sequences). The National Center for Biotechnology Information (NCBI) non-redundant database^52^ was also downloaded as of 20 December 2018 (~184 million sequences). A dataset of 17 enzymes that were confirmed to exhibit PET hydrolysis activity as of 20 December 2018 was compiled (Supplementary Table 1). Sequences of the 17 PETases were retrieved and aligned with T-Coffee^75^. T-Coffee performed better in aligning the distantly related sequences, compared to MAFFT^76^, ClustalW2^77^, and MUSCLE^78^, particularly in correct placement of the catalytic Ser and His residues and the terminal Cys residues.

A profile hidden Markov Model (HMM) was constructed with the PETase alignment using the HMMER software (version 3.1b2)^79^ and putative PET hydrolases were retrieved by hmmsearch of the HMM against the retrieved NCBI and JGI IMG sequences. The NCBI search returned 2165 hits with alignment scores ranging from 100 to 442 (*E*-value: 7.7e−25 to 8.6e−129). To diversify the sequence search space, the HMM threshold was lowered for the JGI IMG search and sequences with relatively lower scores were selected. The JGI search returned 1367 hits with alignment scores ranging from 26 to 360 (*E*-value: 1.0e−2 to 1.8e−104). For organisms from which the NCBI sequence hits were derived, optimal growth temperature (OGT) data were retrieved from the NCBI Bioproject database (https://www.ncbi.nlm.nih.gov/bioproject/) and the BacDive database^56^ (https://bacdive.dsmz.de/). The sample temperatures of the JGI IMG metagenomes (Supplementary Table 2) were used as the OGT for the JGI IMG sequence hits. To limit the search to thermostable sequences, only thermophilic sequences with OGT of 50 °C or greater were selected. Among the NCBI hits, 31 were selected as thermophilic, 1777 were mesophilic and were discarded, and 353 were from organisms that could not be mapped to OGT data. The thermophilicity of these sequences that could not be mapped to OGT data was predicted with ThermoProt (*vide infra*). The final selection included 58 thermophilic sequences (predicted/OGT) from NCBI (scores: 104–442, *E*-values: 8.0e−26–8.6e−129) and 35 sequences from JGI IMG (scores: 27–35, *E*-values: 3.0e−3–2.6e−5). Redundant sequences (100% identity, excluding the predicted signal peptide region) were removed, which left 74 putative thermophilic PET hydrolases in the selection (Supplementary Table 9).

Unless otherwise stated, structure-based multiple sequence alignments were used in all analyses and were performed as follows. First, a structural alignment of all crystal structures and AlphaFold structure models presented in this work was performed with the Promals3D web server^80^. Then, all sequences to be analyzed were aligned with MAFFT using the structural alignment as constraint^76^. Sequence analyses were implemented with the Biopython package^81^.

### Prediction of thermophilicity with machine learning (ThermoProt)

From the NCBI and BacDive databases, sequence and OGT data were retrieved for 24 organisms classified as psychrophilic (<15 °C), mesophilic (25–37 °C), thermophilic (45–70 °C), or hyperthermophilic (>80 °C) (Supplementary Table 3). A separate testing set was formed of 22,299 proteins from an organism in each OGT class, and the remaining sequences (231,171) were used in training and validation. To prevent overestimation of the validation performance, the sequences were clustered at 40% sequence-identity threshold using the CD-HIT algorithm^82^. From the CD-HIT output, 40,000 sequences were selected for validation such that there were 10,000 sequences in each class, with 8000 sequences (2000 in each class) set aside for hyperparameter tuning, while the remaining 32,000 (8000 in each class) were used for training, validation, and analysis. Three categories of features were derived from the protein sequences.

#### Amino acid composition features

the relative amounts of 20 canonical amino acids in the sequence.

#### g-gap dipeptide composition

the relative amounts of the peptide, a(x)_g_b, where a and b are specific amino acids and (x)_g_ represents g amino acids of any type, sandwiched between a and b^83^. In this work, 1200 g-gap dipeptides (i.e., g = 0, 1, and 2) were tested and the top 10 were selected by their relative (Gini) importance in a random forest model. Additional g-gap dipeptides beyond 10 did not improve the random-forest classification performance.

#### Residue type and physiochemical features

in addition, 20 features that have been shown in previous works to correlate with thermal stability were selected, namely the composition of acidic, basic, non-polar, acyclic, aliphatic, aromatic, charged, and EFMR (Glu, Phe, Met, Arg) residues; the ratio of basic to acidic, non-polar to polar, acyclic to cyclic, and charged to non-charged residues^84^; the composition of tiny (Ala, Gly, Pro, Ser) and small (Thr, Asp) residues, the average maximum solvent accessible area (ASA)^85^, the ratio of (Glu + Lys) to (Gln + His)^86^, charged vs. polar composition^87^, IVYWREL (Ile, Val, Tyr, Trp, Arg, Glu, Leu) composition^88^, molecular weight, and heat capacity^89^. Supp﻿lementary Table 4 shows a full description of the 50 features derived for each sequence and the Spearman correlation coefficient between these features and the thermostability class, using the dataset of 32,000 proteins. Five machine-learning methods were tested with the Scikit-learn Python package^90^: random forests, logistic regression, Gaussian naïve Bayes, K-nearest neighbor, and support vector machine (SVM). Hyperparameters for each method were optimized with a grid search with a separate tuning dataset (8000 proteins). Four binary classifiers were tested: psychrophilic vs. mesophilic (PM), mesophilic vs. thermophilic (MT), thermophilic vs. hyperthermophilic (TH), and mesophilic vs. thermophilic/hyperthermophilic (MTH). Supplementary Tables 5–7 show the performance of the machine-learning methods with the different binary classification schemes measured over fivefold cross-validation with the training dataset (32,000 proteins, 8000 per class). All methods achieve accuracies between 68.0% and 86.6%. In addition to the accuracy, the true positive rate (recall), true negative rate (specificity), and Matthew’s correlation coefficient were also computed. The SVM method (termed ThermoProt) yielded the best performance (MTH, 86.6% accuracy) and was applied to the PETase HMM hits without OGT data to predict the thermophilicity. It is important to note that while this work was ongoing, a dataset of OGT for 21,498 microbes was published^54^, which enabled regression models that directly predict the OGT^91,92^, and the optimal catalytic temperature (*T*_opt_) of an enzyme^92,93^. These new regression methods possibly enable improved prediction of the thermotolerance of enzymes.

### Discrimination of active PETases from inactive homologs with hidden Markov Models (HMM)

Sequence data of 60 enzymes with experimentally confirmed PET hydrolase activity were compiled, comprising 36 PETases reported in other studies (Supplementary Table 1) and 24 non-redundant PETases presented in this study (Supplementary Table 11). Sequence data of 19 homologs that are experimentally reported to be inactive on PET were also compiled, comprising 15 sequences from this study (Supplementary Table 11), PET28, PET29, PET38^94^, and Cbotu_EstB^95^ reported previously. An alignment of all 79 active and inactive sequences was performed, and the alignment was split to separate sub-alignment of active and inactive sequences.

The performance of HMM in discriminating active PETases from inactive homologs was evaluated with fivefold cross-validation. The active/inactive sequences were split into five folds and the HMM was repeatedly built with the data in four folds and evaluated with the data in the left-out fold such that each fold was iteratively used in training and testing. Two methods of HMM prediction were considered. First, an HMM was built with active PETases in the training set and searched against sequences in the testing set, and the HMM alignment score of test sequences was derived as a predictive measure of PET hydrolase activity (score method). In the second method (difference method), an additional HMM was built with inactive homologs in the training set, and searched against the testing set. The difference between the HMM score obtained from the active PETase HMM and the score from the inactive homologs HMM was construed as the predictive measure of PET hydrolase activity. With the score method, it is expected that PET hydrolase activity would directly correlate with the HMM scores, while with the difference method, it is expected that active PETases would yield higher scores with the active HMM compared to the inactive HMM. Similar HMM approaches have proven remarkably successful in discriminating functional subtypes and specificities in protein families^67,96^. However, the results here indicated that HMM demonstrates low performance in discriminating PETases from inactive homologs (Supplementary Fig. 21).

In addition, the amino-acid distribution in the alignment of active PET hydrolases and inactive homologs was investigated. If a residue position plays key roles in activity, it is expected that the amino acid distribution at that position would significantly vary between actives and inactives^67^. A Chi-squared test of independence (two-sided) was performed to compare the amino-acid distribution at each position in the alignment of 60 active PETases and 19 inactive homologs. Positions with gaps in more than 90% of the sequences were removed (805 removed, 437 remaining). The test examined the null hypothesis that the amino acid distribution at a position in the alignment is significantly different between the active PETases and inactive homologs. A second test was also performed to compare the distribution of amino acid types (aliphatic: Ala, Gly, Val, Leu, Ile, Met, Cys, Pro; aromatic: Phe, Trp, Tyr, His; positive: Arg, Lys; negative: Asp, Glu; polar: Asn, Gln, Ser, Thr). The results indicated that no single position in the alignment showed statistically significant difference (*p* < 0.01) between active PETases and inactive homologs (Supplementary Fig. 21D).

### Phylogenetic analyses and sequence similarity network

Phylogenetic analyses were conducted with the MEGAX software^97,98^. For the phylogeny of 74 candidate sequences (Fig. 1A), the evolutionary history was inferred using the Minimum Evolution (ME) method^99^. The evolutionary distances were computed using the JTT matrix-based model and are in the units of the number of amino acid substitutions per site^100^. The ME tree was searched using the Close-Neighbor-Interchange (CNI) algorithm at a search level of 1^101^. The Neighbor-joining algorithm was used to generate the initial tree^102^. All ambiguous positions were removed for each sequence pair with the pairwise deletion option. A separate tree was constructed to additionally illustrate the phylogenetic relationships of 36 previously reported PET-hydrolases and the unique PET-hydrolases presented in this study (Supplementary Fig. 1) using the maximum likelihood method with 1000 replicates and the JTT matrix-based model. The initial tree for the heuristic search was obtained by applying the Neighbor-Join and BioNJ algorithms to a matrix of pairwise distances estimated using the JTT model, and then selecting the topology with superior log likelihood value. All positions with <95% site coverage were eliminated. The phylogenetic trees were visualized with the Interactive Tree of Life (iTOL) online tool^103^.

The sequence similarity network (SSN) (Fig. 1B,) was implemented with the Enzyme Function Initiative Enzyme Similarity Tool (EFI-EST)^104^. Sequences were subjected to a BLASTall pairwise search and the SSN was constructed with a threshold of 1e−10. The SSN was visualized with Cytoscape^105^.

### Amorphous PET powder production and analysis

For generation of an amorphous PET powder, 300 mm × 300 mm sheets of 0.25 mm-thick amorphous PET film (Goodfellow Product ES30-FM-000145) were first cut into 100 mm × 100 mm squares with a guillotine. These were then rolled, immersed in liquid nitrogen and cryo-cut at 2400 rpm in a SM300 cutting mill (Retsch) equipped with a stainless-steel V-rotor, a bottom sieve with 4 mm square holes, and a cyclone trap for product collection. Subsequently, this cryo-cut product was immersed in liquid nitrogen and subjected to further size reduction by cryo-milling at 18,000 rpm in a ZM200 centrifugal mill equipped with a stainless-steel 12-teeth push-fit rotor, a 0.12 mm ring sieve with trapezoidal holes, and a cyclone trap. A 200 mg sample of the cryo-milled amorphous PET powder was dried under vacuum for 30 min at 45 °C, and its particle size and shape distributions were compared to that of the purchased crystalline PET powder by dynamic image analysis using a CAMSIZER X2 (Microtrac MRB) equipped with an X-Fall module to measure the cross-sectional area and aspect ratio (Supplementary Fig. 22).

### Plasmid construction

Coding sequences were codon optimized for *Escherichia coli* str. K-12 MG1655 using a guided random approach from the OPTIMIZER server (http://genomes.urv.es/OPTIMIZER). Optimized sequences for expression of the 6 control hydrolases (wild-type *Is*PETase, mutant variant *Is*PETase (W159H/S238F), wild-type LCC, the ICCG variant of LCC, the WCCG variant of LCC, and BTA-1), and all versions of the 74 candidate enzymes were synthesized by Twist Biosciences in pET21b(+) (EMD Millipore)-based plasmids. Each construct includes a C-terminal hexa-histidine epitope tag. Sequences are provided within the Source Data file.

### Enzyme expression

For identifying soluble heterologous protein expression, BL21 (DE3) *E. coli* (NEB), OverExpressTM C41 (DE3) (Lucigen), and Lemo21 (DE3) (NEB) competent cells were used. Competent cells were transformed with pET21b(+) plasmids encoding the enzyme of interest. Single colonies from transformation were then inoculated into a starter culture of lysogeny broth (LB) media containing 100 μg/mL ampicillin and grown at 37 °C overnight. Four expression strategies were evaluated using 50 mL cultures and soluble expression was evaluated by SDS-PAGE with Coomassie staining and Western blot using primary antibody against the hexa-histidine epitope tag (Invitrogen). Using results from the 50 mL scale expression tests, the best condition was chosen for each control or candidate and scaled to 1–5  L, depending on expression level. Supplementary Table 11 details which competent cell line and expression strategy was used for each control and candidate enzyme, and the final expression level (mg enzyme/L culture) obtained for each enzyme. Details of the four strategies employed are given in Supplementary Methods.

### Enzyme purification

Harvested cells were thawed on ice and resuspended in a lysis buffer (300  mM NaCl, 10 mM imidazole, 20 mM Tris HCl, pH 8.0,) with 0.25 mg/mL lysozyme, and 12.5 U/mL DNase I. Cells were lysed using either a bead beater (BioSpec Products, Inc.) or sonication with a microtip (39% power, 20 s ON, 20 s OFF for a total of 2 min 20 s ON). Lysate was clarified by centrifugation at 40,000 × *g* for 40 min at 4 °C. Clarified lysate was filtered through a 0.45 µm PVDF membrane, then applied to a 5 mL HisTrap HP (Cytiva) affinity column using an ÄKTA Pure chromatography system (Cytiva) and eluted using a buffer comprising 300 mM NaCl, 500 mM imidazole, 20 mM Tris HCl, pH 8.0. Resulting fractions containing the protein of interest were pooled and dialyzed at room temperature (25 °C) using 3.5 kDa molecular weight exclusion membranes in an exchange reservoir at least 300 times the pooled sample volume of 300 mM NaCl, 20  mM Tris, pH 8.0 buffer. After 16–20 h of buffer exchange, samples were centrifuged and evaluated by SDS-PAGE with Coomassie staining. Pooled samples were concentrated using 3.5 kDa molecular weight cut-off spin columns and applied to a HiLoad Superdex 75 pg 16/60 (Cytiva) size exclusion column equilibrated with 300 mM NaCl, 20 mM Tris, pH 8.0 for use in screening or time course analysis. Protein in eluted fractions from affinity and size exclusion columns were assessed using SDS-PAGE with Coomassie staining and Western blot using primary antibody against the hexa-histidine epitope tag (Invitrogen). Total protein was assessed by BCA assay^106^.

Using *E. coli* strains transformed with only the empty pET21b(+) expression vector, no PET hydrolysis activity was observed using the cell lysate or using endogenous *E. coli* protein that demonstrates non-specific binding to the Ni-NTA affinity column.

### Signal peptide sequences

The presence of signal peptide sequences was predicted using SignalP 5.0^107^. From 74 putative thermophilic PET hydrolase sequences, 36 signal peptides were removed for construct synthesis. A selection of 12 truncated constructs that proved challenging to express were re-synthesized to include the native signal peptide (nSP) and compared for changes in expression and activity. Of these signal peptide-containing constructs, 7 were successfully expressed and screened, of which, only 607 could not be expressed without the native signal peptide. Sequences for the nSP-containing candidates are provided in the Source Data file. In addition, expression of the Thh_Est enzyme (710) was previously reported from an expression plasmid (pET26b(+)) containing an N-terminal pelB signal peptide^22^. Both the truncated version of 710 and the pelB-containing version (710-pelB) expressed enzyme, but neither showed activity during screening (data not shown for 710-pelB).

### Protein calorimetry (DSC)

Apparent melting temperature (*T*_m_) values for those purified enzymes that were sufficiently soluble (>0.1 mg/mL) in neutral buffer were assessed by differential scanning calorimetry (DSC). Immediately prior to DSC analysis, to ensure both mono-dispersity and an optimal buffer match, each enzyme was prepared by size-exclusion chromatography (SEC) through a HiLoad Superdex 75 pg column (Cytiva) pre-equilibrated with the DSC reference buffer comprising 50 mM NaH_2_PO_4_, pH 7.5, with either 300 mM NaCl (for 606) or 100 mM NaCl (for all other enzymes). The SEC column was calibrated with a mixture of globular protein standards (Sigma-Aldrich)— thyroglobulin (670 kDa), γ-globulin (158 kDa), albumin (67.0 kDa) and ribonuclease A (13.7 kDa)—to allow for the calculation of an apparent molecular weight (MW_app_) for each enzyme from its elution volume. Subsequently, triplicate DSC analyses, each using 0.1–0.2 mg/mL enzyme, were performed on a MicroCal PEAQ-DSC-Automated instrument (Malvern Panalytical). The temperature of the sample and reference cells was raised from 30 to 120 °C at a rate of 1.5 °C/min using low feedback. Thereafter, reference buffer subtraction, baseline correction and apparent *T*_m_ determination were performed using the instrument’s data analysis software (v1.60).

### Monomer quantitation

Analyte analysis of BHET, MHET, and TPA was performed on an Infinity II 1290 ultra-high-performance liquid chromatography (UHPLC) system (Agilent Technologies) equipped with a G7117A diode array detector (DAD). Samples and standards were injected using a volume of 0.25 µL onto a Zorbax Eclipse Plus C18 Rapid Resolution HD (2.1 × 50 mm, 1.8 µm) (Agilent Technologies) column maintained at 40 °C. The mobile phase used to separate the analytes of interest was composed of (A) 20 mM phosphoric acid in ultrapure water and (B) 100% methanol. Separation of analytes was carried out using a constant flow rate of 0.7 mL/min and a gradient program with a total run time of 3 min. The gradient program proceeded as follows: at *t* = 0 min, (A) = 80% and (B) = 20%; at *t* = 2 min, (A) = 35% and (B) = 65%; from *t* = 2.01 min until the end at *t* = 3 min, (A) = 80% and (B) = 20%. The calibration curve for each analyte was evaluated between concentrations of 1–200 mg/L with DAD detection at a wavelength of 240 nm. Ten calibration standards were used with an *R*^2^ coefficient of 0.995 or better. Calibration verification standards (CVS) for each analyte was analyzed every 12–24 samples to ensure the integrity of the initial calibration. Samples were diluted with ultrapure water for analysis and maintained at 15 °C during the analysis.

### Screening for activity on amorphous PET film

In each screening reaction, 2.9% loading by mass of an amorphous PET film (Goodfellow) was incubated with 10 µg enzyme of interest (0.7 mg enzyme/g PET), unless noted otherwise in Supplementary Table 11 due to low expression levels. Reactions were performed in polypropylene tubes containing 100 mM NaCl and 50 mM buffering agent (citrate at pH 6.0, NaH_2_PO_4_ at pH 7.0, NaH_2_PO_4_ at pH 7.5, HEPES at pH 7.5, bicine at pH 8.0, and glycine at pH 9.0) and incubated at 30, 40, 50, 60, or 70 °C. All reactions were terminated after 96 h by the addition of an equal volume of 100% methanol and PET was removed from the reaction solution. Soluble fractions were filtered through 0.2 µm nylon filters for monomer quantitation. All PET hydrolysis screening reactions were performed in triplicate.

For enzymes with peak activity at pH 6.0, an extended pH screening assay was performed using 2.9% loading by mass of amorphous PET film (Goodfellow) and 10 µg enzyme of interest (0.7 mg enzyme/g PET enzyme loading) in polypropylene tubes containing 100 mM NaCl and 50 mM citrate (pH 5.5 and pH 5.0) or 50 mM sodium acetate (pH 5.0 and pH 4.5). The reactions were again stopped at 96 h by the additional of an equal volume of 100% methanol and worked up in the same manner as described directly above.

Aromatic product release data are reported throughout relative to background aromatic product release detected in no-enzyme control reactions at each pH and temperature. Background aromatic product release for both amorphous PET film and crystalline PET powder was below the detection limit for all pH and temperature combinations tested.

### Characterization of PET hydrolysis activity on varied substrates with time resolution

Using the reaction conditions (buffer and temperature combination) where peak PET hydrolysis activity was measured from the screening assays, a selection of enzymes was further characterized over a 168 h reaction on amorphous PET film (Goodfellow), crystalline PET powder (Goodfellow), and an amorphous PET powder produced in-house through cryomilling of the Goodfellow amorphous PET film. Each reaction was performed using 2.9% by mass substrate loading and 10 µg enzyme of interest (0.7 mg enzyme/g PET). Reactions were terminated at the designated timepoint by the addition of an equal volume of 100% methanol and PET was removed from the reaction solution. Soluble fractions were filtered through 0.2 µm nylon filters for monomer quantitation. All time course experiments were performed in triplicate and samples were diluted with ultrapure water for analyte quantitation. Supplementary Tables 12, 13 provide details on the enzyme and reaction condition pairings evaluated over 168 h reaction time.

### Structure determination

For crystallography, all proteins were concentrated and sitting drop crystallization trials were set up with a Mosquito crystallization robot (SPT Labtech) using SWISSCI 3-lens low profile crystallization plates (Supplementary Methods). All crystals were cryo-protected with 20% glycerol in the crystallization solution and flash-frozen into liquid nitrogen. Diffraction data were collected at the Diamond Light Source (Didcot, UK) and automatically processed using Autoproc on ISPyB^108^. STARANISO^109^ was also used for processing anisotropic data and calculating ellipsoidal completeness. The structure was solved within CCP4 Cloud by molecular replacement with Molrep^110^ using search models created by Phyre2^111^. For 306, MR was solved with an AlphaFold structure prediction (Supplementary Fig. 11)^47^. Model buildings were performed in Coot^112^ and the structures were refined with BUSTER^113^ and REFMAC5^114^. MolProbity^115^ was used to evaluate the final models and PyMOL (Schrödinger, LLC) for protein model visualizations. Data and refinement statistics are summarized in Supplementary Table 15. The atomic coordinates have been deposited in the Protein Data Bank, and PDB IDs are included in Supplementary Table 15. Search for structural protein homologs and calculation of RMSD values were performed with the DALI server^116^. AlphaFold structure predictions were generated using the same models and inference procedure as employed in CASP14^47^. Mean pLDDT (predicted local distance difference test) over the structure was used for model ranking, and pLDDT values were written into the B-factor column of each structure file.

### Molecular docking

Molecular docking calculations were performed using the program Molecular Operating Environment (MOE)^117^. Flexible PET dimers and trimers were optimized inside a rigid host structure. Initial placement of the PET oligomer units was carried out using the Triangle Matcher approach, with subsequent refinement via molecular mechanics using the MMFF94x forcefield. The position and energy of 200 poses were optimized and their ranking was carried out based on the most favorable molecular mechanics interaction energy, E_refine. Results were discarded where the distance between the carbonyl group of a monomer unit and the serine of the catalytic triad exceeded 4 Å.

## Supplementary information

Peer Review File

Supplementary Information

## Data Availability

The data that support this study are available from the corresponding authors upon request. The atomic coordinates and structure factors have been deposited in the Protein Data Bank, (https://www.wwpdb.org/) with PDB ID codes 7QJM, 7QJN, 7QJO, 7QJP, 7QJQ, 7QJR, 7QJS, and 7QJT. AlphaFold models are available at https://github.com/beckham-lab/AlphaFold-PETase-PDBs. Genetic expression constructs for the 74 sequences have been deposited at AddGene, (https://www.addgene.org/Gregg_Beckham/). Source data are provided with this paper.

## Source data

Source data

## Code availability

The ThermoProt code and training dataset are available at https://github.com/beckham-lab/ThermoProt.
