# Supplementary material for: Sourcing thermotolerant poly(ethylene terephthalate) hydrolase scaffolds from natural diversity
Source: Nat Commun. 2022 Dec 21;13:7850. doi: 10.1038/s41467-022-35237-x (PMC9772341; doi:10.1038/s41467-022-35237-x)
Supplement: Supplementary file 2 — Supplementary Information [file 41467_2022_35237_MOESM2_ESM.pdf]

Supplementary Information for:

Sourcing thermotolerant poly(ethylene terephthalate) hydrolase scaffolds from natural diversity

Erika Erickson, Japheth E. Gado, Luisana Avilán, Felicia Bratti, Richard K. Brizendine, Paul A. Cox, Raj Gill, Rosie Graham, Dong-Jin Kim, Gerhard König, William E. Michener, Saroj Poudel, Kelsey J. Ramirez, Thomas J. Shakespeare, Michael Zahn, Eric S. Boyd, Christina M. Payne, Jennifer L. DuBois, Andrew R. Pickford, Gregg T. Beckham, and John E. McGeehan

Table of Contents

Supplementary Materials and Methods

Supplementary Figures

1 Phylogenetic tree of 36 previously reported, experimentally verified PET hydrolases and the 23 newly described from this study
2 Protein yield of enzymes screened for PET hydrolysis activity
3 Heat map data for all 51 candidate enzymes and 7 native signal peptide-containing variants screened for PET hydrolysis activity
4 Heat map data for the extended pH screen of selected PET hydrolase enzymes
5 Substrate selectivity plot comparing hydrolysis extent of amorphous PET film versus crystalline PET powder
6 Time course plots comparing product release from amorphous PET film and crystalline PET powder
7 Heat map data for 18 candidate enzymes screened on three PET substrate morphologies
8 Time course plots comparing product release from three PET substrate morphologies
9 Substrate selectivity ratios from 9 candidate enzymes presented in **Figure 3B**
10 Crystallographic and AlphaFold structural comparisons
11 Molecular replacement solution of 306 using AlphaFold
12 Structure gallery
13 Exploring the relationship between enzyme pI and optimal reaction pH
14 Docking analysis of PET comparing binding modes of enzymes LCC, 504 and 611
15 Additional appendages
16 Extended helical domain of enzyme 214 creates an unusual flat surface
17 The active site of 204
18 The buried active site of enzyme 202
19 Mini-PETase superpositions with LCC
20 Truncated carboxylesterase scaffolds
21 Performance of Hidden Markov model (HMM) in predicting PET hydrolase activity
22 Dynamic image analysis of amorphous and crystalline PET powders

Supplementary Tables

1 List of experimentally verified PET hydrolases
2 JGI IMG metagenomes from which putative sequences were derived
3 Organism and sequence dataset used in machine learning prediction of thermophilicity (ThermoProt).
4 Sequence features used in machine-learning prediction of thermophilicity (ThermoProt
5 Accuracy of binary classifiers in discriminating proteins
6 Validation performance of the SVM (ThermoProt) measured over fivefold cross-validation
7 Accuracy of ThermoProt on the separate testing dataset of 22,299 proteins.
8 Maximum sequence identity between PET hydrolases presented in this study and previously reported PET hydrolases
9 Annotated list of the 74 candidate enzymes
10 ESTHER family classification and EC number prediction of candidate sequences with experimental PET hydrolase activity
11 Expression and purification trial results for all 74 enzymes and the signal peptide-containing variants
12 Enzymes and reaction conditions tested in 168 h time course experiments for amorphous PET film and crystalline PET powder
13 Enzymes and reaction conditions tested in 168 h time course experiments for all 3 PET substrate morphologies
14 T_m values
15 Crystallographic data and model refinement statistics

Supplementary References

Supplementary Materials and Methods

Enzyme expression strategies

Multiple strategies were employed for enzyme expression with details of the final expression level (mg enzyme/L culture) obtained for each enzyme in **Supplementary Table 11**.

In strategy A, the starter culture was inoculated at a 100-fold dilution into a 2xYT medium (10 g NaCl, 10 g yeast extract, 16 g tryptone per L culture) containing 100 µg/mL ampicillin and grown at 37°C until the optical density measured at 600 nm (OD_{600}) reached 0.6-0.8. Protein expression was then induced by addition of isopropyl β-D-1-thiogalactopyranoside (IPTG) to a final concentration of 1 mM. Cells were induced at 20°C for 18 to 24 h following IPTG addition, harvested by centrifugation, and stored at -80°C until purification.

In strategy B, the starter culture was inoculated at a 100-fold dilution into a 2xYT medium containing 100 µg/mL ampicillin and grown at 37°C until the OD_{600} reached 0.6. Protein expression was then induced by addition of IPTG to a final concentration of 0.5 mM. Cells were induced at 25°C for 16 to 18 h following IPTG addition, harvested by centrifugation, and stored at -80°C until purification.

In strategy C, the starter culture was inoculated at a 1000-fold dilution into ZYP-5052 medium (1) containing 100 µg/mL ampicillin and grown at 28°C for 24 h. Cells were harvested by centrifugation and stored at -80°C until purification.

In strategy D, the starter culture was inoculated at a 500-fold dilution into ZYP-5052 medium with 0.3 M NaCl containing 100 µg/mL ampicillin and grown at 25°C for 72 h. Cells were harvested by centrifugation and stored at -80°C until purification.

Crystallization conditions

The proteins were crystallized using the following screens and conditions:

202 - JCSG-plus screen (Molecular Dimensions), G7, 15 % PEG 3350, 0.1 M succinic acid
306 - SaltRx screen (Hampton Research), E8, 1.8 M sodium phosphate monobasic monohydrate, potassium phosphate dibasic pH 5.0
606 - Structure screen (Molecular Dimensions), F5, 0.1 M Sodium HEPES pH 7.5, 70% (v/v) MPD
611 - PACT screen (Molecular Dimensions), F1, 20 % PEG 3350, 0.2 M sodium fluoride, 0.1 M Bis-Tris propane pH 6.5
702 - PACT screen (Molecular Dimensions), F8, 20 % PEG 3350, 0.2 M sodium sulfate, 0.1 M Bis-Tris propane pH 6.5
703 - PACT screen (Molecular Dimensions), F10, 20 % PEG 3350, 0.02 M sodium/potassium phosphate, 0.1 M Bis-Tris propane pH 6.5
705 - JCSG screen (Molecular Dimensions), F1, 0.05 M Cesium Chloride, 0.1 M MES pH 6.5, 30% (v/v) Jeffamine M-600
711 - JCSG screen (Molecular Dimensions), D6, 0.2 M Magnesium Chloride Hexahydrate, 0.1 M Tris pH 8.5, 20 % (w/v) PEG 8000

Supplementary Figures

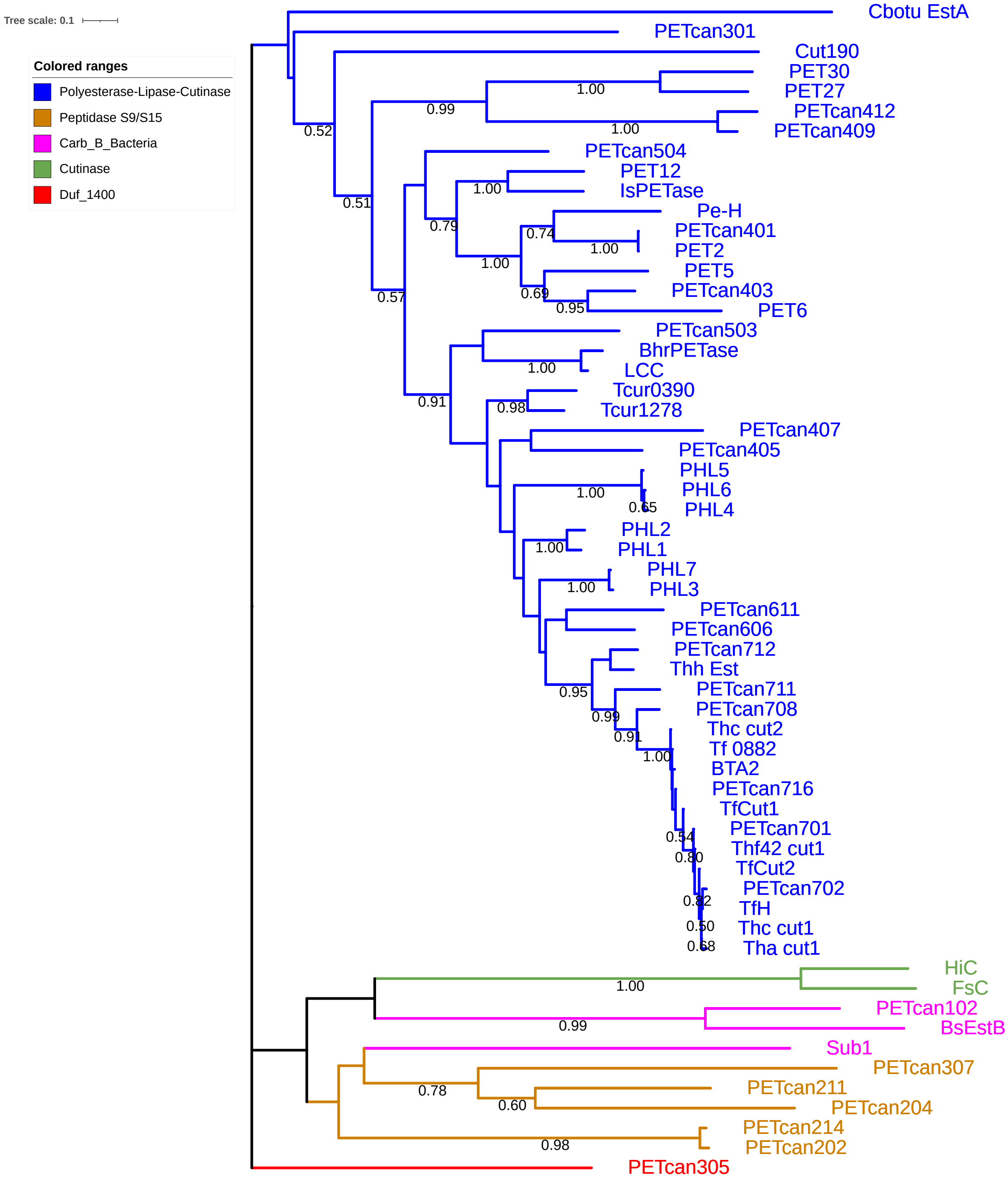

Supplementary Figure 1. Maximum likelihood phylogenetic tree of 36 experimentally confirmed PET hydrolases previously reported (**Supplementary Table 1**) and 23 from this study (**Supplementary Table 11**). Sequences in the tree are colored according to the associated families in ESTHER. Two main clades form in the tree. The first clade consists of canonical PETases in the polyesterase-lipase-cutinase family, and the second clade includes divergent sequences.

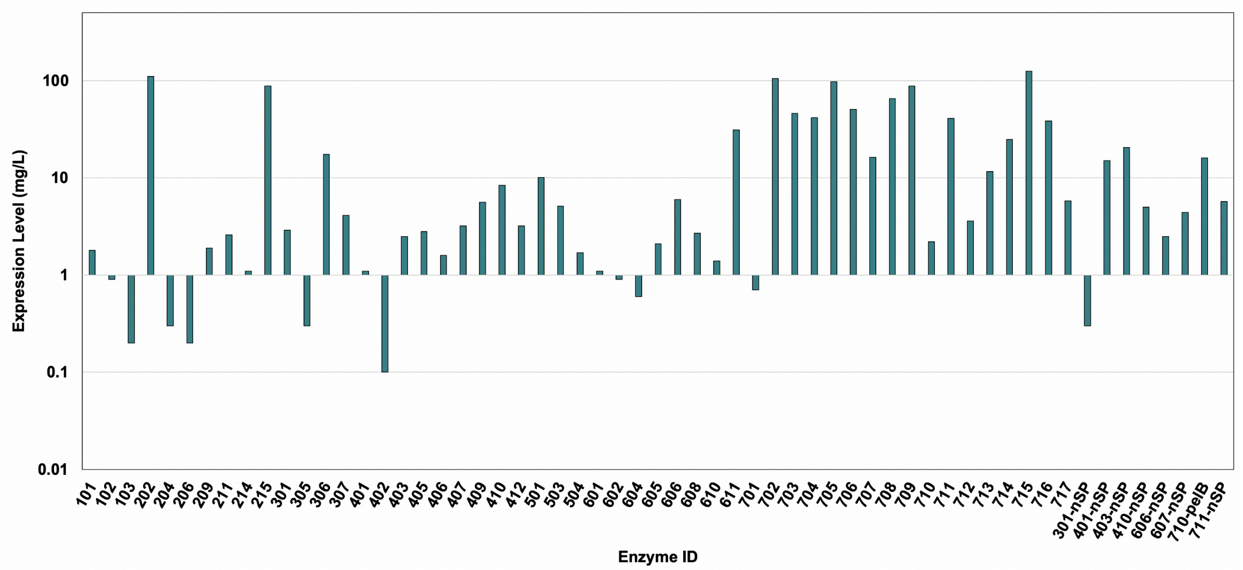

Supplementary Figure 2. Protein yield of enzymes screened for PET hydrolysis activity. Protein yield (mg enzyme / L culture) from *E. coli* cultivations is provided for each enzyme candidate expressed and screened. Insoluble enzymes and enzymes with yields below 0.1 mg/L were not screened. The numerical data are provided in **Supplementary Table 11**.

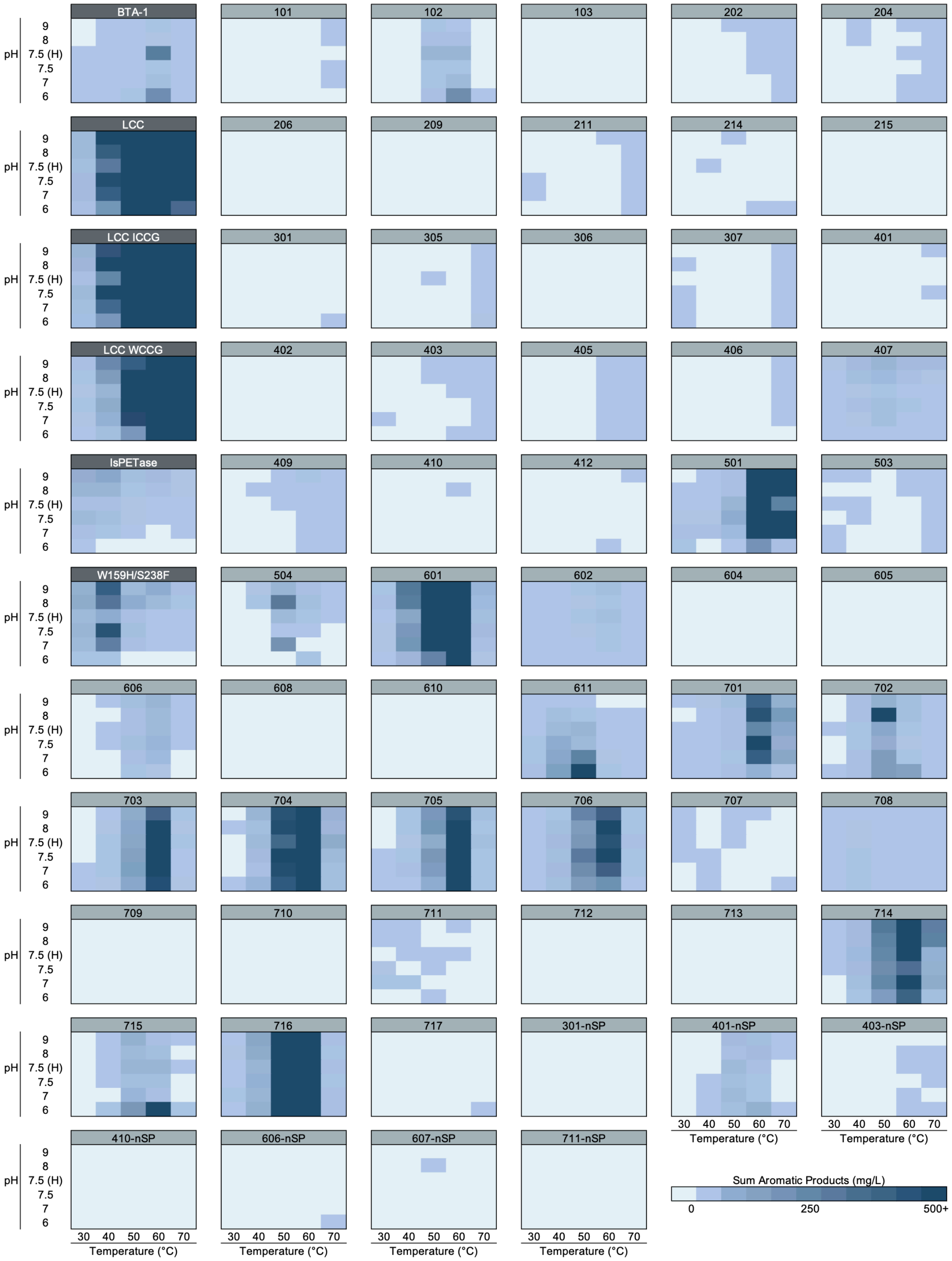

Supplementary Figure 3. Heat map data for 51 candidate enzymes, 7 native signal peptide-containing variants (nSP), and 6 benchmark enzymes from the enzymatic PET hydrolysis literature (dark grey), all screened for PET hydrolysis activity across a range of pH and temperature reaction conditions. Each heat map displays the reaction conditions utilized (citrate at pH 6.0, NaH_2PO_4 at pH 7.0, NaH_2PO_4 at pH 7.5, HEPES (H) at pH 7.5, bicine at pH 8.0, and glycine at pH 9.0), and reaction temperature (30°C, 40°C, 50°C, 60°C, or 70°C). Source data are provided as a Source Data file and can be found within as **Source Data Table D3.** Buffer compositions are provided in the Materials and Methods section.

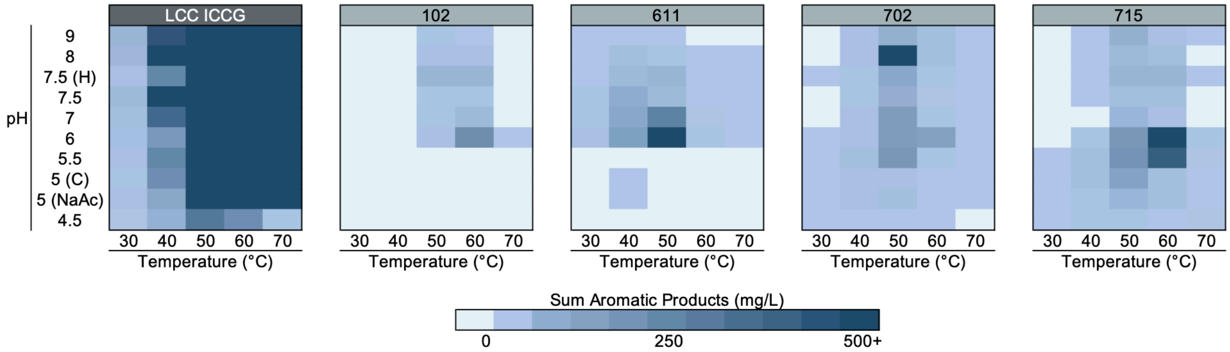

Supplementary Figure 4. Heat map data for the extended pH screen of selected PET hydrolase enzymes. Each heat map displays the reaction conditions utilized (sodium acetate (NaAc) at pH 4.5 and 5.0, citrate (C) at pH 5.0, 5.5, and 6.0, NaH_2PO_4 at pH 7.0 and 7.5, HEPES (H) at pH 7.5, bicine at pH 8.0, and glycine at pH 9.0), and reaction temperature (30°C, 40°C, 50°C, 60°C, or 70°C). Source data are provided as a Source Data file and can be found within as **Source Data Table D4**. Buffer compositions are provided in the Materials and Methods section.

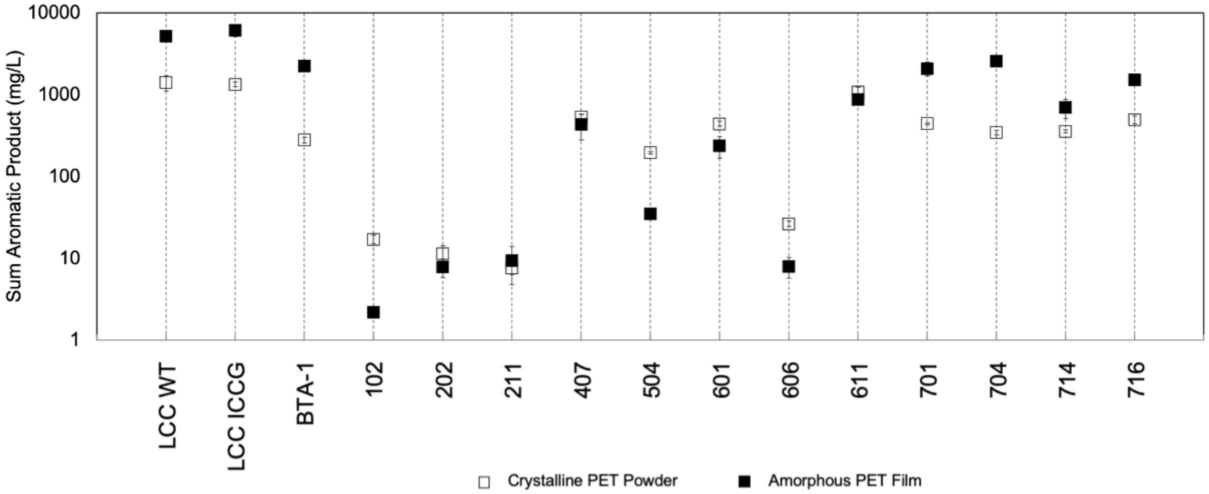

Supplementary Figure 5. Log-plot of the sum of aromatic products measured after 168 h reaction time as measured from time course experiments using amorphous PET film (black squares) and crystalline PET powder (open squares) as substrates. Reaction conditions (listed in **Supplementary Table 12)** used for time course experiments correspond to the pH and temperature resulting in the highest product release observed in amorphous PET film screening reactions. All reactions were performed in triplicate (n=3) and error bars represent the standard deviation of reaction products. Error bars are centered on the average of triplicate measurements and represented on the log-plot scale of the y-axis. Source data is provided as a Source Data file and can be found within **Source Data Table D5**.

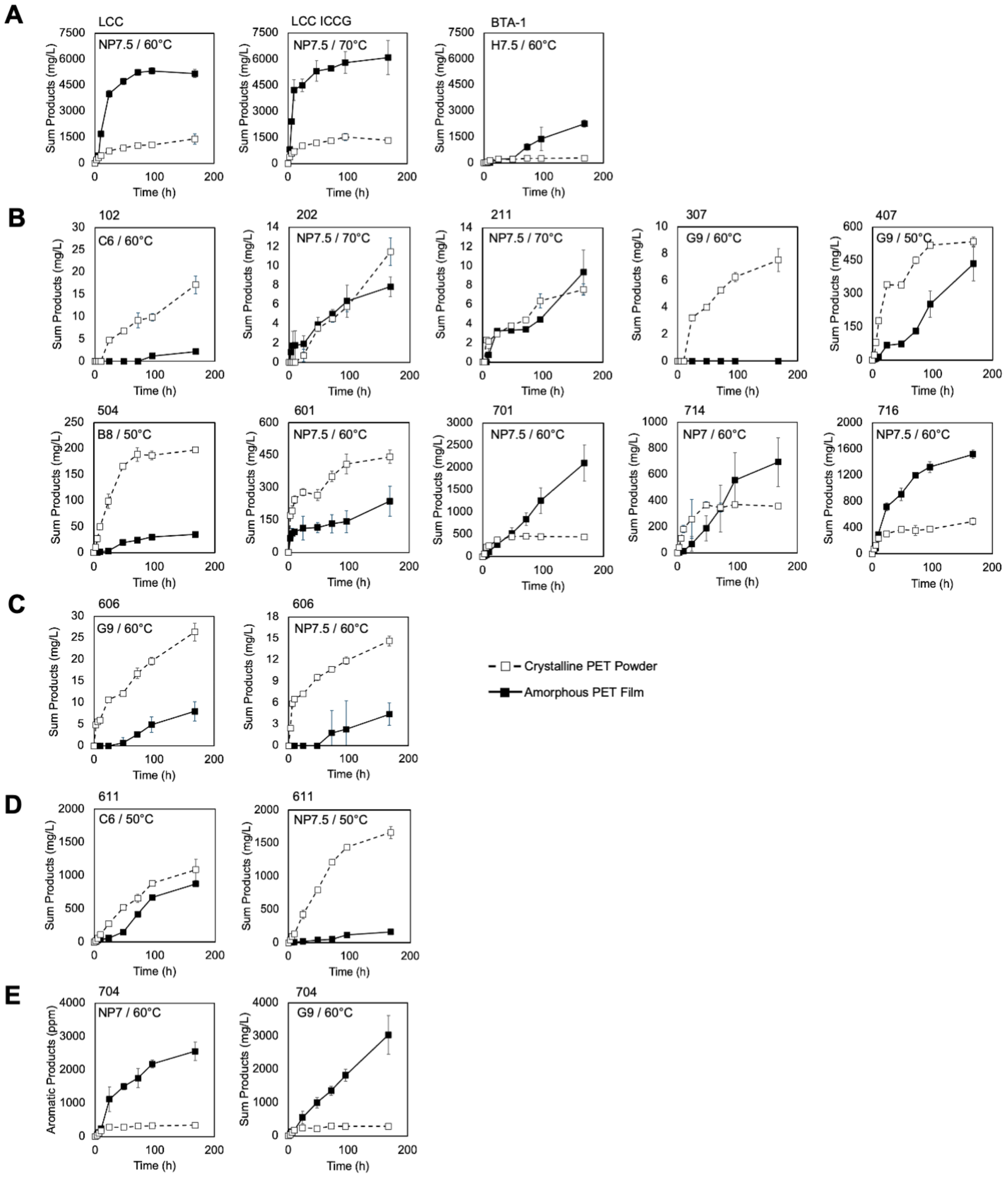

Supplementary Figure 6. Time course plots comparing aromatic product release from amorphous PET film and crystalline PET powder over 168 h reaction time. Each plot provides the reaction conditions utilized (citrate at pH 6.0 = C6, NaH_2PO_4 at pH 7.0 = NP7, NaH_2PO_4 at pH 7.5 = NP7.5, HEPES at pH 7.5 = H7.5, bicine at pH 8.0 = B8, and glycine at pH 9.0 = G9), and reaction temperature (30°C, 40°C, 50°C, 60°C, or 70°C). Error bars represent the standard deviation of reactions measured in triplicate (n=3) and are centered on the average of the three measurements. Source data are provided as a Source Data file and can be found within **Source Data Table D5**. (**A**) Comparison of benchmark enzymes using peak activity reaction conditions from screening on amorphous PET film. (**B**) Comparison of selected candidate enzymes using peak activity conditions from screening on amorphous PET film. (**C**) Comparison of two reaction conditions for enzyme 606 showing that 606 has higher activity in more alkaline reaction conditions. (**D**) Comparison of two reaction conditions for enzyme 611. Enzyme 611 is more selective for crystalline PET powder compared to amorphous PET film in both conditions tested. (**E**) Comparison of two reaction conditions for enzyme 704, showing that while 704 prefers a more alkaline reaction environment (pH 9), comparable activity is achieved even at pH 7.

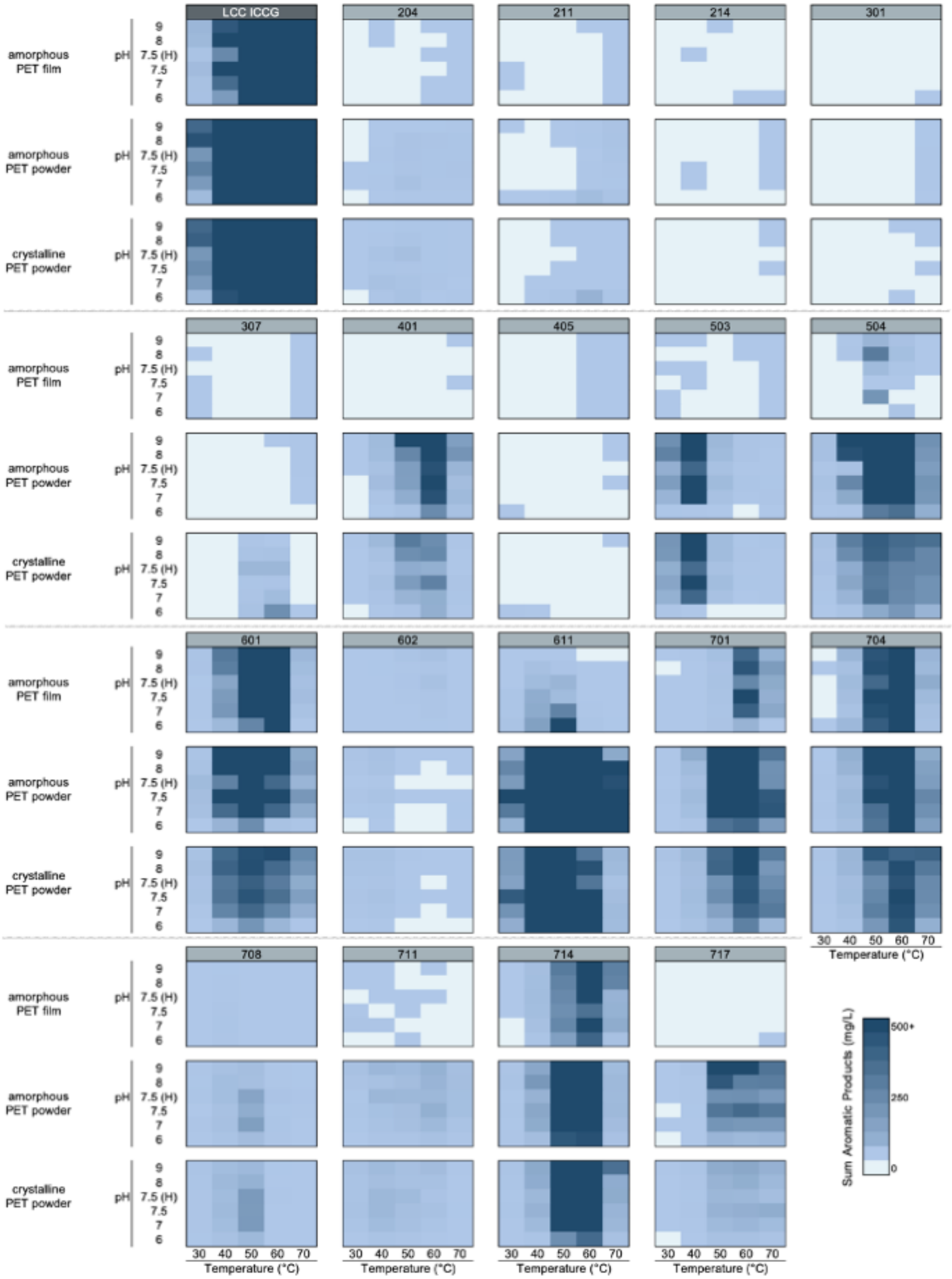

Supplementary Figure 7. Heat map profiles of pH and temperature screening for hydrolytic activity on 3 PET substrate morphologies, amorphous PET film, amorphous PET powder, and a crystalline PET powder, for a selection of 18 candidate enzymes and a positive control enzyme, LCC ICCG. The heat map gradient indicates the extent of measured product release up to 500 mg/L of total aromatic products after 96 h reaction time. Each heat map displays the reaction conditions utilized (citrate at pH 6.0, NaH_2PO_4 at pH 7.0, NaH_2PO_4 at pH 7.5, HEPES (H) at pH 7.5, bicine at pH 8.0, and glycine at pH 9.0), and reaction temperature (30°C, 40°C, 50°C, 60°C, or 70°C). Quantitative data for all enzymes screened are provided in a Source Data file and can be found within **Source Data Table D6**.

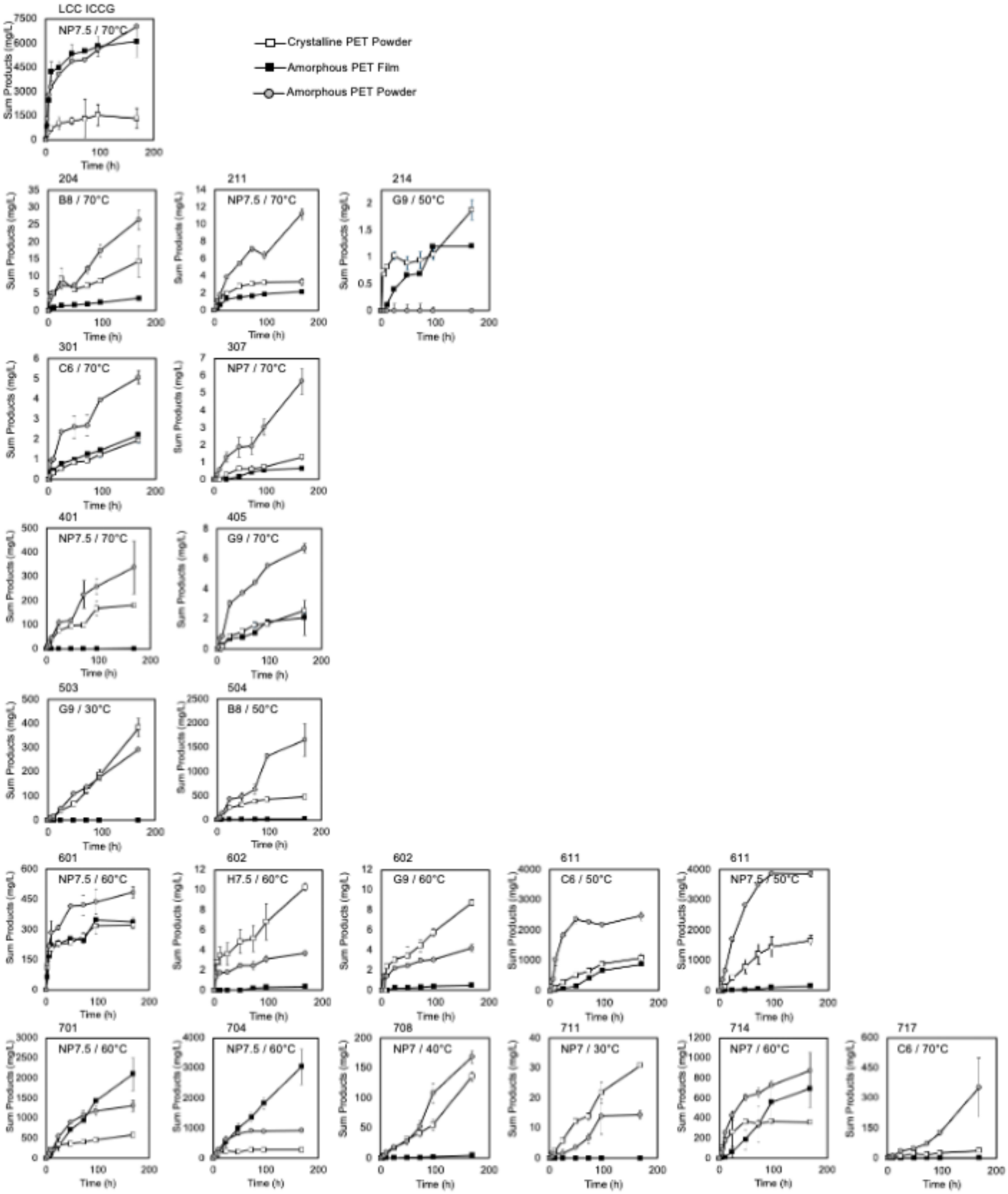

Supplementary Figure 8. Time course plots comparing aromatic product release from amorphous PET film (aFilm, black squares), amorphous PET powder (aPow, grey circles), and crystalline PET powder (cryPow, open squares) over 168 h reaction time. Each plot provides the reaction conditions utilized (citrate at pH 6.0 = C6, NaH_2PO_4 at pH 7.0 = NP7, NaH_2PO_4 at pH 7.5 = NP7.5, HEPES at pH 7.5 = H7.5, bicine at pH 8.0 = B8, and glycine at pH 9.0 = G9), and reaction temperature (30°C, 40°C, 50°C, 60°C, or 70°C). Error bars represent the standard deviation of reactions measured in triplicate (n=3) and are centered on the average of the three measurements. Source data is provided as a Source Data file and can be found within **Source Data Table D7**.

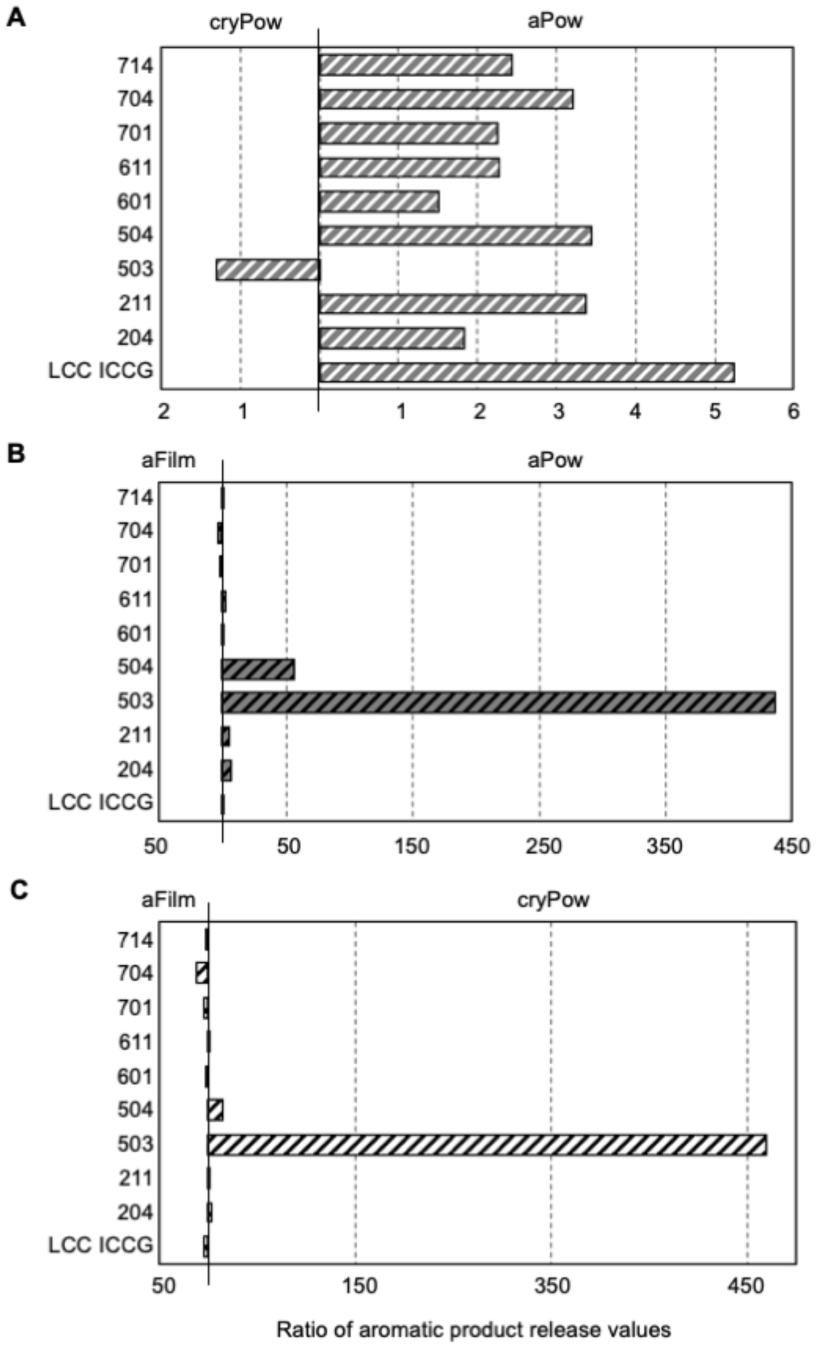

Supplementary Figure 9. Ratios of product release values observed from hydrolysis reactions for each PET substrate morphology pairwise comparison, demonstrating differences in substrate selectivity for each selected enzyme. (**A**) Amorphous powder (aPow, grey) compared to crystalline powder (cryPow, white) is shown in grey and white hatch; (**B**) amorphous powder (aPow, grey) compared to amorphous film (aFilm, black) is shown in grey and black hatch; and (**C**) crystalline powder (cryPow, white) compared to amorphous film (aFilm, black) is shown in white and black hatch. Selective preference for a given substrate morphology is indicated by the direction of the bar for each enzyme. Ratio calculation data is provided in **Supplementary Table 13**. Additional source data is provided as a Source Data file and can be found within **Source Data Table D7**.

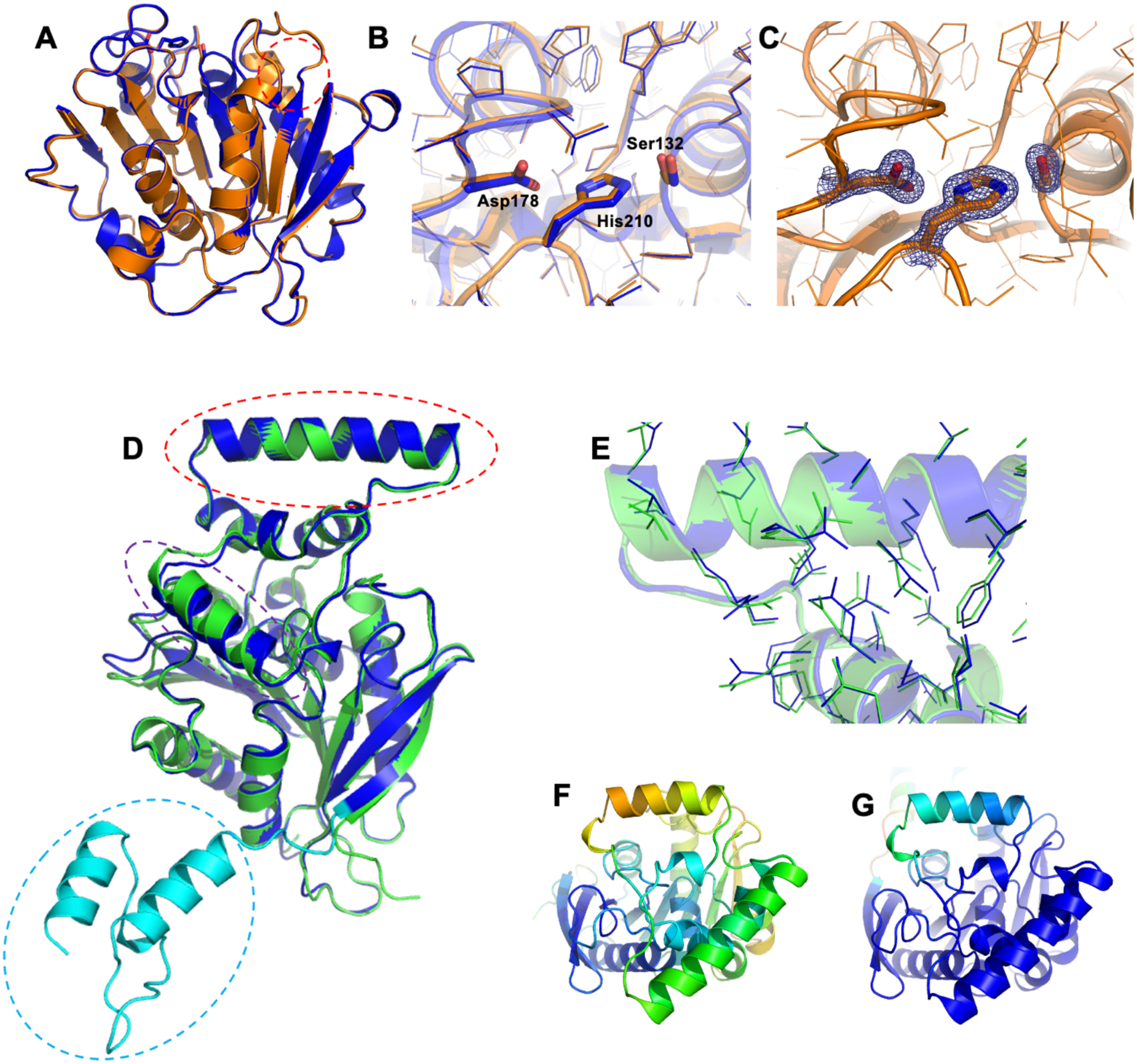

Supplementary Figure 10. Crystallographic and AlphaFold structural comparisons. **(A)** Superposition of crystallographic (orange) and AlphaFold (blue) models of enzyme 611 reveals an almost perfect match to all secondary and tertiary structure elements. The active site region is highlighted with red dashes. **(B)** Closeup of the 611 active site with the catalytic triad shown as sticks. High accuracy alignment can be seen for these and surrounding residues. Representative electron density is shown in **(C)** as a $2F_cF_o$ map contoured at 2 sigma. **(D)** Superposition of crystallographic (green) and AlphaFold (blue) models of enzyme 202 reveals high accuracy. The AlphaFold model predicts an extra domain (cyan) not captured in the crystal structure. **(E)** Close-up of the top helix highlighted in C with red dashes. Alignments are also accurate for both buried and surface exposed residues. **(F)** View of the helical region highlighted with purple dashes in C. The cartoon depicts the crystal structure colored according to B-factor scale, from dark blue (low) to lighter orange and yellow (high). This surface-exposed helix has high B-factors compared to the core of the 202 enzyme and is likely to be dynamic. **(G)** The same region is shown for the AlphaFold model, colored using the pLDDT scale, where dark blue is high confidence predictions and lighter blue and green lower confidence scores. In common with the eight crystallographic-AlphaFold comparisons analyzed in this study, high B-factors often correlate to lower pLDDT scores, making this metric useful for predicting areas of flexibility more generally.

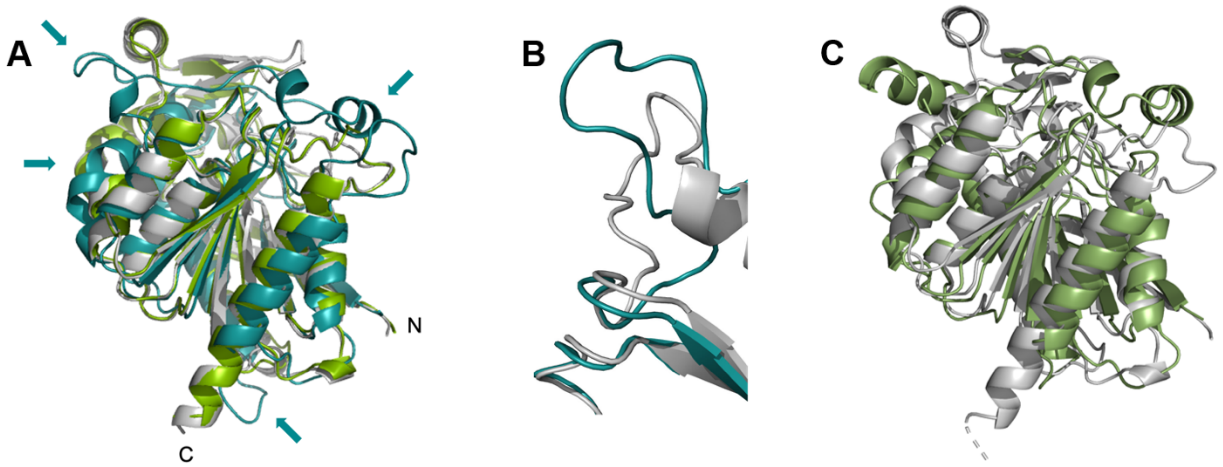

Supplementary Figure 11. Molecular replacement solution of enzyme 306 using an AlphaFold search model. **(A)** Superposition of the solved 306 structure (green) with a model based on PDB ID 6EIC, monoglyceride lipase from *Mycobacterium Tuberculosis* (18.4 % sequence identity) (blue), and the AlphaFold model (grey). Molecular replacement with the *M. Tuberculosis* model was unsuccessful, blue arrows indicate regions of discrepancy. **(B)** Major differences in loop region prediction between the *M. Tuberculosis* model (blue) and AlphaFold models (grey). **(C)** The structure was solved (green) using the more closely modelled AlphaFold model (RMSD = 1.46 Å, 2224 atoms).

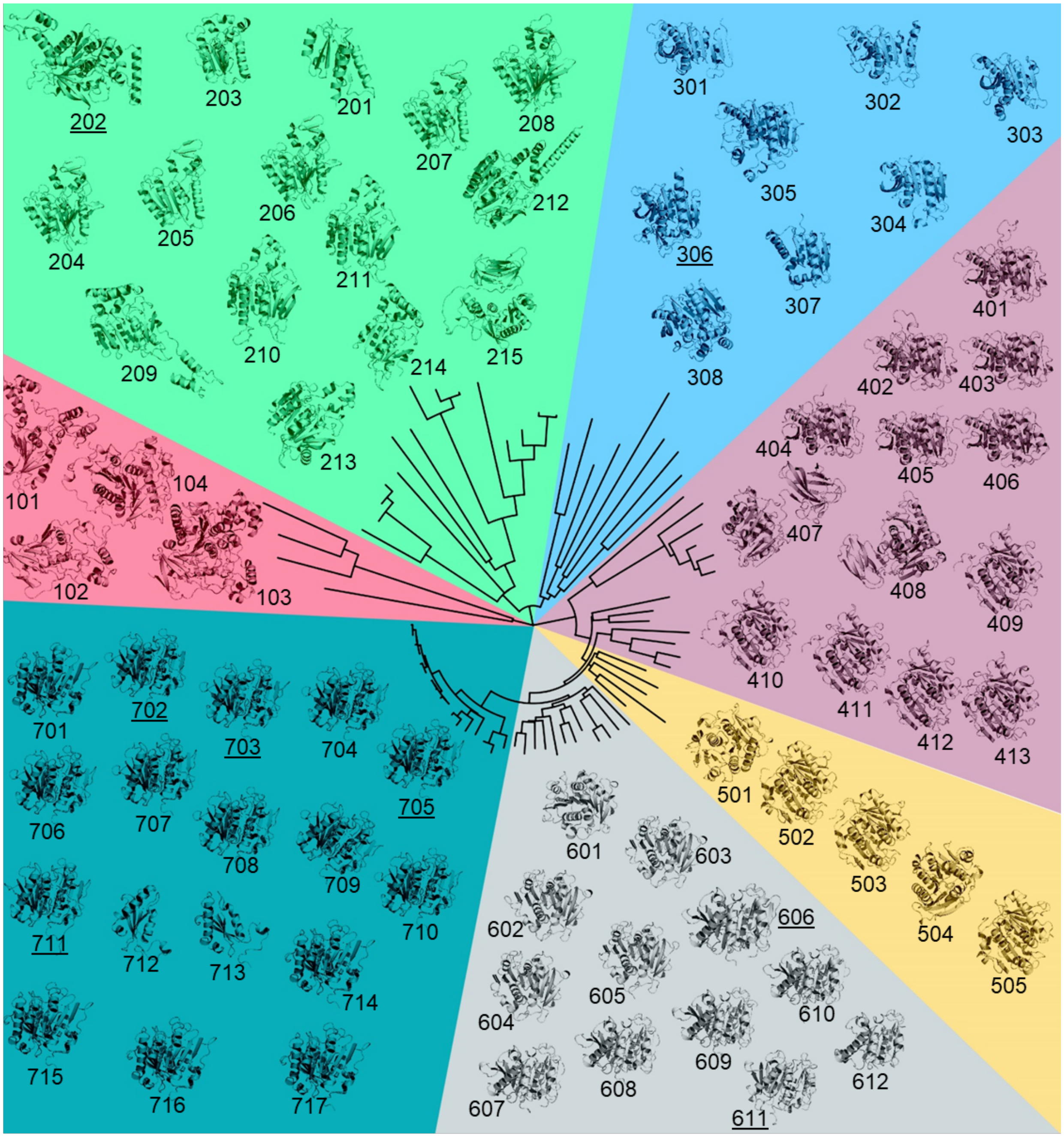

Supplementary Figure 12. Structure gallery. Cartoon models of all 74 structures are shown grouped according to the phylogenetic color scheme in **Figure 1**, main text. The central diagram depicts the minimum-evolution phylogenetic tree of the 74 PET-hydrolase candidates selected by HMM and ML. All models are to scale and incorporate accessory domains where they were observed. Each structure is labelled, and those representatives determined by X-ray crystallography are underlined.

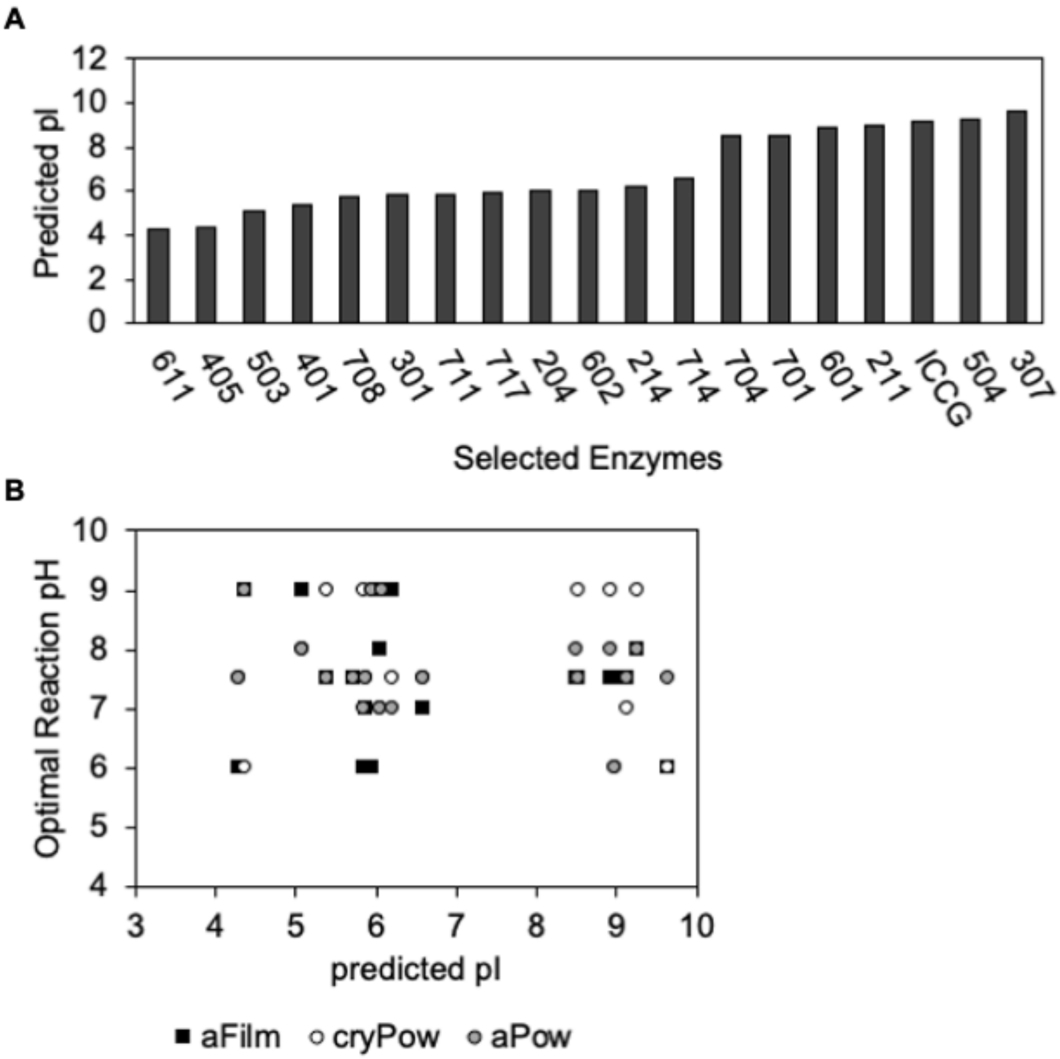

Supplementary Figure 13. Exploring the relationship between enzyme surface charge, represented by the enzyme pI, and the optimal reaction pH. (**A**) Showing the pI of a selection of PET-active enzymes spanning low and high pI from amongst the cohort of 74 candidates, including the ICCG variant of LCC as a control. (**B**) Plot of enzyme pI compared to optimal reaction pH as determined by highest product release observed over 96 h screening reactions using three PET substrates with different morphologies, amorphous film (aFilm, black squares), crystalline powder (cryPow, open circles), and amorphous powder (aPow, grey circles). Theoretical pI values are listed in **Supplementary Table 9.** Source data used to assign the optimal reaction pH can be found within **Source Data Table D6**.

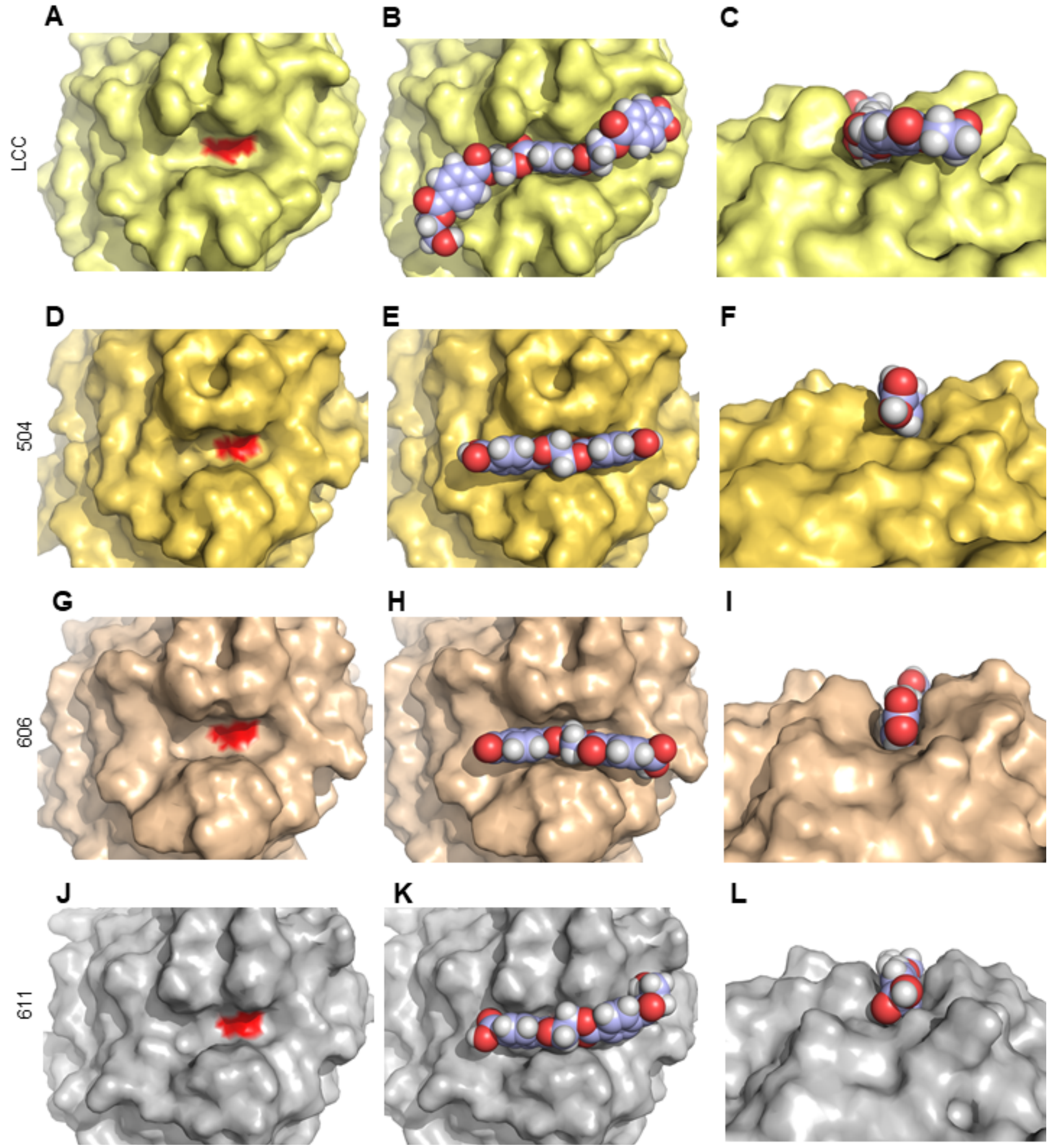

Supplementary Figure 14. Docking analysis of PET comparing binding modes of enzymes LCC, 504, 606, and 611. **(A, D, G & J)** Active site clefts are shown as surface representations with the location of the catalytic serine colored in red. **(B, E, H & K)** Docking of PET chains are shown in the cleft. **(C, F, I & L)** Alternative view of docked PET. These docking poses reveal that the polymer chain sits deeper in the cleft of LCC compared to 504, 606, and 611. LCC also induces a significant twist in the polymer chain as can been seen in the orientation of alternate aromatics in the trimer. In contrast, as shown with a dimer bound to 504, 606, and 611 clefts, adjacent aromatics bind in a relatively straight conformation.

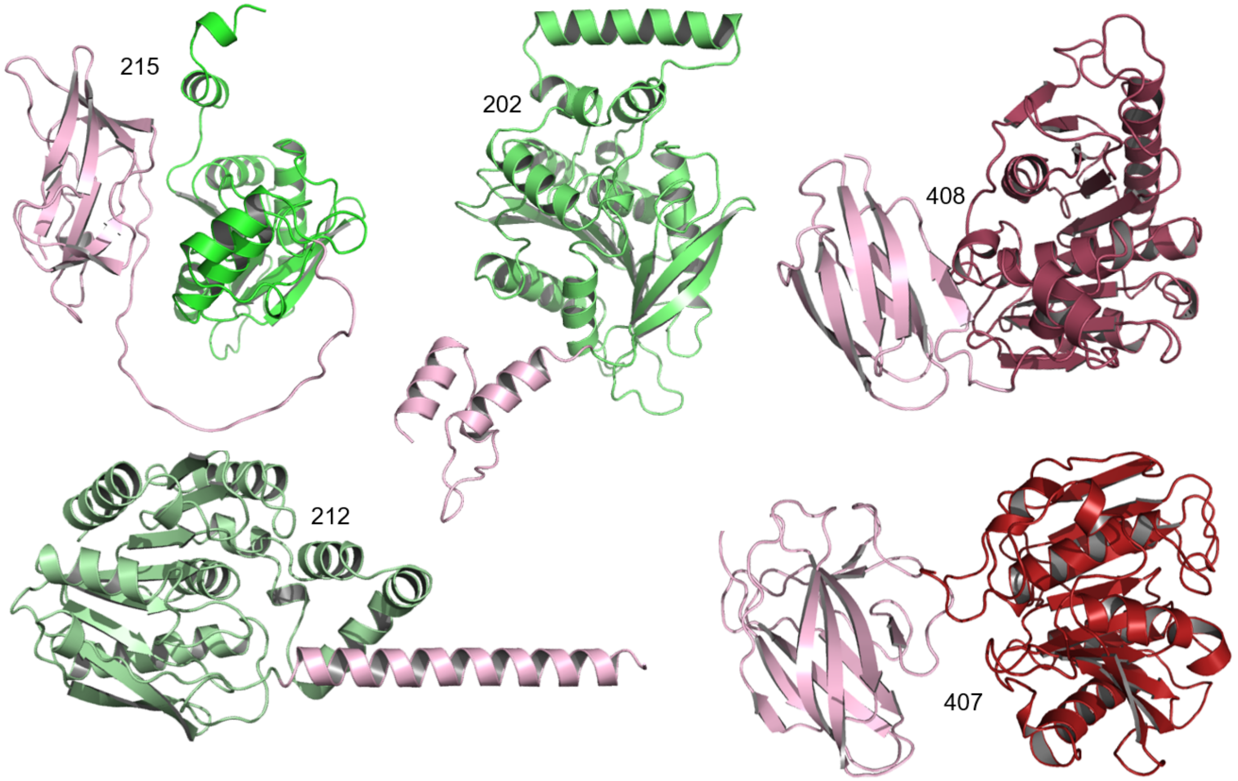

Supplementary Figure 15. Additional appendages. Several additional structures and domains (highlighted in pink) are found in this enzyme cohort predicted as 202 - Peripheral Subunit-Binding Domain, 212 - Transmembrane Helix, 215 – Lipoyl Domain (similar to PDB ID 2DNC), 407 - Family 35 CBM, and 408 - Choice-of-anchor A Domain.

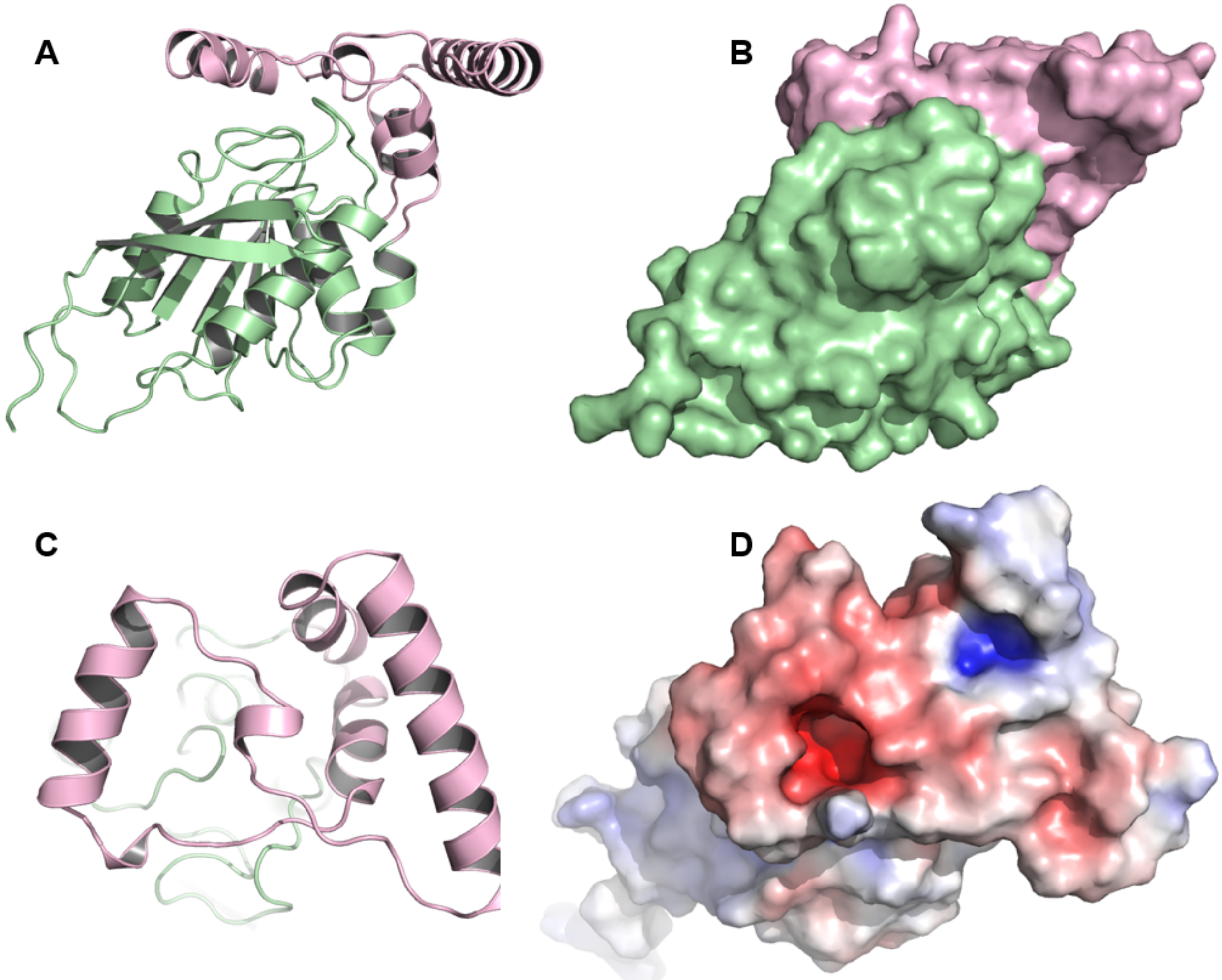

Supplementary Figure 16. Extended helical domain of enzyme 214 creates an unusual flat surface. **(A)** The catalytic domain is shown in green with a multi-helix platform in pink. **(B)** Surface representation in the same orientation as A. **(C)** Top view of the major helices that make up the surface which is approximately 35 x 45 Å. **(D)** Electrostatic surface potential in the same orientation as C reveals strongly polarized areas. The surface is colored with a gradient from red (acidic) at −7 kT/e to blue (basic) at 7 kT/e (where k is Boltzmann's constant, T is temperature, and e is the charge on an electron).

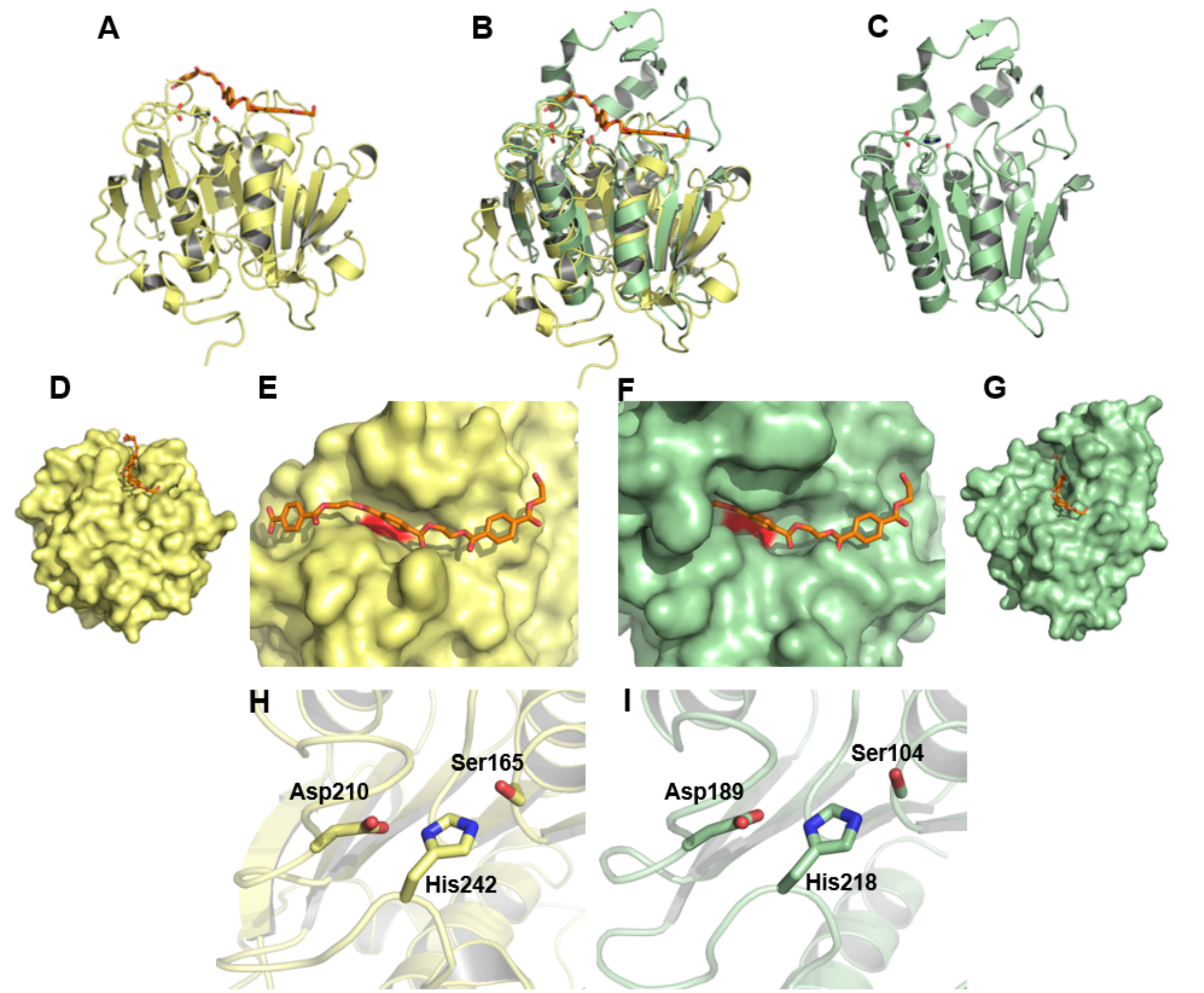

Supplementary Figure 17. The active site of 204. **(A-C)** A superposition of LCC with 204 is shown as cartoon in the middle, with LCC (yellow) and 204 (green) either side in the same orientation. A comparison reveals that the enzyme 204-fold has a more compact core, but with an additional lid domain. A PET trimer docked to LCC is shown in orange. **(D-G)** Surface rendering depicts the active site cleft of LCC (yellow) and 204 (green) with the location of the catalytic Ser shown in red. **(H-I)** Active site residues highlighted as sticks for LCC and 204.

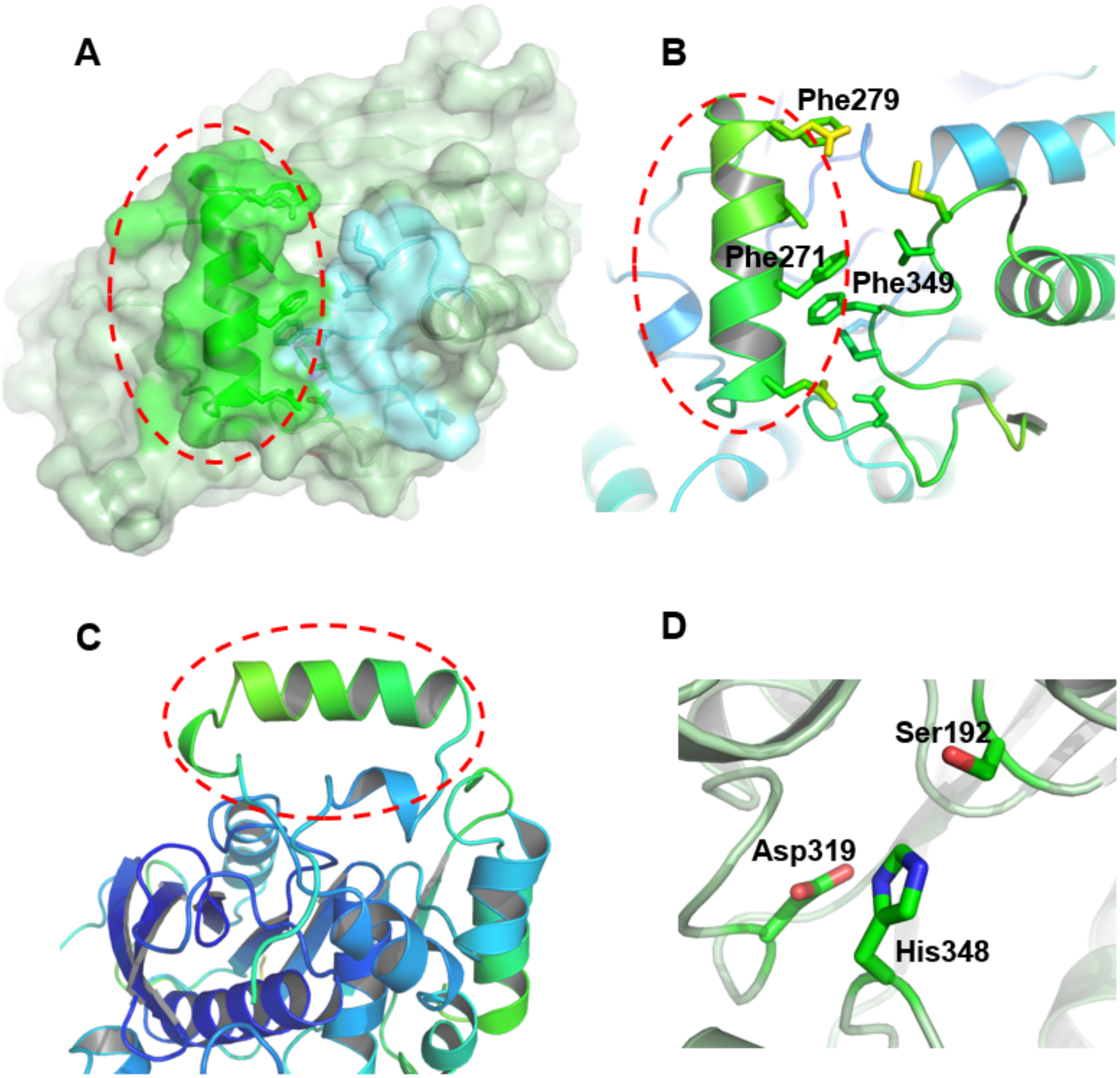

Supplementary Figure 18. The buried active site of enzyme 202. **(A)** A semi-transparent surface representation reveals a helix (green and outlined in red dashes) and loop region (cyan) that come together to close off the active site triad which is located beneath. **(B)** Key residue interactions between the occluding helix and loop include the aromatic residues Phe271, 279 and 349. The model is colored by crystallographic B-factor, indicating that both helix and loop regions are potential dynamic. **(C)** B-factor coloring indicates the relative difference in flexibility between the core (blue, lower B-factors) and the helical cap (green, higher B-factors). **(D)** Despite the substantial differences to canonical PET hydrolases, a familiar active site geometry is retained, constituted by Ser192, Asp 319 and His348.

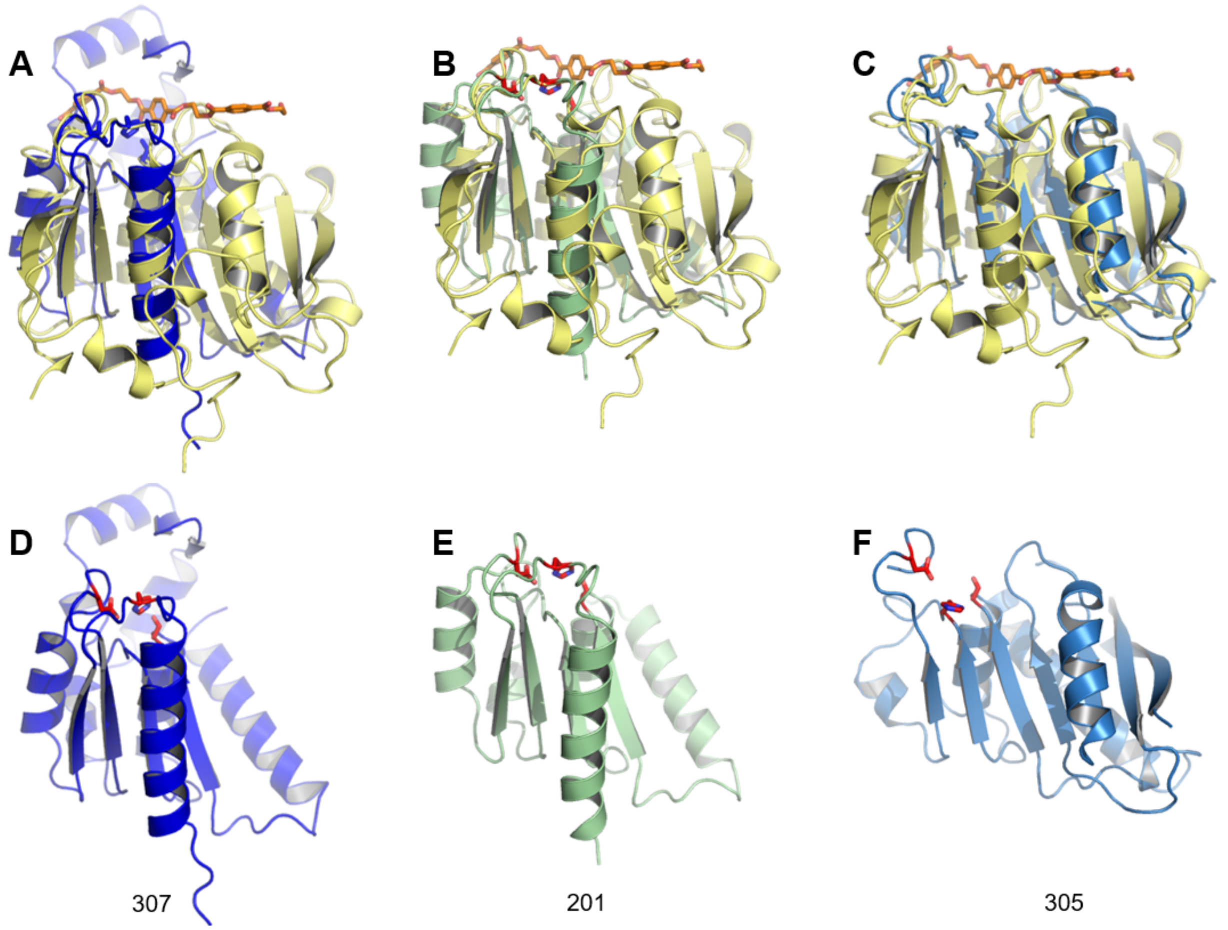

Supplementary Figure 19. Mini-PETase superpositions with LCC. Cartoon representations are shown with LCC in yellow with a docked PET trimer in orange sticks. **(A, B, C)** Superposition of 307 (blue, active on PET), 201 (green, not experimentally validated to be an active PET hydrolase), and 305 (light blue, active on PET) with LCC. **(D, E, F)**. Individual enzymes 307, 201, and 305 shown with predicted active site catalytic residues highlighted in red sticks.

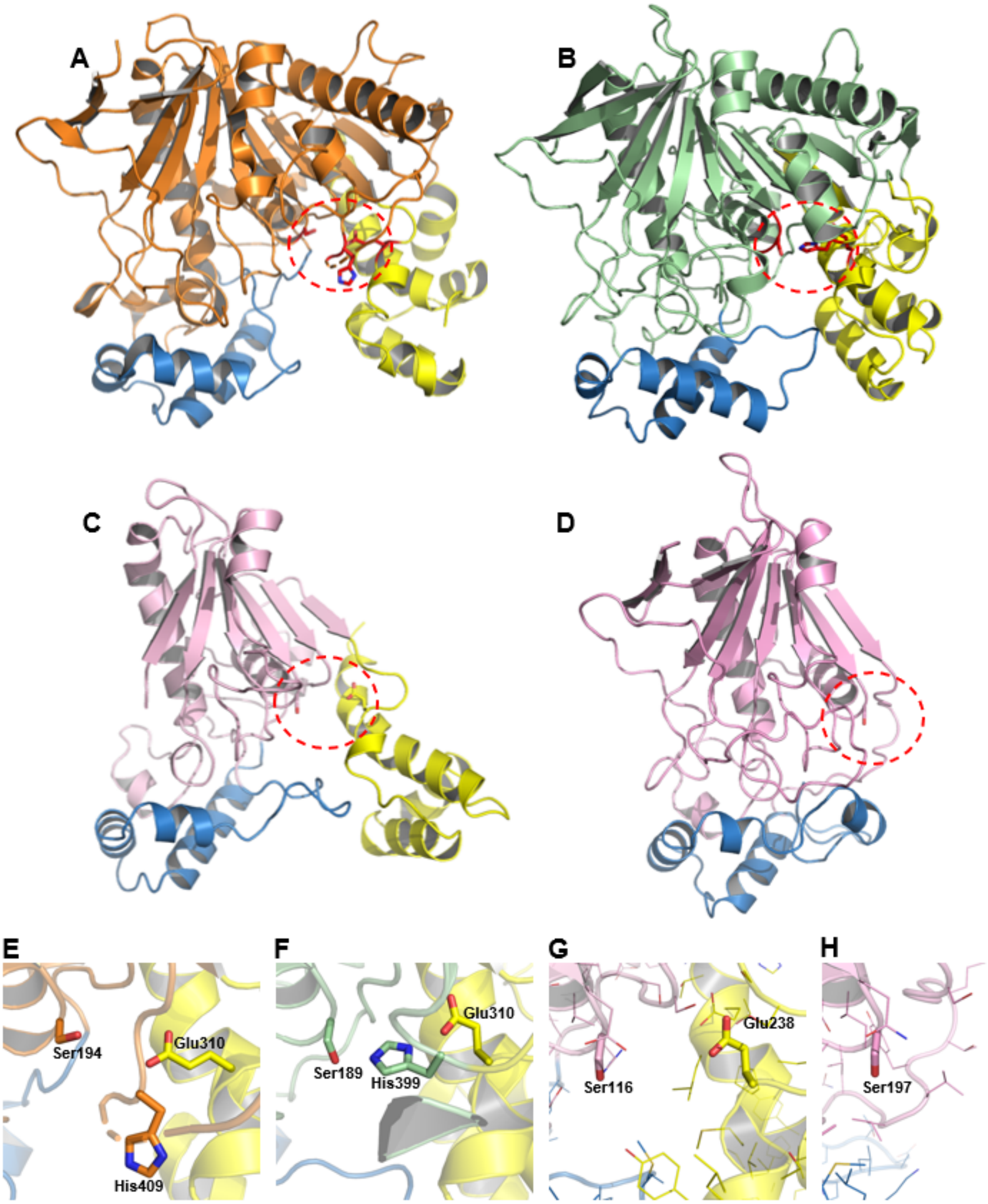

Supplementary Figure 20. Truncated carboxylesterase scaffolds. **(A)** The group 1 enzymes are homologues to carboxylesterases such as the EST55 enzyme from *Geobacillus stearothermophilus* (2), a 3-domain protein with catalytic (orange), regulatory (yellow) and a distinct α/β (blue) components. The active site residues, highlighted in red sticks within the dashed area, are contributed by the catalytic and regulatory domains. **(B)** The *Bacillus subtilis p*-nitrobenzylesterase BsEstB enzyme was previously shown to have activity on PET (3) thus an AlphaFold model was generated for comparison (Enzyme PnbA, Genbank: HM040886.1), revealing the same overall fold as the EST55 enzyme (RMSD of 0.69 Å) with only minor differences, mainly in loop regions. (**C**) Enzyme 101 has a highly truncated catalytic domain (pink) as compared to the EST55 and BsEstB enzymes (orange and green in **A** and **B**, respectively), but does have alternative versions of both the regulatory and α/β domains. (**D**) Enzyme 102 is similar to 101 but lacks the regulatory domain all together. **(E-H)** Comparisons of predicted active site residues in EST55, BsEstB, 101, and 102 enzymes. While EST55 and BsEstB have well defined triads, enzyme 101 lacks an obvious His residue in the vicinity of the catalytic Ser, and 102 lacks both His and Glu residues in the canonical positions. While the Ser position is conserved across this group, further studies will be required to define the mechanism of these highly truncated thermotolerant versions.

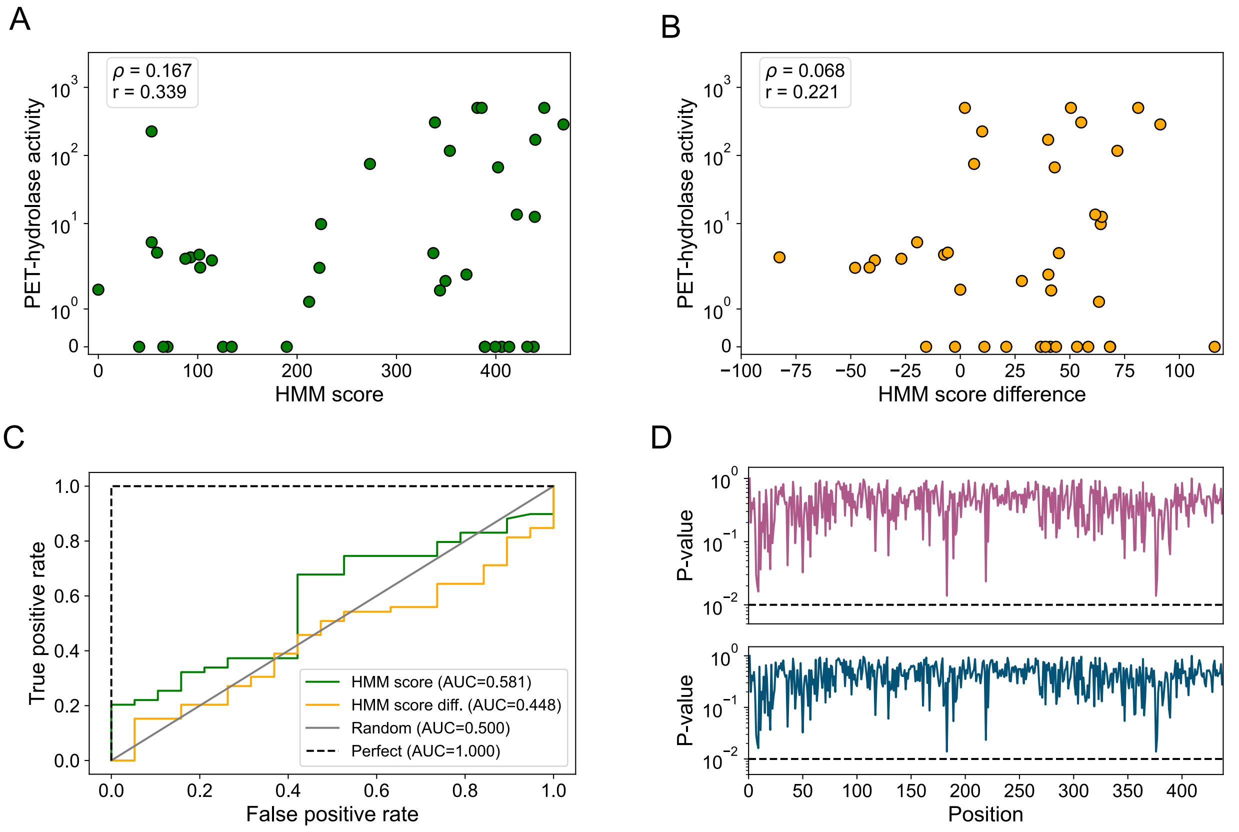

Supplementary Figure 21. Performance of Hidden Markov model (HMM) in predicting PET hydrolase activity (**A**) Relationship between HMM scores and PET-hydrolase activity. HMM scores were derived by searching HMMs constructed from experimentally confirmed PET hydrolases against a dataset of 60 PET hydrolases and 19 inactive homologs. The maximum PET-hydrolase activity observed for the enzymes assayed in this study is shown on the *y*-axis, and was measured as the total amount of aromatic products (BHET, MHET, TPA) released in mg/L (**Figure 2, Source Data Table D3**). The Spearman (ρ) and Pearson (r) correlation coefficient are reported for the data and show a weak relationship between experimental activity and the alignment scores. (**B**) Relationship between the HMM score difference and PET-hydrolase activity. The HMM score difference is derived as the difference between the score obtained by a search against the HMM built on active PETases and the score obtained by a search against the HMM built on inactive homologs. See Supplementary Methods and Materials. (**C**) Receiver operating characteristic (ROC) curve showing the true positive rate and false positive rate obtained from classifying sequences as PETase or non-PETase with varying thresholds of the HMM scores. While a random classifier would be described by the 45° line and an area under the curve (AUC) of 0.5, a hypothetically perfect classifier would be described by the dashed curve and an AUC of 1.0. Classification using HMM scores performs slightly better than random prediction (0.581), and the HMM difference method performs worse than random prediction (AUC=0.448). (**D**) Chi-squared test of independence (two-sided) on amino acid distribution at residue positions in the structure-based alignment (437 positions). The *p*-values for separate Chi-squared tests for each position are shown on the *y*-axes. The first plot above (purple) shows the results of the test for the distribution of 20 canonical amino acids at each position. The plot below (blue) shows the results of the test for the distribution of five amino acid types at each position.

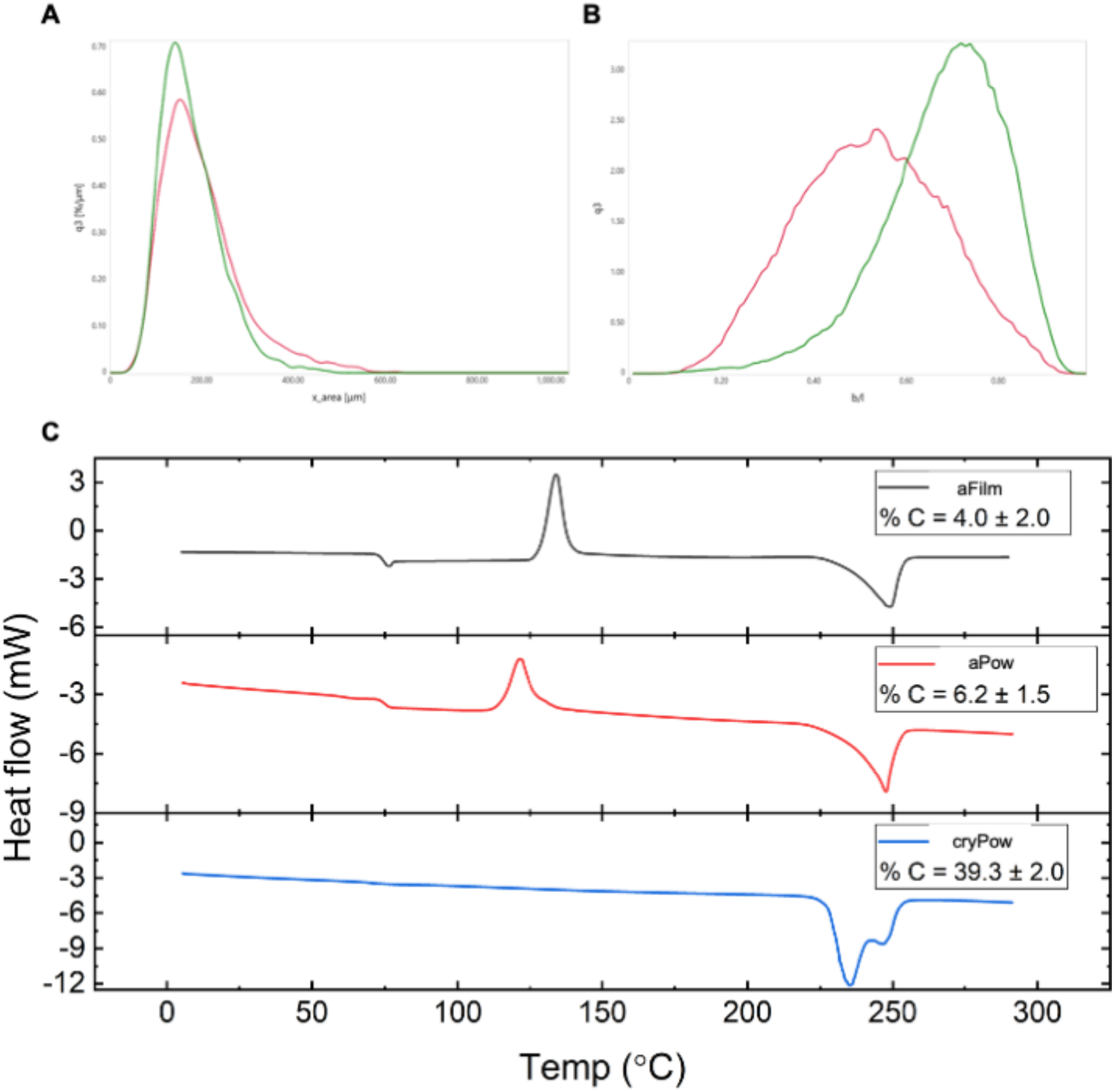

Supplementary Figure 22. Dynamic image analysis of amorphous and crystalline PET powders. (**A**) Population distributions of (**A**) particle cross-sectional area and (**B**) aspect ratio (breadth/length), for the commercial crystalline PET powder (green) and the in-house cryo-milled amorphous PET powder (red). In (A), *x_area* is the radius of a circle within the equivalent cross-sectional area as the particle. The analysis reveals that the two powders have similar cross-sectional area distributions, but the amorphous particles are more elongated. (**C**) DSC analysis of samples evaluated in triplicate for determination of PET crystallinity.

Supplementary Tables

Supplementary Table 1. List of experimentally verified PET hydrolases at the time of this study. The HMM column shows the 17 sequences used in constructing the HMM, which were among the PET hydrolases known at the time of the initial enzyme candidate selection (see Supplementary Materials and Methods). The Candidate Enzyme ID column shows the identifier for sequences that are also contained in our set of 74 putative PET hydrolases.

	**Organism**	**Name**	**Accession**	**HMM**	**Candidate Enzyme ID**	**References**
1	*Ideonella sakaiensis*	IsPETase	GAP38373.1	1		(4-7)
2	*Thermobifida fusca* DSM43793	BTA-1 (TfH, Tfu_0883, Cut2)	WP_011291330.1	2	715	(5, 6, 8-14)
3	Uncultured bacterium	LCC	AEV21261	3	501	(5, 6, 15, 16)
4	*Fusarium solani* pisi	FsC	1CEX_A	4		(5, 6, 10, 17-19)
5	*Thermobifida cellulosilytica* DSM44535	Thc_cut1	ADV92526.1	5		(6, 20)
6	*Thermobifida cellulosilytica* DSM44535	Thc_cut2	ADV92527.1	6	716 (DM)	(6, 20, 21)
7	*Thermobifida fusca* DSM44342	Thf42_cut1	ADV92528.1	7	703	(6, 20)
8	*Thermobifida alba*	Tha_cut1	ADV92525.1	8	707	(6, 22)
9	*Thermobifida halotolerans* DSM44931	Thh_Est	AFA45122.1	9	710	(6, 23)
10	*Sachharomonospora viridus* AHK190	Cut190	BAO42836.1	10		(6, 24)
11	*Humicola insolens*	HiC	4OYY_A	11		(6, 19, 22)
12	*Bacillus subtilis*	BsEstB	ADH43200.1	12		(3, 6)
13	*Thermonospora curvata* DSM43183	Tcur1278	CDN67545.1	13	601	(25)
14	Uncultured bacterium	PET2 (lipIAF5-2)	ACC95208.1	14	401	(26, 27)
15	*Oleispira antartica* RB-8	PET5 (lipA)	CCK74972.1	15		(26)
16	*Vibrio gazogenes*	PET6	WP_021018894.1	16		(26)
17	*Polyangium brachysporum*	PET12 (AAW51_2473)	WP_047194864.1	17		(26)
18	*Thermonospora curvata* DSM43183	Tcur0390	CDN67546.1		602	(25)
19	*Thermobifida fusca* KW3	TfCut1	CBY05529.1		704	(14)
20	*Thermobifida fusca*	BTA2	CAH17554.1		706	(5, 13, 14)
21	*Thermobifida fusca* KW3	TfCut2	CBY05530.1		714	(14, 28-30)

22	*Thermobifida fusca* YX	Tf_0882 (Cut1)	AAZ54920.1		705	(11, 14, 31)
23	*Streptomyces scabiei*	Sub1	QEX94755.1			(32)
24	*Clostridium botulinum* ATCC3502	Cbotu_EstA	AKZ20828.1			(33)
25	Bacterium HR29	BhrPETase	GBD22443.1			(34)
26	*Pseudomonas aestusnigri*	Pe-H	6SBN_A			(35)
27	*Aequorivita* sp. CIP111184	PET27	WP_111881932.1			(36)
28	*Chryseobacterium (Kaistella) jeonii*	PET30	WP_039353427.1			(36)
29	Compost metagenome	PHL1	LT571440			(37)
30	Compost metagenome	PHL2	LT571441			(37)
31	Compost metagenome	PHL3	LT571442			(37)
32	Compost metagenome	PHL4	LT571443			(37)
33	Compost metagenome	PHL5	LT571444			(37)
34	Compost metagenome	PHL6	LT571445			(37)
35	Compost metagenome	PHL7	LT571446			(37)
36	*Thermobifida alba* AHK119	Est119 (Est2)	BAK48590.1		717	(38)

Supplementary Table 2. JGI IMG metagenomes from which putative sequences were derived. These metagenomes comprised a total of 38 million sequences, which were searched against the PETase HMM to derive putative PET hydrolases. The rows that are bolded in the Scaffold Key column highlight metagenomes from which the JGI candidates in our dataset (27 out of 74) were derived.

Scaffold Key	IMG Genome ID	Gold Ecosystem Type	Geographic Location	Sample Temp.	Sample pH
Deep	3300001781	Marine	Cayman Islands, UK	-	-
Ga0063234	3300005209	Thermal springs	Yellowstone National Park, USA	-	-
Ga0063235	3300004269	Thermal springs	Yellowstone National Park, USA	-	
Ga0073359	3300005292	Thermal springs	Yellowstone National Park, USA	-	
Ga0073360	3300005291	Thermal springs	Yellowstone National Park, USA	-	
Ga0073929	3300007070	Thermal springs	British Columbia, Canada	66.4	7.93
Ga0073930	3300007071	Thermal springs	British Columbia, Canada	64.7	7.94
Ga0073931	3300006951	Thermal springs	British Columbia, Canada	85.9	7.08
Ga0073932	3300007072	Thermal springs	British Columbia, Canada	64.7	7.94
Ga0073933	3300006945	Thermal springs	British Columbia, Canada	44.5	8.15
Ga0073934	3300006865	Thermal springs	British Columbia, Canada	33.1	7.16
Ga0074394	3300005396	Thermal springs	Yellowstone National Park, USA	-	-
Ga0079041	3300006857	Thermal springs	Yellowstone National Park, USA	-	-
Ga0079042	3300006181	Thermal springs	Yellowstone National Park, USA	-	-
Ga0079043	3300006179	Thermal springs	Yellowstone National Park, USA	-	-
Ga0079044	3300006855	Thermal springs	Yellowstone National Park, USA	-	-
Ga0079046	3300006859	Thermal springs	Yellowstone National Park, USA	-	-
Ga0079048	3300006858	Thermal springs	Yellowstone National Park, USA	-	-
Ga0105154	3300009598	Thermal springs	Sandy's Spring West, Nevada, USA	86.6	7.03
Ga0105155	3300009591	Thermal springs	Sandy's Spring West, Nevada, USA	86.6	7.03
Ga0105156	3300009596	Thermal springs	Sandy's Spring West, Nevada, USA	86.6	7.03
Ga0105158	3300008019	Thermal springs	Little Hot Creek, California, USA	81.1	6.83
Ga0105159	3300009590	Thermal springs	Little Hot Creek, California, USA	81.1	6.83
Ga0105160	3300009585	Thermal springs	Gongxiaoshe Hot Spring,, China	73.8	7.29
Ga0105161	3300009013	Thermal springs	Gongxiaoshe Hot Spring,, China	71.7	7.46
Ga0105162	3300008000	Thermal springs	Baoshan, Yunnan, China	78.2	6.65
Ga0105163	3300007999	Thermal springs	Baoshan, Yunnan, China	81.6	6.71
Ga0114943	3300009626	Thermal springs	Beatty, Nevada, USA	-	-
Ga0114944	3300009691	Thermal springs	Beatty, Nevada, USA	-	-
Ga0114945	3300009444	Thermal springs	Beatty, Nevada, USA	-	-
Ga0116196	3300010393	Thermal springs	Zodletone Spring, Oklahoma, USA	10.0	7.50
Ga0116197	3300010317	Thermal springs	Zodletone Spring, Oklahoma, USA	10.0	7.50

Ga0116210	3300010288	Thermal springs	Tshipise, South Africa	-	-
Ga0116211	3300010313	Thermal springs	Limpopo, South Africa	-	-
Ga0123519	3300009503	Thermal springs	Yellowstone National Park, USA	-	-
Ga0129299	3300010289	Thermal springs	California, USA	45.6	8.08
Ga0129301	3300010284	Thermal springs	California, USA	45.6	8.08
Ga0129302	3300010291	Thermal springs	California, USA	-	7.48
Ga0137047	3300010484	Thermal springs	British Columbia, Canada	85.9	7.08
Ga0137159	3300010494	Thermal springs	British Columbia, Canada	85.9	7.08
Ga0137169	3300010514	Thermal springs	British Columbia, Canada	85.9	7.08
Ga0137224	3300010600	Thermal springs	British Columbia, Canada	85.9	
Ga0137240	3300010575	Thermal springs	British Columbia, Canada	85.9	7.08
Ga0167615	3300013009	Thermal springs	Yellowstone National Park, USA	68.0	3.00
Ga0167616	3300013008	Thermal springs	Yellowstone National Park, USA	78.0	3.00
Ga0170330	3300013082	Thermal springs	British Columbia, Canada	85.9	7.08
Ga0170563	3300013084	Thermal springs	British Columbia, Canada	85.9	7.08
Ga0170564	3300013085	Thermal springs	British Columbia, Canada	85.9	7.08
**GxsBSedJan11**	3300000865	Thermal springs	Gongxiaoshe pool, Tengchong, China	73.8	7.29
**JGI20127J14776**	3300001382	Thermal springs	Yellowstone National Park, USA	-	-
JGI20128J18817	3300001684	Non-marine saline and alkaline	Yellowstone National Park, USA	-	-
**JGI20132J14458**	3300001339	Thermal springs	Yellowstone National Park, USA	83.0	8.60
JGI24227J36426	3300002555	Thermal springs	Yellowstone National Park, USA	-	-
JGI24228J36427	3300002539	Thermal springs	Yellowstone National Park, USA	-	-
JGI24229J36425	3300002556	Thermal springs	Yellowstone National Park, USA	-	-
JGI24230J36428	3300002540	Thermal springs	Yellowstone National Park, USA	-	-
JGI24231J26847	3300002208	Thermal springs	Yellowstone National Park, USA	-	-
JGI24717J26846	3300002207	Thermal springs	Yellowstone National Park, USA	-	-
JGI24718J22297	3300001986	Thermal springs	Yellowstone National Park, USA	-	-
JGI24721J26819	3300002182	Thermal springs	Yellowstone National Park, USA	-	-
JGI24721J44947	3300005573	Thermal springs	Yellowstone National Park, USA	-	-
JGI26464J51801	3300003604	Thermal springs	Yellowstone National Park, USA	-	-
JGI26465J51735	3300003598	Thermal springs	Yellowstone National Park, USA	-	-
JGI26466J51736	3300003603	Thermal springs	Yellowstone National Park, USA	--	-
JGIcombinedJ22296	3300001987	Thermal springs	Yellowstone National Park, USA	-	-
**JzSedJan11**	3300000866	Thermal springs	Baoshan, Yunnan, China	81.6	6.71
shallow	3300001835	Marine	Cayman Islands, UK	-	-
**YNP11**	2014031007	Thermal springs	Yellowstone National Park, USA	82.0	7.90

YNP15294550	2015219002	Thermal springs	Yellowstone National Park, USA	59.9	8.20
**YNP15490790**	2015219002	Thermal springs	Yellowstone National Park, USA	59.9	8.20
**YNP16**	2016842003	Thermal springs	Yellowstone National Park, USA	36.0	9.10
YNP17	2016842005	Thermal springs	Yellowstone National Park, USA	56.0	5.70
**YNP18**	2016842004	Thermal springs	Yellowstone National Park, USA	76.0	6.40
**YNP20**	2016842008	Thermal springs	Yellowstone National Park, USA	52.0	6.30
YNP3	2014031003	Thermal springs	Yellowstone National Park, USA	80.0	4.00
YNP3A	2016842001	Thermal springs	Yellowstone National Park, USA	80.0	4.00
**YNP6**	2013515000	Thermal springs	Yellowstone National Park, USA	50.0	-
YNP7	2014031006	Thermal springs	Yellowstone National Park, USA	52.9	6.00
**YNPsite05**	2022920003	Thermal springs	Yellowstone National Park, USA	57.6	6.20
**YNPsite06**	2022920004	Thermal springs	Yellowstone National Park, USA	50.0	-
YNPsite07	2022920013	Thermal springs	Yellowstone National Park, USA	52.9	6.00
YNPsite11	2022920012	Thermal springs	Yellowstone National Park, USA	82.0	7.90
YNPsite15	2022920016	Thermal springs	Yellowstone National Park, USA	59.9	8.20
**YNPsite16**	2022920018	Thermal springs	Yellowstone National Park, USA	36.0	9.10
YNPsite17	2022920021	Thermal springs	Yellowstone National Park, USA	56.0	5.70
YNPsite18	2022920019	Thermal springs	Yellowstone National Park, USA	76.0	6.40
YNPsite20	2022920020	Thermal springs	Yellowstone National Park, USA	52.0	6.20

Supplementary Table 3. Organism and sequence dataset used in machine learning prediction of thermophilicity (ThermoProt). Details of the sequence selection and machine learning are in the Supplementary Materials and Methods.

	Organism	Group	Growth/Optimum Temp (ºC)		Number of Proteins	Class	Used in
			NCBI	BacDive			
1	*Psychroflexus torquis*	Bacteria	0-15	4	9,953	Psychrophilic	Validation
2	*Moritella sp.*	Bacteria	5-8	5	31,433	Psychrophilic	Validation
3	*Colwellia psychrerythraea*	Bacteria	8	10	31,845	Psychrophilic	Validation
4	*Rhodonellum psychrophilum*	Bacteria	5	5 - 28	5,035	Psychrophilic	Testing
5	*Vibrio mediterranei*	Bacteria	26	25-28	48,521	Mesophilic	Validation
6	*Parvimonas micra*	Bacteria	37	37	12,460	Mesophilic	Validation
7	*Aeromonas enteropelogenes*	Bacteria	36	30	27,934	Mesophilic	Validation
8	*Methylobacillus flagellatus*	Bacteria	30 - 42	30	5,743	Mesophilic	Testing
9	*Thermogemmatispora onikobensis*	Bacteria	60-65	60-65	4,255	Thermophilic	Validation
10	*Thermovenabulum gondwanense*	Bacteria	65	65	4,424	Thermophilic	Validation
11	*Acidianus brierleyi*	Archaea	70	70	10,479	Thermophilic	Validation
12	*Metallosphaera sedula*	Archaea	70	65	18,352	Thermophilic	Validation
13	*Thermomicrobium roseum*	Bacteria	70	70	5,641	Thermophilic	Validation
14	*Thermobifida fusca*	Bacteria	50-55	45-60	19,415	Thermophilic	Validation
15	*Ardenticatena maritima*	Bacteria	60	62 - 65	8,881	Thermophilic	Testing
16	*Methanocaldococcus vulcanius*	Archaea	80	80	3,446	Hyperthermophiilic	Validation
17	*Thermococcus sp.*	Archaea	85	80	39,447	Hyperthermophiilic	Validation
18	*Vulcanisaeta distributa*	Archaea	85-90	90	4,921	Hyperthermophiilic	Validation
19	*Geoglobus ahangari*	Archaea	88	85	3,958	Hyperthermophiilic	Validation
20	*Thermococcus guaymasensis*	Archaea	88	88	4,121	Hyperthermophiilic	Validation
21	*Aeropyrum pernix*	Archaea	90-95	90-95	18,861	Hyperthermophiilic	Validation
22	*Pyrococcus kukulkanii*	Archaea	105	105	4,061	Hyperthermophiilic	Validation
23	*Pyrolobus fumarii*	Archaea	106	103	3,875	Hyperthermophiilic	Validation
24	*Thermotoga petrophila*	Bacteria	80	80	2,640	Hyperthermophilic	Testing

Supplementary Table 4. Sequence features used in machine-learning prediction of thermophilicity (ThermoProt). The last column shows the Spearman correlation coefficient between the computed features and the thermostability class (psychrophilic=1, mesophilic=2, thermophilic=3, hyperthermophilic=4).

	Feature	Description	Correlation (ρ)
1	A composition	n_A/n_{total}	0.013
2	C composition	n_C/n_{total}	-0.081
3	D composition	n_D/n_{total}	-0.158
4	E composition	n_E/n_{total}	0.216
5	F composition	n_F/n_{total}	-0.137
6	G composition	n_G/n_{total}	0.201
7	H composition	n_H/n_{total}	-0.162
8	I composition	n_I/n_{total}	0.010
9	K composition	n_K/n_{total}	-0.085
10	L composition	n_L/n_{total}	0.071
11	M composition	n_M/n_{total}	-0.039
12	N composition	n_N/n_{total}	-0.318
13	P composition	n_P/n_{total}	0.196
14	Q composition	n_Q/n_{total}	-0.427
15	R composition	n_R/n_{total}	0.358
16	S composition	n_S/n_{total}	-0.258
17	T composition	n_T/n_{total}	-0.182
18	V composition	n_V/n_{total}	0.316
19	W composition	n_W/n_{total}	0.024
20	Y composition	n_Y/n_{total}	0.088
21	AA 0-gap dipeptide composition	$n_{AA}/(n_{total}-1)$	-0.033
22	RE 0-gap dipeptide composition	$n_{RE}/(n_{total}-1)$	0.274
23	RR 0-gap dipeptide composition	$n_{RR}/(n_{total}-1)$	0.272
24	EQ 0-gap dipeptide composition	$n_{EQ}/(n_{total}-1)$	-0.230
25	QA 0-gap dipeptide composition	$n_{QA}/(n_{total}-1)$	-0.198
26	KQ 0-gap dipeptide composition	$n_{KQ}/(n_{total}-1)$	-0.240
27	R*R 1-gap dipeptide composition	$n_{R*R}/(n_{total}-2)$	0.187
28	A**R 2-gap dipeptide composition	$n_{A**R}/(n_{total}-3)$	0.095
29	L**Q 2-gap dipeptide composition	$n_{L**Q}/(n_{total}-3)$	-0.302
30	R**R 2-gap dipeptide composition	$n_{R**R}/(n_{total}-3)$	0.259
31	Acidic residue composition	$\sum n_x/n_{total}$ for x in [D,E]	0.081
32	Basic residue composition	$\sum n_x/n_{total}$ for x in [K,R,H]	0.141
33	Non-polar residue composition	$\sum n_x/n_{total}$ for x in [A,G,I,L,M,F,P,W,V]	0.231
34	Cyclic residue composition	$\sum n_x/n_{total}$ for x in [F,Y,W,P,H]	0.021
35	Aliphatic residue composition	$\sum n_x/n_{total}$ for x in [A,G,I,L,V]	0.248
36	Aromatic residue composition	$\sum n_x/n_{total}$	-0.094

		for x in [H,F,W,Y]	
37	Charged residue composition	$\sum n_x/n_{total}$ for x in [D,E,K,R,H]	0.131
38	Basic/acidic ratio	$Basic/Acidic$	0.019
39	Non-polar/polar ratio	$Non\text{–}polar/(1 - Non\text{–}polar)$	0.192
40	Cyclic/acyclic ratio	$Cyclic/(1 - Cyclic)$	0.023
41	Charged/non-charged ratio	$Charged/(1 - Charged)$	0.152
42	EFMR composition	$\sum n_x/n_{total}$ for x in [E, F, M, R]	0.310
43	(E+K)/(Q+H)	$\frac{n_E + n_K}{n_Q + n_H}$	0.290
44	Charged vs. polar	$\sum n_x/n_{total} - \sum n_y/n_{total}$ for x in [D,E,K,R], for y in [N,Q,S,T]	0.396
45	IVYWREL composition	$\sum n_x/n_{total}$ for x in [I,V,Y,W,R,E,L]	0.525
46	Tiny residues composition	$\sum n_x/n_{total}$ for x in [A,G,P,S]	0.062
47	Small residues (TD) composition	$\sum n_x/n_{total}$ for x in [T,D]	-0.246
48	Average maximum ASA	$\sum (n_x/n_{total} \times A_x)$ for x in all 20 amino acids, A_x is the maximum solvent accessible surface area of amino acid, x.	0.023
49	Molecular weight (kDa)	$\sum (n_x \times W_x)$ for x in all 20 amino acids, W_x is the molecular weight of amino acid, x.	-0.063
50	Heat capacity	$\sum (n_x/n_{total} \times c_x)$ for x in all 20 amino acids, c_x is heat capacity of amino acid, x.	-0.178

Supplementary Table 5. Accuracy (%) of binary classifiers in discriminating psychrophilic from mesophilic proteins (PM), mesophilic from thermophilic proteins (MT), thermophilic from hyperthermophilic proteins (TH), and mesophilic from thermophilic and hyperthermophilic proteins (MTH). Errors represent one standard deviation over fivefold cross-validation on the dataset of 32,000 proteins.

	PM	MT	TH	MTH
Logistic regression	71.0 ± 0.9	80.5 ± 0.9	76.6 ± 0.2	82.4 ± 0.8
KNN	69.6 ± 0.8	83.3 ± 0.4	81.0 ± 0.9	83.6 ± 0.3
Naïve Bayes	68.0 ± 0.9	73.9 ± 0.7	70.8 ± 0.6	77.0 ± 1.2
Random forests	73.0 ± 0.6	84.5 ± 0.5	82.9 ± 0.5	85.2 ± 0.5
SVM (RBF kernel)	74.0 ± 0.5	85.5 ± 0.4	83.3 ± 0.6	86.6 ± 0.8

Supplementary Table 6. Validation performance of the SVM (ThermoProt) measured over fivefold cross-validation.

	PM	MT	TH	MTH
Accuracy	74.0 ± 0.5	85.5 ± 0.4	83.3 ± 0.6	86.6 ± 0.8
True positive rate	76.2 ± 0.7	86.1 ± 0.5	80.5 ± 0.5	87.0 ± 1.2
True negative rate	72.1 ± 0.5	85.0 ± 0.5	86.6 ± 1.5	86.3 ± 0.9
Matthew's Correlation Coefficient (MCC)	0.48 ± 0.01	0.71 ± 0.01	0.67 ± 0.01	0.73 ± 0.02

Supplementary Table 7. Accuracy of ThermoProt on the separate testing dataset of 22,299 proteins.

Organism	Accuracy			
	PM	MT	TH	MTH
R. psychrophilum (P)	75.0	-	-	-
M. flagellatus (M)	87.1	80.1	-	82.5
A. maritima (T)	-	80.2	85.8	77.2
T. petrophila (H)	-	-	86.1	86.9

Supplementary Table 8. Maximum sequence identity between 37 experimentally confirmed PET hydrolases presented in this study (**Supplementary Table 10**) as well as enzyme 709, which was previously reported as an active PET hydrolase (Thh_Est) but did not show activity in our assay conditions, and previously reported PET hydrolases (**Supplementary Table 1**). Sequence identities were determined from a multiple sequence alignment and the sequences in bold (14 sequences) are identical to previously reported PET hydrolases.

	**Enzyme ID (this study)**	**Most similar previously reported enzyme**	**Maximum sequence identity (%)**
1	101	BsEstB	42.2
2	102	BsEstB	39.2
3	202	PHL7	21.6
4	204	Pe-H	22.8
5	211	PET5	20.5
6	214	BTA2	20.9
7	301	Pe-H	30.0
8	305	Tcur1278	20.3
9	307	Pe-H	16.9
10	**401**	**PET2**	**100.0**
11	403	PET5	65.0
12	405	PHL2	61.5
13	406	PET12	53.0
14	407	PHL2	54.6
15	409	PET30	31.7
16	412	Thc_cut2	31.3
17	**501**	**LCC**	**100.0**
18	503	Tcur1278	56.6
19	504	Tcur1278	53.5
20	**601**	**Tcur1278**	**100.0**
21	**602**	**Tcur0390**	**100.0**
22	606	PHL2	71.2
23	607-nSP	PHL7	68.0
24	611	PHL7	66.0
25	701	Thf42_cut1	99.6
26	702	BTA-1	98.9
27	**703**	**Thf42_cut1**	**100.0**
28	**704**	**TfCut1**	**100.0**
29	**705**	**Tf_0882**	**100.0**
30	**706**	**BTA2**	**100.0**
31	**707**	**Tha_cut1**	**100.0**
32	708	Est119	95.0
33	**709**	**Thh_Est**	**100.0**
34	711	Est119	80.4
35	**714**	**TfCut2**	**100.0**
36	**715**	**BTA-1**	**100.0**
37	**716**	**Thc_cut2**	**98.9**
38	**717**	**Est119**	**100.0**

Supplementary Table 9. Annotated list of the 74 candidate enzymes. The HMM score column shows the alignment scores obtained by searching the HMM built with 17 experimentally confirmed PETases against the NCBI and JGI databases. Sequences in groups 1 to 3 were retrieved from JGI IMG and the accession column shows the scaffold ID mapping the sequence to the corresponding metagenome (see **Supplementary Table 2**). Sequences in groups 4 to 7 were retrieved from NCBI and the accession column shows the GenBank accession number. OGT indicates the optimal growth temperature, or the specific environment temperature, if available. Otherwise (N/A), the sequence was selected as a thermophilic enzyme by prediction with ThermoProt. Enzymes selected for additional characterization based on the predicted pI are highlighted in light grey.

	Group	Enzyme ID	Accession/ID	Organism	HMM score	HMM Coverage	Sequence Length	OGT (ºC)	Theoretical pI	Predicted molecular weight (w/o His tag)
1		101	YNPsite06_CeleraDRAFT_263770	Environmental sample	34.6	5-273	301	**N/A**	7.10	32.2
2	1	102	YNP6_02150	Environmental sample	35.1	20-228	326	**N/A**	5.42	31.0
3		103	GxsBSedJan11_10003667	Environmental sample	35.3	89-393	518	**73.8**	6.49	55.0
4		104	YNP16_304900	Environmental sample	30.8	86-213	366	**N/A**	4.97	41.0
5		201	YNP15490790	Environmental sample	28.9	19-115	137	**59.9**	5.08	15.6
6		202	YNPsite05_CeleraDRAFT_401410	Environmental sample	30.3	85-223	380	**57.6**	6.03	41.5
7		203	YNP16_189140	Environmental sample	27.5	82-189	197	**N/A**	9.47	21.6
8		204	YNP18_240440	Environmental sample	40.5	45-241	241	**N/A**	6.07	27.0
9		205	JzSedJan11_10146151	Environmental sample	45.4	3-176	195	**81.6**	5.99	22.2
10		206	JGI20127J14776_10147151	Environmental sample	37.8	46-241	241	**80.0**	6.33	27.0
11		207	YNPsite18_CeleraDRAFT_262380	Environmental sample	45.8	20-213	214	**N/A**	6.91	24.0
12	2	208	JzSedJan11_10131225	Environmental sample	37.6	20-248	256	**81.6**	6.51	29.0
13		209	YNPsite20_CeleraDRAFT_325860	Environmental sample	29.8	84-222	345	**N/A**	5.77	37.4
14		210	JzSedJan11_10073025	Environmental sample	28.3	33-258	275	**81.6**	8.98	31.5
15		211	JzSedJan11_10004914	Environmental sample	27.5	18-244	263	**81.6**	8.98	30.0
16		212	JGI20127J14776_100005829	Environmental sample	31.7	100-266	300	**80.0**	9.03	34.0
17		213	JzSedJan11_10131031	Environmental sample	30.9	28-253	274	**81.6**	6.73	31.5
18		214	YNPsite06_CeleraDRAFT_160970	Environmental sample	28.0	23-150	239	**N/A**	6.22	26.5
19		215	GxsBSedJan11_10061611	Environmental sample	28.4	180-269	321	**73.8**	5.59	34.0
20		301	YNPsite06_CeleraDRAFT_367810	Environmental sample	54.1	26-194	238	**N/A**	5.86	22.5
21		302	YNPsite16_CeleraDRAFT_71360	Environmental sample	30.7	54-180	218	**N/A**	7.06	23.5
22		303	YNPsite16_CeleraDRAFT_248770	Environmental sample	54.4	59-265	338	**N/A**	6.00	37.0
23	3	304	YNP11_222720	Environmental sample	38.9	20-180	224	**N/A**	9.1	26.0
24		305	GxsBSedJan11_10251181	Environmental sample	27.8	29-147	231	**73.8**	6.5	25.5
25		306	GxsBSedJan11_10009658	Environmental sample	27.2	29-246	283	**73.8**	6.01	32.1
26		307	JGI20132J14458_10325381	Environmental sample	30.7	11-173	177	**80**	9.66	21.1
27		308	JzSedJan11_10355852	Environmental sample	27.7	13-247	283	**81.6**	8.35	33.0
28		401	ACC95208.1	uncultured bacterium	360.0	21-308	308	N/A	5.40	30.0
29		402	WP_101893885.1	*Ketobacter alkanivorans*	360.7	23-314	314	N/A	5.57	32.0
30		403	RLU00646.1	*Ketobacter sp.*	353.9	21-312	312	N/A	4.52	31.0
31	4	404	WP_012854926.1	*Thermomonospora curvata*	329.5	26-294	295	50.0	5.83	29.0
32		405	WP_082414832.1	*Actinobacteria bacterium*	318.5	12-299	302	N/A	4.37	29.0
33		406	ODU60407.1	*Comamonadaceae bacterium*	298.2	22-305	305	N/A	8.30	31.5
34		407	WP_117215036.1	*Micromonosporaceae bacterium*	247.8	20-298	434	N/A	7.68	41.5

35		408	RCL73670.1	*Flavobacteriales bacterium*	137.9	57-279	366	N/A	4.29	40.0
36		409	RLT92980.1	*Ketobacter sp.*	122.8	40-254	269	N/A	7.75	29.0
37		410	RLT88027.1	*Alcanivoracaceae bacterium*	111.0	40-290	311	N/A	6.41	30.2
38		411	RLU03930.1	*Ketobacter sp.*	104.9	40-269	287	N/A	4.75	29.5
39		412	WP_101893509.1	*Ketobacter alkanivorans*	114.5	39-265	283	N/A	8.49	30.2
40		413	WP_115481747.1	*Robinsoniella sp.*	104.2	38-299	315	N/A	9.43	34.0
41		501	4EB0_A	uncultured bacterium	355.1	22-257	258	N/A	9.32	28.0
42		502	PKO68961.1	*Betaproteobacteria bacterium*	335.5	24-289	289	N/A	9.49	28.0
43	5	503	EGD44994.1	*Nocardioidaceae bacterium*	296.7	21-293	294	N/A	5.10	28.0
44		504	WP_062195544.1	*Caldimonas taiwanensis*+D57	314.9	11-292	292	55.0	9.26	29.5
45		505	OGP67040.1	*Deltaproteobacteria bacterium*	228.9	7-282	282	N/A	9.26	27.5
46		601	WP_012851645.1	*Thermomonospora curvata*	383.2	15-288	289	50.0	8.93	29.0
47		602	WP_012850775.1	*Thermomonospora curvata*	377.4	24-291	292	50.0	6.08	29.0
48		603	WP_119925005.1	*Streptosporangiaceae bacterium*	377.7	35-303	305	N/A	5.82	28.5
49		604	WP_113973098.1	*Micromonospora sp.*	364.5	30-295	296	N/A	6.08	27.5
50		605	WP_106963453.1	*Actinomycetia*	369.4	24-285	287	N/A	6.42	29.0
51	6	606	WP_078759821.1	*Marinactinospora thermotolerans*	365.7	39-309	311	55.0	4.43	29.0
52		607	WP_107095481.1	*Actinobacteria bacterium*	378.2	45-308	310	N/A	5.47	28.0
53		608	WP_119951510.1	*Frankiales bacterium*	355.0	47-313	313	N/A	6.30	28.0
54		609	WP_125778035.1	*Promicromonosporaceae bacterium*	369.3	39-306	307	N/A	5.39	28.5
55		610	WP_125089638.1	*Saccharopolyspora sp.*	347.8	33-293	293	45.0	4.48	29.0
56		611	WP_093412886.1	*Saccharopolyspora flava*	353.5	32-293	293	45.0	4.31	28.5
57		612	OWY58880.1	*cyanobacterium* TDX16	214.0	1-175	175	N/A	6.4	19.0
58		701	WP_104613137.1	*Thermobifida fusca*	435.8	28-301	301	50.0	8.52	29.0
59		702	ADM47605.1	*Thermobifida fusca*	433.5	2-262	262	50.0	6.3	29.0
60		703	ADV92528.1	*Thermobifida fusca*	432.0	2-262	262	50.0	7.02	28.5
61		704	CBY05529.1	*Thermobifida fusca*	430.5	46-319	319	50.0	8.50	29.0
62		705	AAZ54920.1	*Thermobifida fusca*	426.2	48-319	319	50.0	6.97	29.0
63		706	CAH17554.1	*Thermobifida fusca*	425.6	30-301	301	50.0	8.5	29.0
64		707	ADV92525.1	*Thermobifida alba*	424.8	2-262	262	50.0	6.59	28.5
65		708	BAI99230.2	*Thermobifida alba*	414.4	23-287	296	50.0	5.74	29.0
66	7	709	WP_068752972.1	*Thermobifida cellulosilytica*	411.8	25-300	300	50.0	6.30	29.0
67		710	AFA45122.1	*Thermobifida halotolerans*	405.8	2-262	262	50.0	5.24	29.0
68		711	WP_083947829.1	*Thermobifida cellulosilytica*	403.9	11-284	284	50.0	5.87	29.0
69		712	RII04304.1	*Thermobifida halotolerans*	182.2	28-162	162	50.0	4.47	13.0
70		713	RII04310.1	*Thermobifida halotolerans*	180.9	35-168	168	50.0	4.67	13.5
71		714	CDN67547.1	*Thermobifida fusca*	437.5	1-261	262	50.0	6.59	29.0
72		715	ALF04778.1	*Thermobifida fusca*	437.2	1-261	269	50.0	6.30	28.5
73		716	5LUK_A	*Thermobifida cellulosilytica*	426.6	2-262	265	50.0	6.21	29.0
74		717	3VIS_A	*Thermobifida alba*	408.1	31-306	306	50.0	5.96	29.0

Supplementary Table 10. ESTHER family classification and EC number prediction of candidate sequences with experimental PET hydrolase activity. Each sequence was searched against the families in ESTHER database with HMMER (http://bioweb.supagro.inra.fr/ESTHER/general?what=index). The top ESTHER family returned by the search, the search E-value, and the associated EC number are reported. All sequence candidates sourced from NCBI with high HMM scores fall into the polyesterase-lipase-cutinase family with E-values of 1.0e-45 or better, as is typical of canonical PET hydrolases. Sequence candidates sourced from JGI IMG with lower HMM scores fall into different ESTHER families and are associated with carboxyl ester hydrolase (3.1.1.-) or peptidase (3.4.-.-) activities. Each sequence was also searched against the SwissProt database using BLAST and the best hit with annotated E.C number is shown. The deep-learning method, DeepEC, was additionally applied to predict E.C. numbers of the sequence and the E.C number with the highest predicted probability is reported.

	Enzyme ID	Top ESTHER family	ESTHER E-value	ESTHER EC number	E-value of SwissProt top hit	EC number of SwissProt top hit	DeepEC predicted EC number
1	101	Carb_B_root	1.7e-86	3.1.1.1	5.0e-49	3.1.1.87	3.1.1.-
2	102	Carb_B_Bacteria	6.2e-101	3.1.1.1	1.9e-65	3.1.1.87	3.1.1.-
3	202	Xaa-Pro_like_dom	1.0e-52	3.4.14.11	5.1e-17	3.1.-.-	3.7.1.-
4	204	5_AlphaBeta_hydrolase	1.9E-29	-	7.8E-08	3.1.1.23	2.3.1
5	211	Abhydrolase_5	2E-33	-	3.0E-09	3.4.21.-	3.1.1.-
6	214	Xaa-Pro_like_dom	1.3E-45	3.4.14.11	3.0e-17	3.1.-.-	4.2.99
7	301	Chlorophyllase	2.7e-27	3.1.1.14	3.3e-12	3.1.1.74	3.1.1.-
8	305	Duf_1400	8.3e-68	-	1.8e-04	3.1.1.14	3.1.1.-
9	307	Duf_1100-S	6.4e-22	-	3.1e-05	3.4.14.-	3.1.1.-
10	401	Polyesterase-lipase-cutinase	4.40E-97	3.1.1.74	4.10E-88	3.1.1.74	3.1.1.-
11	403	Polyesterase-lipase-cutinase	8.30E-105	3.1.1.74	4.40E-88	3.1.1.74	3.1.1.-
12	405	Polyesterase-lipase-cutinase	1.10E-106	3.1.1.74	1.30E-80	3.1.1.101	3.1.1.-
13	406	Polyesterase-lipase-cutinase	5.20E-92	3.1.1.74	5.60E-84	3.1.1.74	3.1.1.-
14	407	Polyesterase-lipase-cutinase	7.10E-88	3.1.1.74	4.50E-64	3.1.1.101	3.1.1.-
15	409	Polyesterase-lipase-cutinase	1.30E-48	3.1.1.74	3.10E-40	3.1.1.74	3.1.1.-
16	412	Polyesterase-lipase-cutinase	1.60E-45	3.1.1.74	1.40E-38	3.1.1.74	3.1.1.-
17	501	Polyesterase-lipase-cutinase	6.10E-120	3.1.1.74	0.0	3.1.1.101	3.1.1.-
18	503	Polyesterase-lipase-cutinase	3.50E-105	3.1.1.74	1.70E-89	3.1.1.101	3.1.1.-
19	504	Polyesterase-lipase-cutinase	7.30E-101	3.1.1.74	1.40E-90	3.1.1.74	3.1.1.-
20	601	Polyesterase-lipase-cutinase	2.00E-134	3.1.1.74	1.10E-98	3.1.1.101	3.1.1.-
21	602	Polyesterase-lipase-cutinase	2.40E-130	3.1.1.74	1.10E-98	3.1.1.101	3.1.1.-
22	606	Polyesterase-lipase-cutinase	7.60E-122	3.1.1.74	9.70E-95	3.1.1.101	3.1.1.-
23	607-nSP	Polyesterase-lipase-cutinase	4.20E-133	3.1.1.74	1.30E-97	3.1.1.101	3.1.1.-
24	611	Polyesterase-lipase-cutinase	1.50E-117	3.1.1.74	1.20E-87	3.1.1.101	3.1.1.-
25	701	Polyesterase-lipase-cutinase	7.10E-136	3.1.1.74	7.00E-102	3.1.1.101	3.1.1.-
26	702	Polyesterase-lipase-cutinase	2.30E-135	3.1.1.74	1.50E-103	3.1.1.101	3.1.1.-
27	703	Polyesterase-lipase-cutinase	6.50E-136	3.1.1.74	1.40E-101	3.1.1.101	3.1.1.-
28	704	Polyesterase-lipase-cutinase	2.50E-136	3.1.1.74	9.90E-102	3.1.1.101	3.1.1.-
29	705	Polyesterase-lipase-cutinase	1.10E-136	3.1.1.74	2.00E-101	3.1.1.101	3.1.1.-
30	706	Polyesterase-lipase-cutinase	1.10E-136	3.1.1.74	4.00E-101	3.1.1.101	3.1.1.-
31	707	Polyesterase-lipase-cutinase	4.40E-132	3.1.1.74	2.00E-101	3.1.1.101	3.1.1.-
32	708	Polyesterase-lipase-cutinase	1.10E-137	3.1.1.74	5.60E-106	3.1.1.101	3.1.1.-
33	709	Polyesterase-lipase-cutinase	1.30E-132	3.1.1.74	8.60E-103	3.1.1.101	3.1.1.-
34	711	Polyesterase-lipase-cutinase	1.50E-128	3.1.1.74	4.20E-98	3.1.1.101	3.1.1.-
35	714	Polyesterase-lipase-cutinase	2.10E-136	3.1.1.74	8.90E-103	3.1.1.101	3.1.1.-
36	715	Polyesterase-lipase-cutinase	2.20E-136	3.1.1.74	2.10E-103	3.1.1.101	3.1.1.-
37	716	Polyesterase-lipase-cutinase	6.20E-137	3.1.1.74	3.90E-102	3.1.1.101	3.1.1.-
38	717	Polyesterase-lipase-cutinase	5.90E-137	3.1.1.74	3.70E-104	3.1.1.101	3.1.1.-

Supplementary Table 11. Expression and purification trial results for all 74 enzymes and the signal peptide-containing variants. Enzymes previously reported to have PET hydrolysis activity are shown in peach. Constructs that were not expressed sufficiently for screening are shown in grey. Expression yields are also shown in **Supplementary Figure 2**. Candidates noted nSP encode the native signal peptide sequence at the N-terminus of the expression sequence. The expression approaches employed are described in the Materials and Methods, annotated as strategies A-D. Briefly, in strategy A, induction in 2xYT media with 1 mM IPTG at 20°C was used; in strategy B, induction in 2xYT media with 0.5 mM IPTG at 25°C was used; in strategy C, autoinduction in ZYP-5052 media at 28°C was used; and in strategy D, autoinduction in ZYP-5052 media supplemented with 0.3 M NaCl at 25°C was used. The final concentration of protein per L of culture is reported after affinity and size exclusion chromatography. Also reported is the pH and temperature combination that resulted in the highest level of product release from the screening assays. C6 = citrate, pH 6; NP7 = NaH_2PO_4, pH 7; NP7.5 = NaH_2PO_4, pH 7.5; H7.5 = HEPES, pH 7.5; B8 = bicine, pH 8; G9 = glycine, pH 9. The enzyme loading (in µg enzyme per reaction) for each screening reaction is noted.

	Enzyme ID	Competent Cell Line	Expression Strategy	Expression Level (mg/L)	Activity optimum (pH / Temperature)	Enzyme loading (µg/rxn)
1	101	BL21 (DE3)	C	1.8	G9 / 70°C	10
2	102	BL21 (DE3)	B	0.9	C6 / 60°C	10
3	103	BL21 (DE3)	C	0.2	No Activity	5
4	104					
5	201					
6	202	BL21 (DE3)	A	110	C6 / 70°C	10
7	203			insoluble		
8	204	BL21 (DE3)	C	0.3	B8 / 70°C	3
9	205					
10	206	C41 (DE3)	D	0.2	No Activity	2
11	207	BL21 (DE3), C41 (DE3)		<0.1		
12	208	BL21 (DE3), C41 (DE3)		<0.1		
13	209	BL21 (DE3)	B	1.9	No Activity	10
14	210			insoluble		
15	211	BL21 (DE3)	C	2.6	NP7.5 / 70°C	10
16	212					
17	213					
18	214	BL21 (DE3)	C	1.1	G9 / 50°C	10
19	215	BL21 (DE3)	C	88	No Activity	10
20	301	BL21 (DE3)	B	2.9	C6 / 70°C	10
21	302	BL21 (DE3), C41 (DE3)		<0.1		
22	303					
23	304					
24	305	BL21 (DE3)	A	0.3	C6 / 70°C	2
25	306	BL21 (DE3)	C	17.4	No Activity	10

26	307	C41 (DE3)	C	4.1	G9 / 60°C	10
27	308					
28	401	BL21 (DE3)	A	1.1	NP7.5 / 70°C	10
29	402	BL21 (DE3)	C	0.1	No Activity	3
30	403	BL21 (DE3)	C	2.5	G9 / 70°C	10
31	404					
32	405	BL21 (DE3)	A	2.8	G9 / 70°C	10
33	406	BL21 (DE3)	C	1.6	G9 / 70°C	10
34	407	BL21 (DE3)	A	3.2	G9 / 50°C	10
35	408					
36	409	BL21 (DE3)	A	5.6	G9 / 60°C	10
37	410	BL21 (DE3)	C	8.4	No Activity	10
38	411					
39	412	BL21 (DE3)	A	3.2	C6 / 60°C	10
40	413			insoluble		
41	501	C41 (DE3)	C	10.1	NP7.5 / 60°C	10
42	502					
43	503	BL21 (DE3)	A	5.1	G9 / 30°C	10
44	504	BL21 (DE3)	A	1.7	B8 / 50°C	10
45	505					
46	601	BL21 (DE3)	C	1.1	NP7.5 / 60°C	10
47	602	BL21 (DE3)	C	0.9	H7.5 / 60°C	10
48	603			insoluble		
49	604	BL21 (DE3)	D	0.6	No Activity	10
50	605	BL21 (DE3)	C	2.1	No Activity	10
51	606	C41 (DE3)	C	6	G9 / 60°C	10
52	607					
53	608	BL21 (DE3)	A	2.7	No Activity	10
54	609					
55	610	BL21 (DE3)	A	1.4	No Activity	10
56	611	BL21 (DE3)	A	31.1	C6 / 50°C	10
57	612					
58	701	C41 (DE3)	A	0.7	NP7.5 / 60°C	10

59	702	BL21 (DE3)	C	105.7	C6 / 50°C	10
60	703	BL21 (DE3)	A	45.9	NP7.5 / 60°C	10
61	704	BL21 (DE3)	B	41.4	NP7 / 60°C	10
62	705	BL21 (DE3)	A	97.3	NP7.5 / 60°C	10
63	706	BL21 (DE3)	C	50.5	NP7.5 / 60°C	10
64	707	BL21 (DE3)	B	16.3	C6 / 70°C	10
65	708	BL21 (DE3)	A	65.4	NP7 / 40°C	10
66	709	BL21 (DE3)	C	88.2	No Activity	10
67	710	BL21 (DE3)	B	2.2	No Activity	10
68	711	BL21 (DE3)	A	40.8	NP7 / 30*C	10
69	712	BL21 (DE3)	A	3.6	No Activity	10
70	713	BL21 (DE3)	C	11.6	No Activity	10
71	714	BL21 (DE3)	C	24.8	NP7 / 60°C	10
72	715	BL21 (DE3)	B	125.1	C6 / 60°C	10
73	716	BL21 (DE3)	C	38.3	NP7.5 / 60°C	10
74	717	BL21 (DE3)	B	5.8	C6 / 70°C	10
75	102-nSP					
76	301-nSP	C41 (DE3)	D	0.3	No Activity	3
77	401-nSP	C41 (DE3)	C	15	NP7 / 50°C	10
78	402-nSP					
79	403-nSP	C41 (DE3)	C	20.6	H7.5 / 70°C	10
80	410-nSP	C41 (DE3)	D	5	No Activity	10
81	505-nSP					
82	603-nSP			insoluble		
83	606-nSP	C41 (DE3)	C	2.5	C6 / 70°C	10
84	607-nSP	C41 (DE3)	A	4.4	B8 / 50°C	10
85	610-nSP			insoluble		
86	711-nSP	C41 (DE3)	D	5.7	No Activity	10

Supplementary Table 12. Enzymes and reaction conditions tested in 168 h time course experiments using two PET substrate morphologies, either amorphous PET film (A) or crystalline PET powder (C). Selectivity ratio provides the mass ratio of measured aromatic products at 168 h, and which substrate experienced higher levels of hydrolysis is noted (A or C). Reaction conditions tested that are not shown in **Supplementary Figure 5** are noted with an asterisk (*).

	**Enzyme ID**	**Reaction Condition (pH / Temperature)**	**Selectivity Ratio at 168 h (mass ratio)**
1	BTA-1	H7.5 / 60°C	8.05 (A)
2	LCC_WT	NP7.5 / 60°C	3.67 (A)
3	LCC ICCG	NP7.5 / 70°C	4.56 (A)
4	LCC ICCG	C6 / 60°C (*)	5.08 (A)
5	102	C6 / 60°C	7.84 (C)
6	202	NP7.5 / 70°C	1.46 (C)
7	211	NP7.5 / 70°C	1.24 (A)
8	407	G9 / 50°C	1.23 (C)
9	504	B8 / 50°C	5.64 (C)
10	601	NP7.5 / 60°C	1.86 (C)
11	606	G9 / 60°C	3.30 (C)
12	606	NP7.5 / 60°C (*)	3.33 (C)
13	611	C6 / 50°C	1.24 (C)
14	611	NP7.5 / 50°C (*)	10.31 (C)
15	701	NP7.5 / 60°C	4.73 (A)
16	704	NP7 / 60°C	7.41 (A)
17	704	NP7.5 / 60°C (*)	10.46 (A)
18	714	NP7 / 60°C	1.95 (A)
19	716	NP7.5 / 60°C	3.08 (A)

Supplementary Table 13. Optimal reaction conditions identified by screening across pH and temperature on three PET substrate morphologies. Reaction conditions tested for 168 h time course experiments using three PET substrate morphologies [either amorphous PET film (aFilm), amorphous PET powder (aPow), or crystalline PET powder (cryPow)] are indicated in grey. Selectivity ratio provides the mass ratio of measured aromatic products at 168 h, and which substrate experienced higher levels of hydrolysis is noted (aFilm, aPow, or cryPow). Reactions and reaction conditions tested that are not shown in **Figure 3B** (main text) are noted with an asterisk (*). The Ø symbol indicates zero product quantitation in either the numerator or denominator of the ratio calculation.

	Enzyme ID	Optimal Reaction Condition (pH / Temperature)			Selectivity Ratio at 168 h (mass ratio)		
		aFilm	aPow	cryPow	aPow v. cryPow	aPow v. aFilm	cryPow v. aFilm
1	LCC ICCG	NP7.5 / 70°C	NP7.5 / 70°C	NP7 / 60°C	5.25 (aPow)	1.15 (aPow)	4.56 (aFilm)
2	204	B8 / 70°C	NP7 / 50°C	H7.5 / 50°C	1.84 (aPow)	7.37 (aPow)	4.00 (cryPow)
3	211	NP7.5 / 70°C	C6 / 60°C	C6 / 60°C	3.37 (aPow)	5.13 (aPow)	1.52 (cryPow)
4	214	G9 / 50°C (*)	G9 / 70°C	G9 / 70°C	Ø	Ø	1.56 (cryPow)
5	301	C6 / 70°C (*)	NP7 / 70°C	G9 / 70°C	2.63 (aPow)	2.30 (aPow)	1.14 (aFilm)
6	307	NP7 / 70°C (*)	H7.5 / 70°C		4.36 (aPow)	8.81 (aPow)	2.02 (cryPow)
				C6 / 60°C (*)	Ø	Ø	Ø
7	401	NP7.5 / 70°C (*)	NP7.5 / 60°C	G9 / 50°C	1.89 (aPow)	235.35 (aPow)	124.68 (cryPow)
8	405	G9 / 70°C (*)	G9 / 70°C (*)	G9 / 70°C (*)	2.65 (aPow)	3.22 (aPow)	1.21 (cryPow)
9	503	G9 / 30°C	B8 / 40°C	B8 / 40°C	1.30 (cryPow)	436.1 (aPow)	569.10 (cryPow)
10	504	B8 / 50°C	B8 / 50°C	G9 / 50°C	3.44 (aPow)	55.92 (aPow)	16.25 (cryPow)
11	601	NP7.5 / 60°C	B8 / 50°C	G9 / 60°C	1.51 (aPow)	1.43 (aPow)	1.05 (aFilm)
12	602	H7.5 / 60°C (*)	G9 / 40°C	G9 / 40°C	2.79 (cryPow)	9.96 (aPow)	27.80 (cryPow)
13	611	C6 / 50°C			2.27 (aPow)	2.82 (aPow)	1.24 (cryPow)
			NP7.5 / 50°C (*)	NP7.5 / 50°C (*)	2.32 (aPow)	23.97 (aPow)	10.31 (cryPow)
14	701	NP7.5 / 60°C	NP7.5 / 60°C	G9 / 60°C	2.24 (aPow)	1.61 (aFilm)	3.60 (aFilm)
15	704	NP7.5 / 60°C	B8 / 60°C	NP7.5 / 60°C	3.21 (aPow)	3.26 (aFilm)	10.46 (aFilm)
16	708	NP7 / 40°C (*)	NP7 / 50°C	NP7 / 50°C	1.24 (aPow)	34.82 (aPow)	28.10 (cryPow)
17	711	NP7 / 30°C (*)	NP7.5 / 60°C	NP7 / 40°C	2.13 (cryPow)	Ø	Ø
18	714	NP7 / 60°C	NP7.5 / 60°C	G9 / 50°C	2.45 (aPow)	1.26 (aPow)	1.95 (aFilm)
19	717	C6 / 70°C (*)	G9 / 60°C	G9 / 60°C	8.85 (aPow)	85.20 (aPow)	9.63 (cryPow)

Supplementary Table 14. T_m data for selected proteins.

**Enzyme ID**	**Mean T_m (°C)**	**T_m s.d. (°C)**	**Buffer**
102	65.96	± 0.28	NP7.5
202	75.13	± 0.06	NP7.5
306	92.57	± 0.02	NP7.5
407	68.20	± 0.04	NP7.5
501	86.91	± 0.12	NP7.5
504	67.25	± 0.03	NP7.5
601	67.18	± 0.04	NP7.5
606	53.90	± 0.11	NP7.5 + 0.3 M NaCl
611	76.21	± 0.05	NP7.5
701	70.28	± 0.03	NP7.5
702	65.57	± 0.03	NP7.5
703	70.86	± 0.09	NP7.5
704	69.93	± 0.08	NP7.5
705	69.02	± 0.05	NP7.5
706	68.35	± 0.10	NP7.5
709	56.05	± 0.05	NP7.5
711	54.16	± 0.03	NP7.5
714	69.96	± 0.08	NP7.5
715	71.83	± 0.03	NP7.5
716	67.71	± 0.15	NP7.5
BTA-1	71.94	± 0.03	NP7.5

Supplementary Table 15. Crystallographic data and model refinement statistics

	**202**	**306**	**606**	**611**
**Data collection**				
Beamline	DLS I24	DLS I03	DLS I03	DLS I03
Space group	*I*222	$P3_221$	$P2_1$	*P*321
Cell dimensions				
a, b, c (Å)	90.7 / 124.9 / 167.0	74.9 / 74.9 / 116.5	74.0 / 45.4 / 86.6	89.3 / 89.3 / 74.3
α, β, γ (°)	90.0 / 90.0 / 90.0	90.0 / 90.0 / 120. 0	90.0 / 108.2 / 90.0	90.0 / 90.0 / 120.0
Resolution (Å)	50.33 – 2.19	64.88 – 1.89	82.27 – 1.93	77.37 – 1.56
Rmerge [%]	8.9 (318.1)	13.5 (184.1)	25.3 (134.3)	14.0 (215.3)
Rpim [%]	2.6 (89.3)	3.3 (46.5)	11.4 (55.5)	3.4 (54.8)
<I/σI>	14.6 (0.9)	14.8 (1.7)	5.5 (1.4)	12.5 (1.4)
Completeness (%)	100.0 (100.0)	93.7 (76.6)[a]	91.6 (52.0)[a]	94.9 (64.7)[a]
Redundancy	13.1 (13.5)	17.7 (16.5)	5.9 (6.7)	17.4 (16.4)
CC(1/2)	0.999 (0.292)	0.999 (0.634)	0.988 (0.577)	0.999 (0.632)
**Refinement**				
Rwork / Rfree	21.6 / 24.4	21.1 / 25.1	23.2 / 28.2	14.3 / 17.1
Ramachandran plot				
most favored [%]	96.6	97.0	94.2	97.2
allowed [%]	2.9	3.0	5.2	2.8
disallowed [%]	0.5	0.0	0.6	0.0
No. atoms				
protein	4629	2224	4044	1969
water	139	94	248	314
PEG				13
B-factors				
protein	80.1	38.5	20.4	19.2
water	66.7	35.6	21.1	38.3
PEG				46.9
R.m.s. deviations				
Bond lengths (Å)	0.0117	0.0128	0.0110	0.0164
Bond angles (°)	1.57	1.54	1.74	2.01
PDB ID	7QJM	7QJN	7QJO	7QJP

	702	703	705	711
**Data collection**				
Beamline	DLS I03	DLS I24	DLS I04-1	DLS I03
Space roup	*P*1	$P3_221$	$P2_1$	$P4_32_12$
Cell dimensions				
a, b, c (Å)	40.3 / 79.8 / 120.1	71.7 / 71.7 / 102.5	36.2 / 150.2 / 43.4	109.3 / 109.3 / 44.2
α, β, γ (°)	86.3 / 87.9 / 89.5	90.0 / 90.0 / 120.0	90.0 / 92.5 / 90.0	90.0 / 90.0 / 90.0
Resolution (Å)	119.80 – 1.64	62.08 – 1.51	75.11 – 1.43	77.29 – 1.78
Rmerge [%]	9.0 (77.2)	11.5 (368.2)	12.1 (117.0)	63.9 (312.7)
Rpim [%]	5.5 (48.1)	2.7 (85.2)	4.9 (49.3)	12.6 (65.0)
<I/σI> [a]	9.0 (1.5)	14.5 (0.9)	9.3 (1.5)	6.3 (1.6)
Completeness (%)	89.5 (53.6) [a]	100.0 (100.0)	89.0 (55.7) [a]	87.1 (64.3) [a]
Redundancy	3.6 (3.6)	19.4 (19.5)	6.9 (6.4)	26.2 (22.0)
CC(1/2)	0.998 (0.567)	0.999 (0.350)	0.997 (0.567)	0.992 (0.615)
**Refinement**				
Rwork / Rfree	16.5 / 19.9	15.4 / 18.6	18.2 / 21.6	18.2 / 24.2
Ramachandran plot				
most favored [%]	97.8	98.1	98.0	96.2
allowed [%]	2.2	1.9	2.0	3.4
disallowed [%]	0.0	0.0	0.0	0.4
No. atoms				
protein	12006	2044	3994	2044
water	1515	222	372	241
PEG	42	10	29	19
B-factors				
protein	21.3	22.3	16.5	19.0
water	31.7	39.1	26.8	29.2
PEG	50.5	50.5	38.1	32.0
R.m.s. deviations				
Bond lengths (Å)	0.0125	0.0166	0.0145	0.0120
Bond angles (°)	1.63	2.05	1.86	1.78
PDB ID	7QJQ	7QJR	7QJS	7QJT

[a] ellipsoidal completeness

Supplementary References

1. F. W. Studier, "Stable expression clones and auto-induction for protein production in *E. coli*" in Structural Genomics. (Springer, 2014), pp. 17-32.
2. P. Liu, H. E. Ewis, P. C. Tai, C.-D. Lu, I. T. Weber, Crystal structure of the *Geobacillus stearothermophilus* carboxylesterase Est55 and its activation of prodrug CPT-11. *J Mol Biol* **367**, 212-223 (2007).
3. D. Ribitsch *et al.*, Hydrolysis of polyethyleneterephthalate by p-nitrobenzylesterase from *Bacillus subtilis*. *Biotechnology Progress* **27**, 951-960 (2011).
4. B. C. Knott *et al.*, Characterization and engineering of a two-enzyme system for plastics depolymerization. *PNAS* **117**, 25476-25485 (2020).
5. V. Tournier *et al.*, An engineered PET depolymerase to break down and recycle plastic bottles. *Nature* **580**, 216-219 (2020).
6. S. Yoshida *et al.*, A bacterium that degrades and assimilates poly (ethylene terephthalate). *Science* **351**, 1196-1199 (2016).
7. H. P. Austin *et al.*, Characterization and engineering of a plastic-degrading aromatic polyesterase. *PNAS* **115**, E4350-E4357 (2018).
8. R. J. Müller, H. Schrader, J. Profe, K. Dresler, W. D. Deckwer, Enzymatic degradation of poly (ethylene terephthalate): rapid hydrolyse using a hydrolase from *T. fusca*. *Macromol Rapid Commun* **26**, 1400-1405 (2005).
9. C. Silva *et al.*, Engineered *Thermobifida fusca* cutinase with increased activity on polyester substrates. *Biotechnol J* **6**, 1230-1239 (2011).
10. A. Eberl *et al.*, Enzymatic surface hydrolysis of poly (ethylene terephthalate) and bis (benzoyloxyethyl) terephthalate by lipase and cutinase in the presence of surface active molecules. *J Biotechnol* **143**, 207-212 (2009).
11. S. Chen *et al.*, Identification and characterization of bacterial cutinase. *J Biol Chem* **283**, 25854-25862 (2008).
12. I. Kleeberg, K. Welzel, J. VandenHeuvel, R.-J. Müller, W.-D. Deckwer, Characterization of a new extracellular hydrolase from *Thermobifida fusca* degrading aliphatic– aromatic copolyesters. *Biomacromolecules* **6**, 262-270 (2005).
13. K. Dresler, J. van den Heuvel, R.-J. Müller, W.-D. Deckwer, Production of a recombinant polyester-cleaving hydrolase from *Thermobifida fusca* in *Escherichia coli*. *Bioprocess Biosyst Eng* **29**, 169-183 (2006).
14. J. Then *et al.*, Ca^{2+} and Mg^{2+} binding site engineering increases the degradation of polyethylene terephthalate films by polyester hydrolases from *Thermobifida fusca*. *Biotechnol J* **10**, 592-598 (2015).
15. A. N. Shirke *et al.*, Stabilizing leaf and branch compost cutinase (LCC) with glycosylation: mechanism and effect on PET hydrolysis. *Biochemistry* **57**, 1190-1200 (2018).
16. S. Sulaiman *et al.*, Isolation of a novel cutinase homolog with polyethylene terephthalate-degrading activity from leaf-branch compost by using a metagenomic approach. *Appl Env Microbiol* **78**, 1556-1562 (2012).
17. R. Araújo *et al.*, Tailoring cutinase activity towards polyethylene terephthalate and polyamide 6, 6 fibers. *J Biotechnol* **128**, 849-857 (2007).
18. S. Longhi, M. Czjzek, V. Lamzin, A. Nicolas, C. Cambillau, Atomic resolution (1.0 Å) crystal structure of *Fusarium solani* cutinase: stereochemical analysis. *J Mol Biol* **268**, 779-799 (1997).
19. Å. M. Ronkvist, W. Xie, W. Lu, R. A. Gross, Cutinase-catalyzed hydrolysis of poly (ethylene terephthalate). *Macromolecules* **42**, 5128-5138 (2009).
20. E. Herrero Acero *et al.*, Enzymatic surface hydrolysis of PET: effect of structural diversity on kinetic properties of cutinases from *Thermobifida*. *Macromolecules* **44**, 4632-4640 (2011).
21. E. Herrero Acero *et al.*, Surface engineering of a cutinase from *Thermobifida cellulosilytica* for improved polyester hydrolysis. *Biotechnol Bioeng* **110**, 2581-2590 (2013).
22. D. Ribitsch *et al.*, Characterization of a new cutinase from *Thermobifida alba* for PET-surface hydrolysis. *Biocatal Biotrans* **30**, 2-9 (2012).
23. D. Ribitsch *et al.*, A new esterase from *Thermobifida halotolerans* hydrolyses polyethylene terephthalate (PET) and polylactic acid (PLA). *Polymers* **4**, 617-629 (2012).
24. F. Kawai *et al.*, A novel Ca^{2+}-activated, thermostabilized polyesterase capable of hydrolyzing polyethylene terephthalate from *Saccharomonospora viridis* AHK190. *Appl Microbiol Biotechnol* **98**, 10053-10064 (2014).
25. R. Wei *et al.*, Functional characterization and structural modeling of synthetic polyester-degrading hydrolases from *Thermomonospora curvata*. *AMB Express* **4**, 1-10 (2014).
26. D. Danso *et al.*, New insights into the function and global distribution of polyethylene terephthalate (PET)-degrading bacteria and enzymes in marine and terrestrial metagenomes. *Appl Env Microbiol* **84**, e02773-02717 (2018).
27. A. Nakamura, N. Kobayashi, N. Koga, R. Iino, Positive charge introduction on the surface of thermostabilized PET hydrolase facilitates PET binding and degradation. *ACS Catal* **11**, 8550-8564 (2021).

28. M. Furukawa, N. Kawakami, A. Tomizawa, K. Miyamoto, Efficient degradation of poly (ethylene terephthalate) with *Thermobifida fusca* cutinase exhibiting improved catalytic activity generated using mutagenesis and additive-based approaches. *Sci Rep* **9**, 1-9 (2019).
29. J. Schmidt *et al.*, Effect of Tris, MOPS, and phosphate buffers on the hydrolysis of polyethylene terephthalate films by polyester hydrolases. *FEBS Open Bio* **6**, 919-927 (2016).
30. M. Barth *et al.*, Effect of hydrolysis products on the enzymatic degradation of polyethylene terephthalate nanoparticles by a polyester hydrolase from *Thermobifida fusca*. *Biochem Eng J* **93**, 222-228 (2015).
31. K. Hegde, V. D. Veeranki, Production optimization and characterization of recombinant cutinases from *Thermobifida fusca* sp. NRRL B-8184. *Appl Biochem Biotechnol* **170**, 654-675 (2013).
32. R. Jabloune *et al.*, Enzymatic degradation of *p*-nitrophenyl esters, polyethylene terephthalate, cutin, and suberin by Sub1, a suberinase encoded by the plant pathogen *Streptomyces scabies*. *Microbes Environ* **35**, ME19086 (2020).
33. V. Perz *et al.*, Hydrolysis of synthetic polyesters by *Clostridium botulinum* esterases. *Biotechnol Bioeng* **113**, 1024-1034 (2016).
34. X. Xi *et al.*, Secretory expression in *Bacillus subtilis* and biochemical characterization of a highly thermostable polyethylene terephthalate hydrolase from bacterium HR29. *Enzyme Microb Technol* **143**, 109715 (2021).
35. A. Bollinger *et al.*, A novel polyester hydrolase from the marine bacterium *Pseudomonas aestusnigri*–Structural and functional insights. *Front Microbiol* **11**, 114 (2020).
36. Z. Hongli *et al.*, The abundance of mRNA transcripts of bacteroidetal polyethylene terephthalate (PET) esterase genes may indicate a role in marine plastic degradation. *Research Square* (2021).
37. C. Sonnendecker *et al.*, Low carbon footprint recycling of post-consumer PET plastic with a metagenomic polyester hydrolase. *ChemSusChem* **15**, 9 (2021).
38. X. Hu, U. Thumarat, X. Zhang, M. Tang, F. Kawai, Diversity of polyester-degrading bacteria in compost and molecular analysis of a thermoactive esterase from *Thermobifida alba* AHK119. *Appl Microbiol Biotechnol* **87**, 771-779 (2010).

Consortium

John Jumper[1], Richard Evans[1], Alexander Pritzel[1], Tim Green[1], Michael Figurnov[1], Olaf Ronneberger[1], Kathryn Tunyasuvunakool[1], Russ Bates[1], Augustin Žídek[1], Anna Potapenko[1], Alex Bridgland[1], Clemens Meyer[1], Simon A. A. Kohl[1], Andrew J. Ballard[1], Andrew Cowie[1], Bernardino Romera-Paredes[1], Stanislav Nikolov[1], Rishub Jain[1], Jonas Adler[1], Trevor Back[1], Stig Petersen[1], David Reiman[1], Ellen Clancy[1], Michal Zielinski[1], Tamas Berghammer[1], Sebastian Bodenstein[1], David Silver[1], Oriol Vinyals[1], Andrew W. Senior[1], Koray Kavukcuoglu[1], Pushmeet Kohli[1], and Demis Hassabis[1].

1. AlphaFold Team, DeepMind, London, UK,
