# Supplementary material for: Sourcing thermotolerant poly(ethylene terephthalate) hydrolase scaffolds from natural diversity
Source: Nat Commun. 2022 Dec 21;13:7850. doi: 10.1038/s41467-022-35237-x (PMC9772341; doi:10.1038/s41467-022-35237-x)
Supplement: Supplementary file 1 — Peer Review File [file 41467_2022_35237_MOESM1_ESM.pdf]

nature portfolio

Peer Review File

Structural transition and re-emergence of iron's total electron spin in (Mg,Fe)O at ultrahigh pressure

Open Access This file is licensed under a Creative Commons Attribution 4.0 International License, which permits use, sharing, adaptation, distribution and reproduction in any medium or format, as long as you give appropriate credit to the original author(s) and the source, provide a link to the Creative Commons license, and indicate if changes were made. In the cases where the authors are anonymous, such as is the case for the reports of anonymous peer reviewers, author attribution should be to 'Anonymous Referee' followed by a clear attribution to the source work. The images or other third party material in this file are included in the article's Creative Commons license, unless indicated otherwise in a credit line to the material. If material is not included in the article's Creative Commons license and your intended use is not permitted by statutory regulation or exceeds the permitted use, you will need to obtain permission directly from the copyright holder. To view a copy of this license, visit http://creativecommons.org/licenses/by/4.0/.

Editorial Note: This manuscript has been previously reviewed at another journal that is not operating a transparent peer review scheme. This document only contains reviewer comments and rebuttal letters for versions considered at Nature Communications .

REVIEWERS' COMMENTS

Reviewer #4 (Remarks to the Author):

The authors have responded to the comments and modified the manuscript accordingly.
Since the data shown to prove the identification of novel enzymes preferentially hydrolyzing crystalline PET are still limited in my opinion, this finding will certainly be the subject of further investigation as also suggested in the authors' reply. The authors remarked that they purposely placed the interpretation of these results in the Discussion section ("Also, we note that most of the "interpretation" is in the Discussion section, which is completely separated (intentionally) from the Results").
The revised manuscript provides interesting and new information on the diversity of PET hydrolases and is recommended for publication.
